# Health service utilisation during the COVID-19 pandemic in sub-Saharan Africa in 2020: a multicountry empirical assessment with a focus on maternal, newborn and child health services

**DOI:** 10.1136/bmjgh-2021-008069

**Published:** 2022-05-02

**Authors:** Agbessi Amouzou, Abdoulaye Maïga, Cheikh Mbacké Faye, Samuel Chakwera, Dessalegn Y Melesse, Martin Kavao Mutua, Sokhna Thiam, Idrissa Boukary Abdoulaye, Seth Kwaku Afagbedzi, Akory Ag Iknane, Odile Sassor Ake-Tano, Joshua O Akinyemi, Victor Alegana, Yakubu Alhassan, Arinaitwe Emma Sam, Dominic Kwabena Atweam, Shraddha Bajaria, Luke Bawo, Mamadou Berthé, Andrea Katryn Blanchard, Hamissou Alaji Bouhari, Ousmane Maimouna Ali Boulhassane, Maio Bulawayo, Ovost Chooye, Amed Coulibaly, Mamatou Diabate, Fatou Diawara, Ousman Esleman, Mulugeta Gajaa, Kamil Halimatou Amadou Garba, Theodros Getachew, Choolwe Jacobs, George P Jacobs, Femi James, Ayodele S Jegede, Catherine Joachim, Rornald Muhumuza Kananura, Janette Karimi, Helen Kiarie, Denise Kpebo, Bruno Lankoandé, Akanni Olayinka Lawanson, Yahaha Mahamadou, Masoud Mahundi, Tewabe Manaye, Honorati Masanja, Modeste Roch Millogo, Abdoul Karim Mohamed, Mwiche Musukuma, Rose Muthee, Douba Nabié, Mukome Nyamhagata, Jimmy Ogwal, Adebola Orimadegun, Ajiwohwodoma Ovuoraye, Adama Sanogo Pongathie, Stéphane Parfait Sable, Geetor S Saydee, Josephine Shabini, Brivine Mukombwe Sikapande, Daudi Simba, Ashenif Tadele, Tefera Tadlle, Alfred K Tarway-Twalla, Mahamadi Tassembedo, Bentoe Zoogley Tehoungue, Ibrahim Terera, Soumaïla Traoré, Musu P Twalla, Peter Waiswa, Naod Wondirad, Ties Boerma

**Affiliations:** 1 Department of International Health, Johns Hopkins University Bloomberg School of Public Health, Baltimore, Maryland, USA; 2 African Population Health Research Centre, Dakar, Senegal; 3 School of Public Health, University of Witwatersrand, Johannesburg, Gauteng, South Africa; 4 UNICEF, New York, New York, USA; 5 Community Health Science, University of Manitoba, Winnipeg, Manitoba, Canada; 6 African Population and Health Research Center, Nairobi, Kenya; 7 Institut National de la Statistique, Niamey, Niger; 8 University of Ghana School of Public Health, Accra, Greater Accra, Ghana; 9 Institut National de Santé Publique, Bamako, Mali; 10 Institut National de Santé Publique, Abidjan, Côte d'Ivoire; 11 University of Ibadan, Ibadan, Oyo, Nigeria; 12 School of Geography and Environmental Sciences, University of Southampton, Southampton, UK; 13 Kenya Medical Research Institute, Nairobi, Kenya; 14 Ministry of Health, Kampala, Uganda; 15 Ghana Health Service, Accra, Greater Accra, Ghana; 16 Ifakara Health Institute, Ifakara, Morogoro, Tanzania, United Republic of; 17 Ministry of Health, Monrovia, Montserrado, Liberia; 18 Department of Health Policy and Management, University of Zambia, Lusaka, Zambia; 19 Zambia Ministry of Health, Lusaka, Zambia; 20 Ministère de la Santé et de l'Hygiène Publique du Mali, Bamako, Mali; 21 Federal Ministry of Health, Addis Ababa, Ethiopia; 22 Health System and Reproductive Health Research Directorate, Ethiopian Public Health Institute, Addis Ababa, Oromia, Ethiopia; 23 College of Medicine and Health Science, Institute of Public Health, University of Gondar College of Medicine and Health Sciences, Gondar, Ethiopia; 24 Epidemiology and Biostatistics, University of Zambia, Lusaka, Zambia; 25 Ministry of Health, Abuja, Nigeria; 26 Ministry of Health, Dar Es Salaam, Tanzania, United Republic of; 27 School of Public Health, Makerere University, Kampala, Uganda; 28 Ministry of Health, Nairobi, Kenya; 29 Institut Superieur des Sciences de la Population, Ouagadougou, Centre, Burkina Faso; 30 Ministère de la Santé Publique, Niamey, Niger; 31 University of Dar es Salaam, Dar es Salaam, Tanzania, United Republic of; 32 Université Joseph Ki-Zerbo, Ouagadougou, Burkina Faso; 33 University of Zambia School of Public Health, Lusaka, Zambia; 34 Institute of Child Health, College of Medicine, University of Ibadan, Ibadan, Oyo State, Nigeria; 35 Direction de l’Informatique et de l’Information Sanitaire, Abidjan, Côte d'Ivoire; 36 University of Liberia, Monrovia, Montserrado, Liberia; 37 Bagamoyo Research and Training Centre, Ifakara Health Institute, Bagamayo, Tanzania, United Republic of; 38 Muhimbili University of Health and Allied Sciences, Dar es Salaam, Tanzania, United Republic of; 39 Ministry of Health Burkina Faso, Ouagadougou, Burkina Faso; 40 Clinical Services Directorate, Ethiopia Ministry of Health, Addis Ababa, Lideta, Ethiopia

**Keywords:** COVID-19, Maternal health, Health systems, Child health, Immunisation

## Abstract

**Introduction:**

There are concerns about the impact of the COVID-19 pandemic on the continuation of essential health services in sub-Saharan Africa. Through the Countdown to 2030 for Women’s, Children’s and Adolescents’ Health country collaborations, analysts from country and global public health institutions and ministries of health assessed the trends in selected services for maternal, newborn and child health, general service utilisation.

**Methods:**

Monthly routine health facility data by district for the period 2017–2020 were compiled by 12 country teams and adjusted after extensive quality assessments. Mixed effects linear regressions were used to estimate the size of any change in service utilisation for each month from March to December 2020 and for the whole COVID-19 period in 2020.

**Results:**

The completeness of reporting of health facilities was high in 2020 (median of 12 countries, 96% national and 91% of districts ≥90%), higher than in the preceding years and extreme outliers were few. The country median reduction in utilisation of nine health services for the whole period March–December 2020 was 3.9% (range: −8.2 to 2.4). The greatest reductions were observed for inpatient admissions (median=−17.0%) and outpatient admissions (median=−7.1%), while antenatal, delivery care and immunisation services generally had smaller reductions (median from −2% to −6%). Eastern African countries had greater reductions than those in West Africa, and rural districts were slightly more affected than urban districts. The greatest drop in services was observed for March–June 2020 for general services, when the response was strongest as measured by a stringency index.

**Conclusion:**

The district health facility reports provide a solid basis for trend assessment after extensive data quality assessment and adjustment. Even the modest negative impact on service utilisation observed in most countries will require major efforts, supported by the international partners, to maintain progress towards the SDG health targets by 2030.

**WHAT IS ALREADY KNOWN ON THIS TOPIC**

- The COVID-19 pandemic affected countries in Africa, causing increased number of cases and deaths and the implementation of restrictions and lockdown measures.
- Multiple modelling studies and other studies have raised major concerns about the impact of the pandemic and the associated service disruptions on health outcomes, particularly for women and children in sub-Saharan Africa where mortality levels are highest.
- Working directly with country government institutions and researchers to quantifying the indirect impact of the pandemic on women’s and children’s health and its subnational level variations using at district level time series data from routine health information system will provide more actionable evidence that helps mitigate the effects of the indirect pandemic on vulnerable populations and plan appropriately for future similar pandemics.

**WHAT THIS STUDY ADDS**

- The COVID-19 pandemic caused modest reductions in the utilisation of maternal, newborn and child health services in the order of 2%–6%, and larger reductions in outpatient consultations and hospitalisations (7%–17%) during March to December 2020, with months immediately following the start of the pandemic showing larger reductions.
- Large variations in service utilisation across subnational areas were observed, with rural areas more affected than urban areas; Eastern African countries were more impacted than West African countries.
- Stronger government response, at the beginning of the pandemic, was associated with larger reduction in general service utilisation but had limited effects on maternal, newborn and child health services.

**HOW THIS STUDY MIGHT AFFECT RESEARCH, PRACTICE AND/OR POLICY**

- District routine health information systems offer unique opportunities to measure the size of and disparities in indirect negative impact of the pandemic on maternal, newborn and child health services, and it is possible to work collaboratively with countries to assess the impact on the continuation of services.
- It will take a major effort of countries in terms of policies and programmes, supported by the international partners, to overcome the adverse effects of the pandemic on the progress towards the ambitious Sustainable Development Goals targets for health by 2030.

## Introduction

The COVID-19 pandemic may affect the utilisation of health services through disruptions in the provision of routine health services as well as changes in the demand for services. Multiple modelling studies in a range of health fields have raised major concerns about the impact of the pandemic and the associated service disruptions on health outcomes, particularly for women and children in sub-Saharan Africa where mortality levels are highest.^1–3^ Systematic reviews have shown impact on maternal and perinatal outcomes such as maternal deaths, stillbirths and pregnancy complications, even though the number of studies are still limited and biases are probable.^4 5^

Data on the continuation of essential health services are critical to assess and guide responses to mitigate the indirect consequences of the pandemic. The WHO has conducted multiple rounds of key informant surveys to assess the impact of the pandemic on the continuation of health services in countries. The results suggest that important disruptions occurred. During the first survey round in May-July 2020, participating countries in WHO Africa region reported at least a partial decrease in services, defined as a decline of 5% or more from usual levels, for 60% of the essential health services (median), with outpatient services as the most affected.^6^ In the second round of the key informant survey, referring to the period January-March 2021, this proportion had fallen to 45% among 40 participating countries in the WHO Africa region.^7^ There is however a need for more exact data on the impact on the continuation of services.

In general, routine health facility reports are the main data source for the continuous monitoring of service utilisation at local and national levels. This is even more so the case during a pandemic when population-based data collection is halted due to restrictions on travel and face-to-face human interactions. Several studies based on health facility data have registered adverse consequences on the use of specific services in multiple countries in the first months following the declaration of a global pandemic on 11 March 2020. The results mostly showed an adverse trend in the utilisation of health services but with considerable variability between countries, type of services and time periods during 2020.^8–12^

Since 2018, the Countdown to for Women’s, Children’s and Adolescents’ Health, an international collaboration of academics and global agencies, and the African Population and Health Research Center (APHRC), the Countdown’s lead institution for Africa, have been working with country public health institutions and ministries of health in sub-Saharan Africa on strengthening the analyses of monthly data reported by health facilities, also referred to as routine health information system, to inform countries’ own reviews of progress and performance.^13 14^ This work has shown the strengths and weaknesses of routine health information systems, contributed to the development of systematic approaches to data quality assessment and adjustment, and laid the foundation for this assessment of the impact of the pandemic on health service utilisation.^15–19^

The current study builds on this Countdown work in sub-Saharan Africa, aiming to expand the knowledge on the disruptions and rebounds in health services utilisation during March to December 2020 for a selected healthcare services, including maternal and newborn care, immunisation, family planning and general services (outpatient visits and admissions). We assessed national, urban and rural monthly trends in West and Eastern African countries, as well as the extent to which stringency of measures in response to the threat of COVID-19 and reported cases were associated with the changes in service utilisation.

## Methods

**Figure 1:**
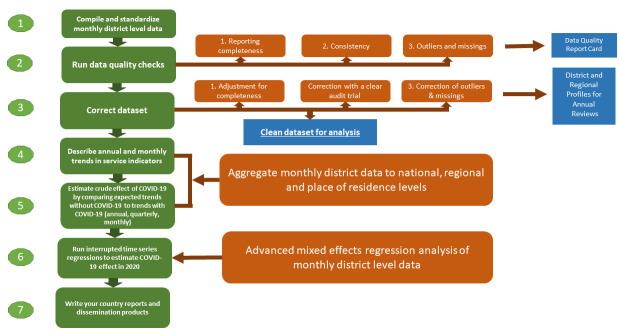
Stepwise analysis process with country teams.

The study was conducted as part of the existing multi-year Countdown country collaborations which include analysts from public health institutions and the ministry of health. Among the 13 countries in sub-Saharan Africa that currently have Countdown collaborations, 12 countries expressed an interest in a joint study: Burkina Faso, Cote d’Ivoire, Ghana, Liberia, Mali, Niger and Nigeria in West Africa and Ethiopia, Kenya, Tanzania, Uganda and Zambia in Eastern Africa. Eight web-based workshops were organised during February to July 2021 to jointly go through a stepwise analytical process of data compilation, data quality assessment and adjustment, analysis, and interpretation of results (figure 1). All workshops were supported by a suite of analytical codes developed in Stata V.15.0.^20^

The main data source is the routine health information system. Data are reported by health facilities to district offices on a monthly basis using standardised reporting forms which differ between countries. In general, district offices enter the data into computers using the most recent version of the District Health Information System software (also known as DHIS2).^21^ Initial data quality issues are identified and corrected at district level. The district files are compiled at the national level (and sometimes at regional/provincial level) and checked for completeness and quality.

Facility data have a range of quality issues related to completeness, extreme outliers and internal consistency over time and between interventions which were addressed using a set of methods developed by WHO^22^ and Countdown to.^15^ Data were extracted from DHIS2 by the country teams and put into standardised Excel sheets. Monthly totals by district for the period January 2017 to December 2020 were compiled for the following interventions: outpatient visits (total), inpatient admissions (IPD), antenatal care visits (one and four or more), institutional deliveries (or skilled birth attendance if this was not available in the country system), caesarean sections, infant vaccinations (one and three doses of pentavalent vaccinations, measles, BCG). Countries also compiled additional data on monthly facility reporting completeness rate, total population, region, type of district (urban–rural–mixed) and government preventive public health measures to control the spread of the pandemic. All countries focused on the district data, except Ethiopia and Nigeria which extracted regional and state data, respectively, and Kenya which used counties. Districts were classified into urban, rural and mixed based on countries’ definition. In Ethiopia and Uganda, the urban–rural comparison was limited to capital city region or districts compared with the rest of the country.

The steps of the joint analyses with the country teams are summarised in figure 1. The analytical approach started with the assessment of data quality and preparation of a curated data sets by adjusting for incomplete reporting by health facilities and correcting missing values and improbably extreme outlying values in the reported monthly data from each district.

The assessment and adjustments were made using common rules. Data quality checks were performed with district, regional and national level data. The districts with problematic reporting rates and inconsistencies were flagged for further examination and, if needed, corrections.

Completeness of reporting by health facilities (the proportion of facilities that reported data for a given month out of all facilities expected to report data) affects the number of reported events and was assessed at the district level. Each country summarised the per cent of district-months with facility reporting rates below 90% and listed all district-months with facility reporting rates below 75%. In the latter case, the median value of the calendar year was imputed for the month.

For all other districts we adjusted for incomplete reporting by considering the completeness of reporting by facilities and the level of service provision expected from non-reporting facilities. For the latter, we use an adjustment factor ranging from 0 to 1, where 1 means similar level of services as reporting facilities and 0 assumes that non-reporting facilities provided no services.^23^ Decision on this adjustment factor is guided by knowledge of service provision and distribution of facilities in each country. In most countries, the default adjustment factor of 0.25 was selected after discussion among the country team on the most appropriate factor for each intervention. An example of different adjustment factors was Burkina Faso which used 0.25 for preventive services (ANC, vaccination, FP), 0.5 for delivery, outpatient consultation (OPD) and IPD, and assumed no services provided in districts located in insecure areas. In case of missing values, the median value of the calendar year was used, unless there was reason to believe that it was a true zero.

Extreme outliers were identified using a modified Z-score which is a standardised score of observations measuring the deviation from the median, obtained by dividing the difference from the median by the median absolute deviation. Monthly data with a score greater than 5 SD from the annual median were identified as extreme outliers.^24 25^ These were corrected by imputing a value based on the median value of the calendar year.

The national summary was created bottom-up from the cleaned and adjusted district data. Similarly, we created summaries by region and by urban–rural–mixed type of districts. A summary of data quality captures both the national values for each year during 2017–2020 and the per cent of districts with good data for each of the three dimensions of data quality (table 1).

**Table 1:** Number of subnational units, cumulative number of reported COVID-19 cases per 100 000 population in 2020, and mean containment stringency index during March–December 2020

Country	Type of subnational units (eg, districts, regions, provinces, etc)	No of subnational units used for the analyses	Cumulative no of reported COVID-19 cases per 100 000 pop	Mean, containment stringency index
West Africa				
Burkina Faso	Districts	70	31.2	43.6
Cote d'Ivoire	Districts	113	83.1	44.6
Ghana	Districts	260	172.6	48.5
Liberia	Counties	15	34.7	65.1
Mali	Districts	75	34.0	54.3
Niger	Districts	72	13.2	32.4
Nigeria	States	37	41.4	63.1
Average			58.6	50.2
Eastern Africa				
Ethiopia	Regions	12	105.4	65.9
Kenya	Counties	47	175.4	66.2
Tanzania	District councils	184	0.8*	
Uganda	Districts	136	74.7	74.9
Zambia	Districts	116	109.5	46.1
Average†			116.3	63.3

*Tanzania did not report cases from May 2020.

†Excludes Tanzania.

Using the final curated data sets, each country team compared the observed monthly service utilisation patterns, obtained from the aggregate of all districts with an expected level of service use based on data from the previous years, starting January 2017. In the final models for^23^ Kenya and Liberia, the year 2017 was excluded as these data were affected by national strikes which distorted the trend analysis to obtain the expected values. The period April–October 2019 for Burkina Faso was excluded for the same reason. For Ethiopia, only monthly data from 2019 to September 2020 were used, as the transition into the DHIS2 platform occurred in 2017–2018 which affected the quality of data. The country teams performed descriptive analysis by assessing annual, quarterly and monthly trends in service utilisation to visualise any changes in the utilisation of specific services.

The final analysis relied on mixed effects ordinary least squared regression models to estimate the size of any change in service utilisation for each COVID-19 month from March to December 2020 and for the whole COVID-19 period in 2020. We fitted these regressions separately for each country using the monthly number of service utilisation at district level as the dependent variable (equation 1). We used districts as units of analysis to reduce the noise in monthly data reported by each facility and to allow a more accurate assessment of the disruption to service utilisation by accounting for within district shifts in service utilisation between facilities. This dependent variable (Y_ij_) was regressed on a time variable (time) defined in months (from January 2017 to December 2020) to capture time trends, calendar month (month) to control of seasonality in service use, district population (pop), type of district (area=urban, rural or mixed), first administrative level (region), and COVID-19 month from March 2020 to December 2020 (covid month). Equation (1) presents the model. The coefficient of each COVID-19 month dummy variable expresses the average change in service utilisation in the particular month. To obtain the average change in service utilisation over the entire COVID-19 period, we fitted another model with a dummy variable for this period. The mixed effects models included random intercept and slope for time trends, accounting for multiple measurements at the district levels. We computed the average per cent change in services utilisation during COVID-19 months along 95% CIs at national and area (urban, rural, mixed) levels. We compared these measures to those obtained by aggregating the predicted monthly change of service utilisation across districts and found them to be generally similar.

(1) $$Y_{ij}=\beta_{0j}+\beta_{1j}time\sum_{m=2}^{12}\beta_{m}Month_{m}+\beta_{13}Pop\;+\sum_{a=14}^{15}\beta_{a}Area+\sum_{r=16}^{n}\beta_{r}Region+\sum_{t=1}^{9}\beta_{ct}Covidmonth+\epsilon_{ij}$$

Using mixed effect linear regressions, we assessed the association between the per cent change in service utilisation for each of the services analysed and COVID-19-related indicators such as the monthly COVID-19 cases and the government response stringency index of COVID-19 restriction measures imposed by each country. The latter indicators were downloaded from Our World in Data.^26^ The stringency index is composite measure based on nine response indicators including school closures, workplace closures and travel bans, rescaled to a value from 0 to 100 (100=strictest response).^27^ Data were extracted from https://github.com/owid/covid-data/tree/master/public/data. The analysis pooled data from all 11 countries and specific services and controlled for population density and total population.

### Patient and public involvement statement

Patients or the public were not involved in the design, implementation or dissemination of this study.

## Results

Table 1 shows the participating countries with the number of subnational units for which monthly health facility data were analysed, with the cumulative number of reported COVID-19 cases per 100 000 population during 2020 and the containment stringency index averaged over the period March–December 2020. Cumulative reported cases of COVID-19 during 2020 were relatively low in all countries, in part due to lack of testing, with four countries having rates just over 100 per 1 000 000 population (Kenya, Ghana, Zambia and Ethiopia) and most west African countries having rates below 50. The government response was strongest in Uganda, followed by Kenya, Ethiopia and Nigeria, while Tanzania and Niger had the lowest scores on the containment stringency index during 2020. On average, Eastern African countries included in the analysis reported higher cumulative cases per 100 000 population and enacted stronger stringency measures than West African countries (table 1).

### Data quality

Table 2 summarises the completeness of monthly health facility reports for the main forms related to antenatal and delivery care, immunisation and outpatient visits. National completeness was over 90% in most countries during 2017–2020 and there was no major decline in monthly reporting during 2020. Nigeria is an exception with considerably lower reporting rates than the other countries.

**Table 2:** Completeness of monthly health facility reports at the national level and per cent of districts with health facility reporting completeness exceeding 90%, 2017–2020

	Completeness of monthly facility reporting (%)				Districts with completeness >90% (%)			
	2017	2018	2019	2020	2017	2018	2019	2020
West Africa								
Burkina Faso	97	98	–	93	90	93	–	79
Cote d'Ivoire	90	94	97	98	59	88	96	98
Liberia	–	95	95	96	–	90	88	92
Ghana	93	94	95	98	77	80	87	96
Mali	92	95	99	99	85	90	99	97
Niger	89	92	87	84	69	73	68	53
Nigeria	74	79	79	68	19	27	27	16
Eastern Africa								
Ethiopia	–	–	84	81	–	–	94	94
Kenya	82	92	94	95		75	79	89
Tanzania	96	97	97	98	87	92	95	98
Uganda	88	89	89	84	59	61	62	74
Zambia	95	96	93	96	100	98	75	87
Country median (all)	91	94	94	96	73	88	87	91

Note: data excluded due to completeness issues caused by national strikes (Burkina Faso, Kenya and Liberia) or transition to DHIS-2 (Ethiopia).

DHIS, District Health Information System.

The per cent of districts with facility reporting completeness exceeding 90% was more variable with four countries exceeding 95% in 2020. In general, reporting completeness in 2020 was as high or even better than in previous years. In some years, less than two-thirds of the districts had reporting rates over 90%, including all years in Nigeria, and 3 of the 4 years in Uganda. There was no evidence of a major decline in completeness in 2020. The low reporting rate in Kenya in 2017 was related to national health worker strike and is excluded from the trend analysis. A similar issue related to a strike led to the exclusion of 2017 data for Liberia and 2019 for Burkina Faso.

Missing monthly values were rare, except in 2020 in Nigeria (41% of expected reports, see online supplemental appendix 1). Extreme outliers were also uncommon (less than 3% of monthly values each year), in Mali (2020, 4%) and Nigeria (2020, 4%). These extreme outliers were inspected and corrected as described in the methods section. Country details on reporting completeness, missing values and extreme outliers are shown in online supplemental appendix 1. Nigeria was excluded from this analysis of service utilisation trends because of the low and variable completeness of reporting which would render its trends highly unreliable.

10.1136/bmjgh-2021-008069.supp1 Supplementary data

### Continuation of essential health services

**Figure 2:**
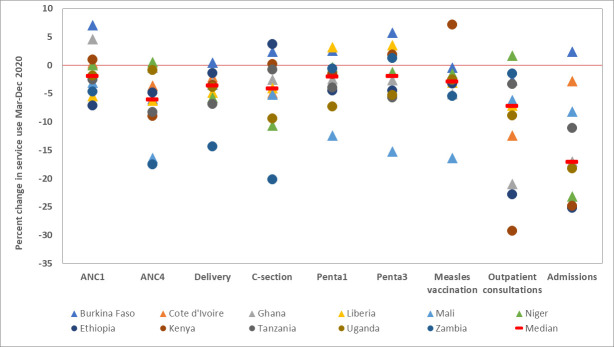
Per cent change in utilisation of selected health services during March–December 2020, compared to expected utilisation based on the preceding 3 years.

Table 3 and figure 2 summarise the per cent change in utilisation of nine health services for the whole period March–December 2020, compared with the expected utilisation derived from the regression analysis with monthly data for 2017–2019. The median change of the nine services was negative for all countries, except Burkina Faso. The median reduction was small in Niger and Kenya (≤2%), ranged from 2% to 5% in all other countries except Mali where the median reduction was 8%.

**Table 3:** Per cent change in utilisation of selected health services during March–December 2020, compared with expected utilisation based on the preceding 3 years

Country	ANC-1		ANC-4		Delivery		C-section		Penta-1		Penta-3		Measles vaccination		OPD		Inpatient Admissions		Median
West Africa	%	95% CI	%	95% CI	%	95% CI	%	95% CI	%	95% CI	%	95% CI	%	95% CI	%	95% CI	%	95% CI	
Burkina Faso	7.1	(4 to 10)	−6.0	(−9 to −3)	0.5	(−2 to 3)	2.4	(−11 to 16)	2.6	(0 to 6)	5.8	(3 to 9)	−0.4	(−4 to 3)	−2.6	(−6 to 1)	2.4	(−3 to 8)	2.4
Cote d'Ivoire	0.4	(−2 to 3)	−3.6	(−6 to −2)	−2.1	(−4 to 0)	−4.9	(−10 to 0)							−12.4	(−14 to −10)	−2.8	(−7 to 2)	−3.2
Ghana	4.6	(3 to 6)	−0.4	(−2 to 1)	−3.1	(−4 to −2)	−2.5	(−5 to 0)	−2.4	(−4 to −1)	−2.6	(−4 to −1)	−5.1	(−6 to −4)	−21.0	(−23 to −19)	−17	(−20 to −14)	−2.6
Liberia	−5.4	(−12 to 1)	−6.2	(−13 to 1)	−4.8	(−9 to −1)	−4.0	(−16 to 8)	3.2	(−3 to 9)	3.6	(−3 to 10)	−3.0	(−10 to 4)	−7.1	(−13 to −1)			−4.4
Mali	−3.0	(−5 to −1)	−16.4	(−20 to −13)	−3.4	(−6 to −1)	−5.1	(−10 to −1)	−12.4	(−14 to −10)	−15.2	(−17 to −13)	−16.4	(−19 to −14)	−6.2	(−10 to −3)	−8.2	(−18 to 2)	−8.2
Niger	0	(−2 to 2)	0.5	(−2 to 3)	−5.7	(−8 to −3)	−10.6	(−26 to 4)	−0.3	(−2 to 1)	−1.2	(−3 to 1)	−1.4	(−3 to 1)	1.7	(−3 to 6)	−23.2	(−40 to −6)	−1.2
Median (West Africa)	0.2		−4.8		−3.2		−4.0		−0.3		−1.2		−3.0		−6.6		−12.6		
Eastern Africa																			
Ethiopia	−7.1	(−14 to 0)	−4.8	(−10 to 0)	−1.4	(−5 to 2)	3.8	(−3 to 11)	−4.5	(−9 to 0)	−4.4	(−9 to 0)	−3.2	(−11 to 4)	−22.8	(−30 to −16)	−25.1	(−35 to −16)	−4.5
Kenya	1.0	(−1 to 3)	−8.9	(−12 to −6)	−3.5	(−5 to −2)	0.2	(−2 to 3)	−1.5	(−3 to 0)	1.9	(0 to 4)	7.2	(4 to 10)	−29.2	(−32 to −27)	−24.8	(−28 to −22)	−1.5
Tanzania	−2.6	(−3 to −2)	−8.2	(−10 to −7)	−6.8	(−8 to −6)	−0.8	(−3 to 1)	−3.9	(−5 to −3)	−5.7	(−7 to −4)	−2.6	(−4 to −1)	−3.3	(−5 to −1)	−11.1	(−13 to −9)	−3.9
Uganda	−1.8	(−3 to 0)	−0.8	(−3 to 1)	−3.8	(−6 to −2)	−9.4	(−15 to −4)	−7.2	(−9 to −6)	−5.3	(−7 to −4)	−2.5	(−6 to 1)	−8.9	(−18 to 0)	−18.2	(−21 to −15)	−5.3
Zambia	−4.7	(−7 to −3)	−17.5	(−20 to −15)	−14.3	(−17 to −12)	−20.2	(−29 to −11)	−0.6	(−3 to 1)	1.3	(−1 to 3)	−5.4	(−8 to −3)	−1.5	(−4 to 1)			−5.0
Median (Eastern Africa)	−2.6		−8.2		−3.8		−0.8		−3.9		−4.4		−2.6		−8.9		−21.5		
Median	−1.8		−6.0		−3.5		−4.0		−2.0		−1.9		−2.8		−7.1		−17.0		−3.9

*Empty cells denote data issues that prevented accurate computation of the indicator.

ANC, antenatal care; C-section, Caesarian section; OPD, Outpatient consultations; Penta, Pentavalent vaccination.

There was, however, major variability by service. The greatest reduction in service use occurred for hospital admissions, driven by major reductions (over 10% of expected numbers) in most countries except Burkina Faso, Mali and Cote d’Ivoire. The second most affected service was outpatient visits, driven by Kenya, Ethiopia, Ghana and Cote d’Ivoire. The median change in services was generally higher in Eastern Africa than in West Africa. The changes in the continuation of services for antenatal care (first visit), institutional deliveries and C-sections were negative in eastern African countries, with Zambia as the most affected country. The impact on immunisation services appeared modest in most countries, with the exception of Mali.

**Figure 3:**
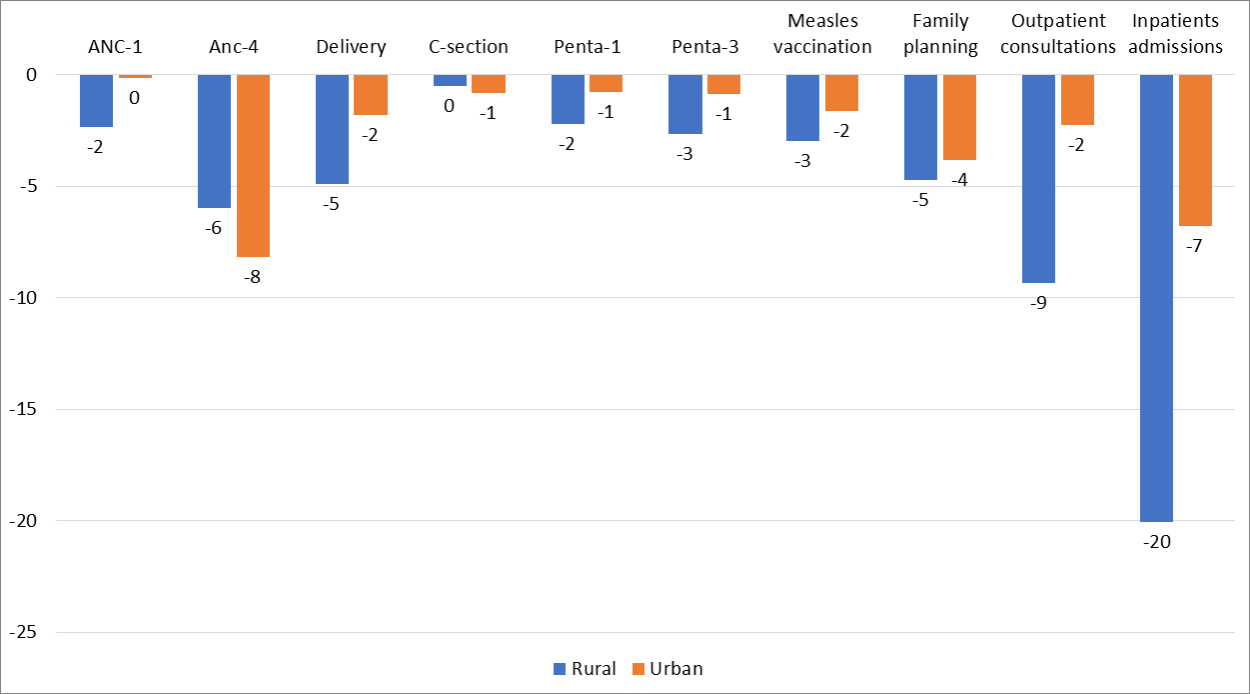
Median change in service utilisation across countries during March–December 2020 by type of service and urban–rural district group. Mixed areas not included. Only five countries reported mixed areas. ANC, antenatal care; C-section, Caesarian section; Penta, Pentavalent vaccination.

We also examined the results by region of each country. Large variations in the changes in service utilisations were observed across regions (see online supplemental appendix 2 for estimates of changes in hospital admissions). However, capital cities where the COVID-19 restrictions cases were expected to have been most pronounced, were not the places with highest disruption in service utilisation. In addition, there was an indication of higher disruption in subnational areas affected by ongoing security issues, especially in Mali and Niger. In Mali, the drop in services was much larger in the three regions most affected by security issues in the north (Taoudénit, Ménaka et Kidal). In Niger, Agadez, Diffa and Tillaberi were the most affected. In general, we could expect the spread of COVID-19 to be greater in urban than rural districts in the initial stages of the pandemic, and perhaps also the containment measures to be earlier. However, a comparison of urban, rural districts showed that the median drops in service utilisation across countries was consistently higher in rural areas than urban, regardless of the type of service (figure 3).

### COVID-19 stringency measures and monthly changes in service utilisation

**Figure 4:**
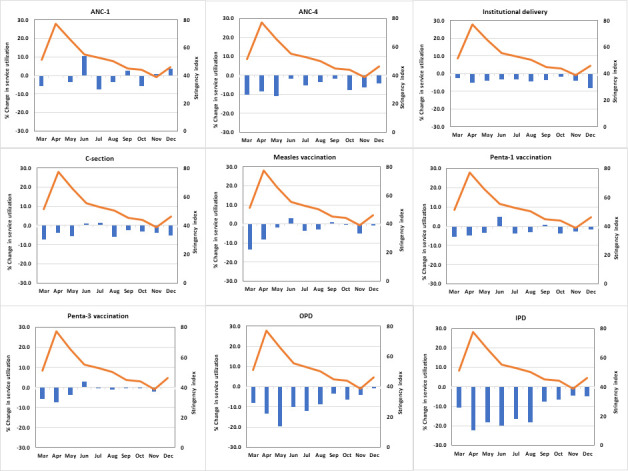
Median stringency index for COVID-19 restrictions and median changes (%) in service utilisation during March–December 2020, by type of service. IPD, inpatient admissions; OPD, outpatient consultations.

We further examined the changes in service utilisation during 2020 by month to document variation over time including declines and rebounds and ascertain the association with the reported case load and the response in greater detail. Figure 4 shows the trends in monthly median change by service for all countries combined. Online supplemental appendix 2 presents the country specific results. Overall OPD and inpatients admissions were the most disrupted services. These two services show the largest drop in the first 3–4 months following the lockdown (March–June) reaching 20%–25% reduction. Median utilisation reductions decreased gradually thereafter but IPD did not reach the expected level by December while the drop faded completely for OPD. For immunisation services, there was a noticeable drop in the 2 months following the lockdown, followed by a rebound to the expected levels in most countries.

Figure 4 also overlays the trend in average monthly stringency measure on top of the monthly per cent change in service utilisation. Overall, countries enacted COVID-19 restriction measures in March. These measures grew stronger in the following 2 months before gradually diminishing. The drop in service utilisation did not follow a similar pattern except for OPD and IPD, and, to a lesser extent, immunisation services.

Table 4 shows the coefficients from mixed effects regression to assess the per cent change in service associated with the response stringency index, while controlling for the number of new monthly COVID-19 cases, population size and density.

**Table 4:** Change in service utilisation associated with COVID-19 cases, stringency index and population measures

Variables	Change in service utilisation								
	ANC-1	ANC-4	Delivery	C-section	Measles vaccination	Penta-1 vaccination	Penta-3 vaccination	Inpatient admissions	Outpatient consultations
No of new monthly COVID-19 cases (in 1000s)	0.1975	0.148	0.011	−0.217	0.196	0.134	0.180	−0.095	−0.186
Stringency index (%)	−0.080*	−0.083*	−0.018	−0.027	−0.059	−0.039	−0.048	−0.363***	−0.307***
Population (in 100000)	−0.008*	−0.002	0.001	0.012	0.001	−0.004	−0.006	−0.010	−0.012*
Population density	0.033	0.071*	0.024	0.011	0.029	−0.003	0.013	0.047	−0.002

**P<0.05, ***p<0.001.

ANC, antenatal care; C-section, Caesarian section; OPD, Outpatient consultations; Penta, Pentavalent vaccination.

The stringency index was significantly associated with reductions in OPD and IPD in similar proportions. On average, a 10 percentage points increase in the stringency index was associated with a 3.1–3.6 percentage points reduction in these services. No significant association was observed with changes in the utilisation of other types of services, except for ANC-1 and ANC-4 where there was a marginally significant decline of 0.8% for every 10 percentage points increase in the stringency index.

## Discussion

The analysis of routine health facility data in 12 countries in sub-Saharan Africa resulted in several key findings on the impact of the COVID-19 pandemic on the continuation of essential health services. First, the overall impact on services utilisation during March–December 2020 was negative. The impact on the utilisation of maternal and child health preventive interventions was of the order of −2% to −6%, while general outpatient services and especially IPD dropped by as much as 18%. Zambia stood out at the only country with substantial impact maternal health services. Our findings are generally consistent with those of Ahmed and Roberton who conducted their study in eighteen countries, with six countries overlapping with our study.^28^ Even though the decline in maternal, newborn and child health (MNCH) preventive services could be considered modest in proportion to multiple global concerns, it does represent a significant stall and even a reverse in the progress towards universal coverage of essential health interventions for MNCH. Increases in coverage of interventions during the past two decades were typically 1%–2% percentage points per year.^29 30^ Therefore, the interruption of this increase in 2020 decline can be considered a setback in progress towards the 2030 Sustainable Development Goals (SDG) targets by as much as 2–3 years.

The reduction in admissions to hospitals was largest in every country, even though there is little evidence of a major increase in COVID-19-related hospitals admissions in 2020. A change in admission policies may have been an important factor, although we have no concrete evidence from the 12 countries. The concomitant decrease in outpatient utilisation suggests that a decline in demand for services from the patient side also played a role. Factors such as fear of infection and disruptions of the transport systems have been found to be critical factors in other settings.^31–34^

Second, in the analysis of the pooled data set, a stronger government response was associated with significantly greater drops in OPD and IPD, but not in maternal, newborn and child health service utilisation. This was not related to closing of health facilities, as facility reporting rates were excellent in 2020, but possibly due to changed practices such as admission criteria and opening hours, as well as patient factors.^35^ In addition, there is some evidence that effective measures were put in place to mitigate the impact on maternal service utilisation.^36^

Third, the monthly data showed that the impact on service utilisation was greatest in the months immediately following the declaration of a pandemic, even though cases were still few. The response was forceful in many countries, according to the response stringency index. The restriction measures may have exacerbated fears of contamination, misconceptions about the transmission modes and distrust in the quality of health services, and treatment offered in case of COVID-19 diagnosis.^37^ In most countries, there was evidence of a rebound in service utilisation during the second half of 2020, but this did not suffice to erase the losses in the early months.

Fourth, even though the assessment of the impact of COVID-19 was our primary purpose, many other factors may influence the trends in service utilisation. This was most obvious in countries affected by disruptions such as armed conflict (Mali and Niger) or health worker strikes (Burkina Faso, Kenya and Liberia). We cannot exclude that a slowdown of service utilisation irrespective of the pandemic has contributed to our findings. The expected numbers are based on the trend in preceding 3 years. Many countries are moving into higher coverage territory where it is harder to maintain the growth rate seen in previous years.

Fifth, there was, perhaps unexpectedly, slightly greater impact on service utilisation in rural than in urban districts across all countries and for virtually all service indicators. It is likely that COVID-19 transmission was greater in urban areas, and perhaps government responses were also stricter (although no such data were available), but this did not result in greater impact in urban areas.11,^32^ It is also possible that there were shifts in service utilisation across facilities within urban areas compared with rural areas where formal careseeking options are more limited. Rural facilities may have faced more closures, stock-outs and reduced availability of health personnel than urban areas. There may have also been higher COVID-19-related stigma in rural than in urban areas. Household level interviews with women will help understand changes in careseeking patterns across these areas that are associated with the pandemic. There were also large variations in impact on service utilisation across countries within and between regions. However, service utilisation in Eastern African countries appeared to have been more affected that in West African countries. Reasons for such differences may reside in the reported size of the pandemic and the level of stringency measures that were also higher in Eastern African countries.

Health facility reports are known to have multiple data quality issues.^14^ There is evidence of improvements in many countries, but it is not surprising that data recorded, compiled and reported by health workers in thousands of clinics and compiled in hundreds of district offices are prone to errors. Our systematic approach aimed to address data issues and the high reporting completeness and manageable number of outliers and missing values, summarised with data quality measures, as well as the consistency of findings across countries, give good confidence in the results.

The data and analysis used have some limitations. The routine facility data compiled at district level in our analysis, after adjustment for completeness, identification of outliers and missing values and their cleaning. Using facility-level data may add value, but our results were consistent with such approaches and have the advantage of alignment with the way routine health information systems generally operate to synthesise data and results. Although our assumptions were conservative, they may have affected slightly our findings. However, given the assumptions were independent of the COVID-19 measures and the adjustments to the data were done mostly prior to the start of the pandemic, we expect these effects to be minimal. The modelling analysis used data from the past 3 years prior to the pandemic to predict service utilisation levels during the first year of the pandemic and resulted in large confidence intervals. A longer time period, both prior and during the pandemic, might have provided more stable and efficient estimates of the effects of the pandemic. Data on COVID-19 monthly new cases and the government stringency measures were obtained from the OurWorldInData portal, a compilation of COVID-19 data from all countries. Given limited testing capacity and uptake in countries included in our analysis, the number of reported cases is probably severely underestimated and increased as a results of testing capacity. Similarly, inaccuracy in measures that compose the stringency index could affect our findings.

The Countdown to 2030 focuses on taking some of the principles of survey analysis to routine health facility data analysis. Well-intended immediate production of numbers and coverage statistics from facility data may be useful for local score cards, but data quality problems must be identified and addressed. This should lead to a curated data file, similar to survey data procedures, before diving into analysis. Our study shows that such cleaned data sets are useful for analysis of trends in reported events. But they can also be used for population service utilisation and intervention coverage analyses for multiple indicators, provided the issue of the denominator or target population can be approximated with some accuracy.^23^

A fundamental element of epidemic preparedness is that countries have the capacity to conduct this kind of analysis of indirect impact on the continuation of services using facility data during any crisis. This is critical for an effective response. The collaborative research process described in our paper, led by APHRC and the Countdown to 2030, present a sincere attempt to strengthen country analytical capacity in countries through longer-term collaborations focused on informing country-led review processes, minimising the dependence on external mechanisms that extract country data and often pay only lip service to country engagement.

In summary, it is possible by working with countries and with routine health information system data to assess the impact of COVID-19 on the continuation of MNCH and general health services. To some extent, the results confirm the reports by key informants in the WHO surveys in 2020 and early 2021, with modest but variable impact in most countries especially in the early months and then reducing over time without full recovery. It will take a major effort of countries, supported by the international partners, to overcome the negative effects of the pandemic on the progress towards the SDG health targets by 2030 and get back on track for the ambitious SDG targets.

10.1136/bmjgh-2021-008069.supp2 Supplementary data

## Data Availability

Data are available on reasonable request. Data analysed in this study was obtained from DHIS-2 from countries that participated in the study. Readers interested in replicating the analysis may contact countries for access to the datasets, on reasonable request. The corresponding authors will provide connection to the country teams for access the data.
