# Supplementary material for: Health service utilisation during the COVID-19 pandemic in sub-Saharan Africa in 2020: a multicountry empirical assessment with a focus on maternal, newborn and child health services
Source: BMJ Glob Health. 2022 May 2;7(5):e008069. doi: 10.1136/bmjgh-2021-008069 (PMC9062456; doi:10.1136/bmjgh-2021-008069)
Supplement: Supplementary data [file bmjgh-2021-008069supp001.pdf]

Appendix -1

Summary of DHIS2 data quality 2017-2020 using tracer indicators (ANC, delivery, vaccination and OPD)

Country	Data quality metric	2017	2018	2019	2020
Burkina Faso	% of expected monthly facility reports (mean, national) *	97	98	81	93
Burkina Faso	% of districts with completeness of facility reporting >= 90% *	90	93	53	79
Burkina Faso	% of districts with no missing monthly values in the year *	100	100	100	100
Burkina Faso	% of monthly values that are not extreme outliers (mean, national) *	100	100	78	100
Burkina Faso	% of districts with no extreme outliers in the year *	100	100	46	95
Burkina Faso	% of districts with ANC1-penta1 ratio between 1.0 and 1.5	63	67	51	60
Burkina Faso	% of districts with penta1-penta3 ratio between 1.0 and 1.5	66	90	66	90
**Burkina Faso**	**Annual data quality score (mean, %)**	**88**	**93**	**68**	**88**
Cote d'Ivoire	% of expected monthly facility reports (mean, national) *	90	94	97	98
Cote d'Ivoire	% of districts with completeness of facility reporting >= 90% *	59	88	96	98
Cote d'Ivoire	% of districts with no missing monthly values in the year *	98	99	99	69
Cote d'Ivoire	% of monthly values that are not extreme outliers (mean, national) *	99	100	99	100
Cote d'Ivoire	% of districts with no extreme outliers in the year *	94	97	97	98
Cote d'Ivoire	% of districts with ANC1-penta1 ratio between 1.0 and 1.5	51	44	36	18
Cote d'Ivoire	% of districts with penta1-penta3 ratio between 1.0 and 1.5	74	79	90	91
**Cote d'Ivoire**	**Annual data quality score (mean, %)**	**81**	**86**	**88**	**82**
Ethiopia	% of expected monthly facility reports (mean, national) *	-	-	84	81
Ethiopia	% of districts with completeness of facility reporting >= 90% *	-	-	94	94
Ethiopia	% of districts with no missing monthly values in the year *	-	-	100	100
Ethiopia	% of monthly values that are not extreme outliers (mean, national) *	-	-	94	94
Ethiopia	% of districts with no extreme outliers in the year *	-	-	92	84
Ethiopia	% of districts with ANC1-penta1 ratio between 1.0 and 1.5	-	-	92	75
Ethiopia	% of districts with penta1-penta3 ratio between 1.0 and 1.5	-	-	100	100
**Ethiopia**	**Annual data quality score (mean, %)**	**-**	**-**	**94**	**90**
Ghana	% of expected monthly facility reports (mean, national) *	93	94	95	98
Ghana	% of districts with completeness of facility reporting >= 90% *	77	80	87	96
Ghana	% of districts with no missing monthly values in the year *	100	100	100	100
Ghana	% of monthly values that are not extreme outliers (mean, national) *	100	100	100	99
Ghana	% of districts with no extreme outliers in the year *	97	97	97	94
Ghana	% of districts with ANC1-penta1 ratio between 1.0 and 1.5	30	27	22	29
Ghana	% of districts with penta1-penta3 ratio between 1.0 and 1.5	47	49	44	46
**Ghana**	**Annual data quality score (mean, %)**	**77**	**78**	**78**	**80**

Amouzou A, *et al. BMJ Global Health* 2022; 7:e008069. doi: 10.1136/bmjgh-2021-008069

Country	Data quality metric	2017	2018	2019	2020
Kenya	% of expected monthly facility reports (mean, national) *	-	92	94	95
Kenya	% of districts with completeness of facility reporting >= 90% *	-	75	79	89
Kenya	% of districts with no missing monthly values in the year *	-	100	100	100
Kenya	% of monthly values that are not extreme outliers (mean, national) *	-	100	100	99
Kenya	% of districts with no extreme outliers in the year *	-	98	98	94
Kenya	% of districts with ANC1-penta1 ratio between 1.0 and 1.5	-	70	70	96
Kenya	% of districts with penta1-penta3 ratio between 1.0 and 1.5	-	91	94	91
**Kenya**	**Annual data quality score (mean, %)**	**-**	**90**	**91**	**95**
Liberia	% of expected monthly facility reports (mean, national) *	-	95	95	96
Liberia	% of districts with completeness of facility reporting >= 90% *	-	90	88	92
Liberia	% of districts with no missing monthly values in the year *	-	100	100	100
Liberia	% of monthly values that are not extreme outliers (mean, national) *	-	100	100	98
Liberia	% of districts with no extreme outliers in the year *	-	98	98	88
Liberia	% of districts with ANC1-penta1 ratio between 1.0 and 1.5	-	27	67	67
Liberia	% of districts with penta1-penta3 ratio between 1.0 and 1.5	-	93	100	100
**Liberia**	**Annual data quality score (mean, %)**	**-**	**86**	**93**	**92**
Mali	% of expected monthly facility reports (mean, national) *	92	95	99	99
Mali	% of districts with completeness of facility reporting >= 90% *	85	90	99	97
Mali	% of districts with no missing monthly values in the year *	96	94	100	98
Mali	% of monthly values that are not extreme outliers (mean, national) *	99	99	98	96
Mali	% of districts with no extreme outliers in the year *	92	93	89	86
Mali	% of districts with ANC1-penta1 ratio between 1.0 and 1.5	30	21	15	27
Mali	% of districts with penta1-penta3 ratio between 1.0 and 1.5	86	84	92	91
**Mali**	**Annual data quality score (mean, %)**	**83**	**82**	**85**	**85**
Niger	% of expected monthly facility reports (mean, national) *	89	92	87	84
Niger	% of districts with completeness of facility reporting >= 90% *	69	73	68	53
Niger	% of districts with no missing monthly values in the year *	100	100	100	100
Niger	% of monthly values that are not extreme outliers (mean, national) *	99	100	99	98
Niger	% of districts with no extreme outliers in the year *	92	96	93	87
Niger	% of districts with ANC1-penta1 ratio between 1.0 and 1.5	19	21	21	31
Niger	% of districts with penta1-penta3 ratio between 1.0 and 1.5	93	93	92	97
**Niger**	**Annual data quality score (mean, %)**	**80**	**82**	**80**	**79**
Nigeria	% of expected monthly facility reports (mean, national) *	74	79	79	68
Nigeria	% of districts with completeness of facility reporting >= 90% *	19	27	27	16

Amouzou A, *et al. BMJ Global Health* 2022; 7:e008069. doi: 10.1136/bmjgh-2021-008069

Country	Data quality metric	2017	2018	2019	2020
Nigeria	% of districts with no missing monthly values in the year *	100	100	100	59
Nigeria	% of monthly values that are not extreme outliers (mean, national) *	99	99	100	96
Nigeria	% of districts with no extreme outliers in the year *	95	89	92	78
Nigeria	% of districts with ANC1-penta1 ratio between 1.0 and 1.5	19	27	22	5
Nigeria	% of districts with penta1-penta3 ratio between 1.0 and 1.5	100	100	97	100
**Nigeria**	**Annual data quality score (mean, %)**	**72**	**74**	**74**	**60**
Tanzania	% of expected monthly facility reports (mean, national) *	96	97	97	98
Tanzania	% of districts with completeness of facility reporting >= 90% *	87	92	95	98
Tanzania	% of districts with no missing monthly values in the year *	100	100	100	100
Tanzania	% of monthly values that are not extreme outliers (mean, national) *	100	100	100	99
Tanzania	% of districts with no extreme outliers in the year *	96	97	96	92
Tanzania	% of districts with ANC1-penta1 ratio between 1.0 and 1.5	88	90	78	76
Tanzania	% of districts with penta1-penta3 ratio between 1.0 and 1.5	89	94	86	89
**Tanzania**	**Annual data quality score (mean, %)**	**94**	**96**	**93**	**93**
Uganda	% of expected monthly facility reports (mean, national) *	88	89	89	84
Uganda	% of districts with completeness of facility reporting >= 90% *	59	61	62	74
Uganda	% of districts with no missing monthly values in the year *	100	100	100	100
Uganda	% of monthly values that are not extreme outliers (mean, national) *	99	100	100	99
Uganda	% of districts with no extreme outliers in the year *	95	97	98	92
Uganda	% of districts with ANC1-penta1 ratio between 1.0 and 1.5	58	68	60	78
Uganda	% of districts with penta1-penta3 ratio between 1.0 and 1.5	94	88	91	88
**Uganda**	**Annual data quality score (mean, %)**	**85**	**86**	**86**	**88**
Zambia	% of expected monthly facility reports (mean, national) *	95	96	93	96
Zambia	% of districts with completeness of facility reporting >= 90% *	100	98	75	87
Zambia	% of districts with no missing monthly values in the year *	100	100	99	100
Zambia	% of monthly values that are not extreme outliers (mean, national) *	100	100	100	99
Zambia	% of districts with no extreme outliers in the year *	99	97	98	94
Zambia	% of districts with ANC1-penta1 ratio between 1.0 and 1.5	78	92	89	91
Zambia	% of districts with penta1-penta3 ratio between 1.0 and 1.5	83	83	87	79
**Zambia**	**Annual data quality score (mean, %)**	**94**	**95**	**92**	**92**

**Mean for ANC, delivery, immunization and OPD services*

APPENDIX - 2

Monthly and overall change in service utilization at national, and by urban-rural-mixed areas

BURKINA FASO

		National			Rural			Urban			Mixed		
Indicator	Month/Period	%change	Lower bound	Upper bound	%change	Lower bound	Upper bound	%change	Lower bound	Upper bound	%change	Lower bound	Upper bound
ANC1	Mar 2020	-12.8	-16.9	-8.7	-18.3	-25.3	-11.3	-9.2	-12.5	-5.9	-11.9	-15.8	-8.0
ANC1	Apr 2020	7.2	2.8	11.6	10.3	3.7	17.0	5.2	1.9	8.4	6.7	2.6	10.8
ANC1	May 2020	6.7	2.7	10.8	9.6	3.5	15.8	4.8	1.8	7.8	6.3	2.5	10.0
ANC1	Jun 2020	22.1	17.3	27.0	31.8	22.2	41.3	15.8	11.6	20.1	20.6	15.9	25.2
ANC1	Jul 2020	1.0	-3.4	5.3	1.4	-4.8	7.6	0.7	-2.4	3.8	0.9	-3.1	4.9
ANC1	Aug 2020	16.6	12.0	21.3	23.9	15.6	32.1	11.9	8.1	15.7	15.5	11.1	19.8
ANC1	Sep 2020	3.1	-1.2	7.5	4.5	-1.8	10.7	2.2	-0.9	5.4	2.9	-1.2	6.9
ANC1	Oct 2020	3.8	-0.5	8.2	5.5	-0.9	11.8	2.7	-0.4	5.9	3.6	-0.5	7.6
ANC1	Nov 2020	16.7	12.5	21.0	24.0	16.1	31.9	12.0	8.4	15.6	15.5	11.5	19.6
ANC1	Dec 2020	7.5	3.5	11.5	10.7	4.5	17.0	5.4	2.4	8.4	7.0	3.2	10.7
**ANC1**	**Total Covid Period**	**6.7**	**4.4**	**9.0**	**9.6**	**5.7**	**13.5**	**4.8**	**3.0**	**6.6**	**6.2**	**4.0**	**8.4**
ANC4	Mar 2020	-22.9	-27.3	-18.5	-30.8	-39.0	-22.7	-17.1	-21.4	-12.9	-21.5	-25.8	-17.2
ANC4	Apr 2020	-9.8	-14.2	-5.3	-13.2	-19.6	-6.7	-7.3	-10.8	-3.8	-9.2	-13.4	-5.0
ANC4	May 2020	-7.7	-11.7	-3.6	-10.3	-16.1	-4.5	-5.7	-8.9	-2.6	-7.2	-11.0	-3.4
ANC4	Jun 2020	-2.0	-6.3	2.4	-2.6	-8.6	3.3	-1.5	-4.7	1.8	-1.8	-6.0	2.3
ANC4	Jul 2020	-5.6	-10.0	-1.2	-7.5	-13.6	-1.4	-4.2	-7.5	-0.8	-5.2	-9.4	-1.1
ANC4	Aug 2020	4.4	0.0	8.8	5.9	-0.1	12.0	3.3	0.0	6.6	4.1	0.0	8.3
ANC4	Sep 2020	10.3	5.8	14.8	13.9	7.3	20.5	7.7	4.1	11.2	9.7	5.4	13.9
ANC4	Oct 2020	1.7	-2.7	6.1	2.3	-3.6	8.3	1.3	-2.0	4.6	1.6	-2.5	5.7
ANC4	Nov 2020	9.2	5.1	13.3	12.4	6.4	18.4	6.9	3.6	10.1	8.7	4.7	12.6
ANC4	Dec 2020	0.4	-3.6	4.4	0.6	-4.9	6.0	0.3	-2.7	3.3	0.4	-3.4	4.2
ANC4	Total Covid Period	-2.9	-5.0	-0.7	-3.9	-6.9	-0.9	-2.2	-3.8	-0.5	-2.7	-4.7	-0.7
Deliveries	Mar 2020	-3.5	-7.9	0.8	-5.3	-11.9	1.3	-2.3	-5.1	0.5	-3.3	-7.4	0.8
Deliveries	Apr 2020	-3.2	-7.9	1.5	-4.8	-11.9	2.3	-2.1	-5.2	1.0	-3.0	-7.4	1.4
Deliveries	May 2020	15.3	10.8	19.9	23.0	14.6	31.4	10.0	6.7	13.2	14.4	10.0	18.7
Deliveries	Jun 2020	1.4	-3.4	6.1	2.0	-5.1	9.1	0.9	-2.2	4.0	1.3	-3.2	5.7
Deliveries	Jul 2020	0.9	-3.9	5.6	1.3	-5.8	8.4	0.6	-2.5	3.6	0.8	-3.6	5.3
Deliveries	Aug 2020	-3.0	-7.7	1.7	-4.5	-11.6	2.6	-2.0	-5.0	1.1	-2.8	-7.3	1.6
Deliveries	Sep 2020	6.5	1.7	11.2	9.7	2.2	17.1	4.2	1.0	7.3	6.1	1.6	10.5
Deliveries	Oct 2020	14.1	9.2	19.1	21.2	12.6	29.8	9.2	5.8	12.6	13.3	8.6	17.9
Deliveries	Nov 2020	21.4	16.6	26.1	32.1	22.2	41.9	13.9	10.3	17.5	20.1	15.5	24.7
Deliveries	Dec 2020	10.1	5.6	14.5	15.1	7.7	22.5	6.5	3.5	9.5	9.4	5.2	13.6
Deliveries	Total Covid Period	7.2	4.7	9.6	10.8	6.4	15.2	4.6	2.9	6.3	6.7	4.4	9.1
Penta1	Mar 2020	-10.0	-14.8	-5.3	-14.5	-21.8	-7.1	-7.1	-10.7	-3.6	-9.3	-13.8	-4.9
Penta1	Apr 2020	13.7	8.4	18.9	19.7	11.2	28.2	9.7	5.7	13.7	12.7	7.7	17.6
Penta1	May 2020	6.3	1.6	11.1	9.1	2.1	16.2	4.5	1.1	7.9	5.9	1.5	10.3
Penta1	Jun 2020	11.7	6.5	17.0	16.9	8.7	25.1	8.3	4.4	12.2	10.9	6.0	15.8
Penta1	Jul 2020	8.9	3.7	14.1	12.8	4.9	20.7	6.3	2.5	10.1	8.2	3.4	13.1
Penta1	Aug 2020	8.5	3.3	13.7	12.2	4.4	20.1	6.0	2.3	9.8	7.9	3.1	12.7
Penta1	Sep 2020	6.1	0.9	11.3	8.8	1.2	16.4	4.3	0.6	8.0	5.7	0.9	10.5
Penta1	Oct 2020	-1.5	-6.6	3.7	-2.1	-9.5	5.3	-1.0	-4.7	2.6	-1.4	-6.1	3.4
Penta1	Nov 2020	11.7	6.9	16.6	16.9	9.2	24.6	8.3	4.7	11.9	10.9	6.4	15.4
Penta1	Dec 2020	15.3	10.4	20.2	22.1	13.9	30.4	10.9	7.1	14.7	14.2	9.6	18.8
Penta1	Total Covid Period	6.9	4.3	9.5	10.0	5.7	14.3	4.9	2.9	6.8	6.4	3.9	8.8
Penta3	Mar 2020	-9.7	-14.7	-4.8	-14.1	-21.8	-6.4	-6.8	-10.4	-3.2	-9.1	-13.7	-4.4
Penta3	Apr 2020	14.9	9.3	20.5	21.7	12.5	30.8	10.4	6.2	14.5	13.9	8.7	19.1
Penta3	May 2020	8.4	3.4	13.4	12.2	4.5	19.8	5.8	2.3	9.4	7.8	3.1	12.5
Penta3	Jun 2020	18.6	13.0	24.3	27.1	17.3	36.9	13.0	8.6	17.3	17.3	12.0	22.7
Penta3	Jul 2020	10.2	4.7	15.7	14.8	6.4	23.3	7.1	3.2	11.0	9.5	4.4	14.6
Penta3	Aug 2020	10.2	4.7	15.6	14.7	6.3	23.2	7.1	3.1	11.0	9.5	4.3	14.6
Penta3	Sep 2020	11.7	6.2	17.2	17.0	8.4	25.7	8.1	4.2	12.1	10.9	5.8	16.1
Penta3	Oct 2020	7.6	2.1	13.0	11.0	2.8	19.2	5.3	1.4	9.1	7.0	2.0	12.1
Penta3	Nov 2020	10.3	5.3	15.3	14.9	7.1	22.8	7.2	3.5	10.8	9.6	4.9	14.3
Penta3	Dec 2020	9.7	4.7	14.7	14.1	6.3	21.9	6.8	3.1	10.4	9.0	4.3	13.7
Penta3	Total Covid Period	8.3	5.5	11.1	12.1	7.3	16.9	5.7	3.7	7.8	7.7	5.1	10.3
C-section	Mar 2020	6.3	-14.8	27.5	21.2	-58.6	101.0	2.1	-4.8	8.9	6.6	-15.6	28.8
C-section	Apr 2020	2.2	-20.7	25.1	7.3	-70.3	85.0	0.7	-6.8	8.2	2.3	-21.7	26.3
C-section	May 2020	32.3	8.7	55.9	109.7	-99.5	318.9	10.5	3.0	17.9	33.8	7.6	60.1
C-section	Jun 2020	21.7	-2.4	45.7	73.1	-78.5	224.7	7.0	-0.7	14.8	22.7	-3.2	48.6
C-section	Jul 2020	19.5	-4.4	43.4	65.9	-74.6	206.3	6.3	-1.3	14.0	20.4	-5.1	46.0
C-section	Aug 2020	18.7	-5.1	42.5	63.0	-73.2	199.2	6.1	-1.6	13.7	19.6	-5.9	45.0
C-section	Sep 2020	41.9	15.1	68.7	143.2	-127.0	413.3	13.6	5.2	21.9	43.9	13.5	74.3
C-section	Oct 2020	40.8	14.2	67.5	139.4	-124.0	402.7	13.2	4.9	21.5	42.7	12.7	72.8
C-section	Nov 2020	36.5	12.3	60.8	124.4	-110.4	359.1	11.8	4.3	19.4	38.2	10.9	65.5
C-section	Dec 2020	14.1	-7.5	35.7	47.4	-62.5	157.3	4.6	-2.4	11.5	14.8	-8.1	37.7
C-section	Total Covid Period	24.3	9.7	38.9	93.9	-113.2	300.9	7.6	3.4	11.9	25.5	8.6	42.4
Measles vaccination	Mar 2020	-20.6	-26.0	-15.2	-29.1	-38.3	-19.9	-15.0	-19.4	-10.5	-19.1	-24.2	-14.0
Measles vaccination	Apr 2020	11.0	5.3	16.8	15.6	7.0	24.2	8.0	3.7	12.3	10.2	4.9	15.6
Measles vaccination	May 2020	-0.2	-5.3	5.0	-0.2	-7.5	7.1	-0.1	-3.9	3.6	-0.1	-4.9	4.6
Measles vaccination	Jun 2020	8.6	2.9	14.3	12.2	3.9	20.6	6.3	2.0	10.5	8.0	2.7	13.3
Measles vaccination	Jul 2020	1.2	-4.4	6.8	1.7	-6.3	9.7	0.9	-3.2	5.0	1.1	-4.1	6.4
Measles vaccination	Aug 2020	2.2	-3.4	7.8	3.1	-4.9	11.1	1.6	-2.5	5.7	2.1	-3.2	7.3
Measles vaccination	Sep 2020	9.9	4.2	15.6	14.0	5.5	22.5	7.2	2.9	11.5	9.2	3.9	14.5
Measles vaccination	Oct 2020	2.3	-3.4	7.9	3.2	-4.8	11.2	1.7	-2.4	5.8	2.1	-3.1	7.4
Measles vaccination	Nov 2020	-3.1	-8.2	2.1	-4.3	-11.7	3.0	-2.2	-6.0	1.5	-2.8	-7.6	2.0
Measles vaccination	Dec 2020	5.6	0.4	10.8	7.9	0.4	15.4	4.1	0.3	7.9	5.2	0.4	10.0
Measles vaccination	Total Covid Period	0.3	-2.5	3.0	0.4	-3.5	4.3	0.2	-1.8	2.2	0.2	-2.3	2.8
OPD	Mar 2020	-4.8	-11.4	1.9	-7.9	-19.0	3.3	-2.6	-6.3	1.0	-4.6	-10.9	1.8
OPD	Apr 2020	-8.2	-15.4	-1.0	-13.6	-26.0	-1.1	-4.5	-8.5	-0.5	-7.9	-14.8	-0.9
OPD	May 2020	-6.9	-13.5	-0.3	-11.4	-22.7	-0.1	-3.8	-7.5	-0.1	-6.6	-13.0	-0.2
OPD	Jun 2020	-3.6	-10.8	3.7	-5.9	-17.9	6.2	-2.0	-5.9	2.0	-3.4	-10.3	3.5
OPD	Jul 2020	-16.5	-23.8	-9.1	-27.2	-41.1	-13.4	-9.1	-13.2	-4.9	-15.8	-22.9	-8.7
OPD	Aug 2020	-2.1	-9.3	5.1	-3.4	-15.4	8.6	-1.1	-5.1	2.8	-2.0	-8.9	4.9
OPD	Sep 2020	12.0	4.6	19.3	19.9	6.7	33.1	6.6	2.5	10.7	11.5	4.4	18.6
OPD	Oct 2020	22.9	15.3	30.5	38.0	22.1	54.0	12.5	8.2	16.9	21.9	14.5	29.3
OPD	Nov 2020	39.7	32.0	47.3	66.2	44.9	87.4	21.7	16.9	26.5	38.0	30.3	45.8
OPD	Dec 2020	3.5	-3.1	10.2	5.8	-5.3	17.0	1.9	-1.7	5.6	3.4	-3.0	9.8
OPD	Total Covid Period	4.6	0.9	8.3	7.6	1.1	14.1	2.5	0.5	4.5	4.4	0.8	7.9
IPD	Mar 2020	-3.8	-12.9	5.2	-8.3	-28.7	12.1	-1.8	-6.2	2.5	-3.6	-12.1	4.9
IPD	Apr 2020	-5.6	-15.5	4.3	-12.3	-35.1	10.6	-2.7	-7.5	2.1	-5.3	-14.6	4.0
IPD	May 2020	0.6	-8.4	9.7	1.4	-18.4	21.1	0.3	-4.1	4.7	0.6	-7.9	9.1
IPD	Jun 2020	4.4	-5.5	14.3	9.6	-12.9	32.1	2.1	-2.7	6.9	4.1	-5.2	13.4
IPD	Jul 2020	-2.5	-12.3	7.4	-5.4	-27.1	16.4	-1.2	-6.0	3.6	-2.3	-11.6	7.0
IPD	Aug 2020	4.3	-5.7	14.2	9.3	-13.1	31.8	2.1	-2.7	6.9	4.0	-5.3	13.3
IPD	Sep 2020	18.2	7.7	28.6	39.7	5.5	74.0	8.7	3.5	13.9	17.0	7.0	27.0
IPD	Oct 2020	16.2	5.9	26.6	35.5	3.4	67.6	7.8	2.7	12.9	15.2	5.4	25.0
IPD	Nov 2020	47.8	35.4	60.2	105.5	31.5	179.4	22.9	15.9	29.9	44.7	32.0	57.4
IPD	Dec 2020	15.6	6.1	25.1	34.1	3.9	64.3	7.5	2.8	12.2	14.6	5.5	23.7
IPD	Total Covid Period	11.0	5.5	16.4	24.6	3.9	45.3	5.2	2.5	7.9	10.2	5.1	15.4

COTE D'IVOIRE

		National			Urban			Mixed urban-rural		
Indicator	Month/Period	%change	Lower bound	Upper bound	%change	Lower bound	Upper bound	%change	Lower bound	Upper bound
ANC1	Mar 2020	-5.8	-11.1	-0.6	-2.0	-3.9	-0.2	-7.1	-13.5	-0.7
ANC1	Apr 2020	10.5	5.1	15.9	3.7	1.8	5.5	12.8	6.2	19.4
ANC1	May 2020	-5.5	-10.8	-0.2	-1.9	-3.8	-0.1	-6.7	-13.1	-0.3
ANC1	Jun 2020	7.5	2.2	12.8	2.6	0.7	4.5	9.1	2.6	15.6
ANC1	Jul 2020	-7.6	-12.9	-2.3	-2.6	-4.5	-0.8	-9.2	-15.7	-2.7
ANC1	Aug 2020	-4.3	-9.6	1.0	-1.5	-3.3	0.3	-5.2	-11.6	1.2
ANC1	Sep 2020	6.7	1.4	12.1	2.3	0.5	4.2	8.2	1.7	14.7
ANC1	Oct 2020	-2.9	-8.2	2.4	-1.0	-2.9	0.8	-3.6	-10.0	2.9
ANC1	Nov 2020	3.9	-1.4	9.2	1.3	-0.5	3.2	4.7	-1.8	11.1
ANC1	Dec 2020	1.5	-3.8	6.8	0.5	-1.3	2.4	1.8	-4.6	8.2
**ANC1**	Total Covid Period	0.4	-2.0	2.7	0.1	-0.7	1.0	0.5	-2.4	3.3
ANC4	Mar 2020	-10.3	-14.6	-5.9	-4.1	-5.8	-2.3	-12.0	-17.1	-6.8
ANC4	Apr 2020	-4.1	-8.4	0.2	-1.6	-3.3	0.1	-4.8	-9.8	0.2
ANC4	May 2020	-7.9	-12.2	-3.6	-3.1	-4.9	-1.4	-9.2	-14.3	-4.1
ANC4	Jun 2020	-1.8	-6.1	2.5	-0.7	-2.4	1.0	-2.1	-7.1	2.9
ANC4	Jul 2020	-4.6	-9.0	-0.3	-1.8	-3.5	-0.1	-5.4	-10.5	-0.4
ANC4	Aug 2020	-5.6	-10.0	-1.3	-2.2	-3.9	-0.5	-6.6	-11.7	-1.5
ANC4	Sep 2020	2.8	-1.5	7.1	1.1	-0.6	2.8	3.3	-1.8	8.4
ANC4	Oct 2020	-3.5	-7.8	0.8	-1.4	-3.1	0.3	-4.1	-9.1	1.0
ANC4	Nov 2020	-1.5	-5.8	2.8	-0.6	-2.3	1.1	-1.7	-6.8	3.3
ANC4	Dec 2020	1.2	-3.1	5.5	0.5	-1.2	2.2	1.4	-3.6	6.5
ANC4	Total Covid Period	-3.6	-5.5	-1.6	-1.4	-2.2	-0.6	-4.2	-6.4	-1.9
Deliveries	Mar 2020	-2.5	-6.2	1.3	-1.2	-2.9	0.6	-2.8	-7.0	1.5
Deliveries	Apr 2020	0.5	-3.2	4.3	0.3	-1.5	2.0	0.6	-3.6	4.8
Deliveries	May 2020	-4.3	-8.1	-0.5	-2.0	-3.8	-0.2	-4.8	-9.1	-0.6
Deliveries	Jun 2020	-6.0	-9.8	-2.2	-2.8	-4.7	-1.0	-6.8	-11.0	-2.5
Deliveries	Jul 2020	1.0	-2.7	4.8	0.5	-1.3	2.3	1.2	-3.1	5.4
Deliveries	Aug 2020	-4.5	-8.3	-0.7	-2.1	-3.9	-0.3	-5.1	-9.3	-0.8
Deliveries	Sep 2020	-1.5	-5.3	2.2	-0.7	-2.5	1.1	-1.7	-5.9	2.5
Deliveries	Oct 2020	-0.3	-4.1	3.5	-0.2	-1.9	1.6	-0.4	-4.6	3.9
Deliveries	Nov 2020	2.1	-1.6	5.9	1.0	-0.8	2.8	2.4	-1.8	6.7
Deliveries	Dec 2020	-5.1	-8.9	-1.3	-2.4	-4.2	-0.6	-5.7	-10.0	-1.4
Deliveries	Total Covid Period	-2.1	-3.7	-0.4	-1.0	-1.8	-0.2	-2.3	-4.2	-0.4
C-section	Mar 2020	-1.2	-12.0	9.6	-0.4	-3.9	3.1	-1.5	-14.9	12.0
C-section	Apr 2020	-11.4	-22.8	0.0	-3.7	-7.4	0.0	-14.1	-28.7	0.4
C-section	May 2020	-6.8	-17.8	4.2	-2.2	-5.7	1.4	-8.4	-22.2	5.4
C-section	Jun 2020	-6.4	-17.4	4.6	-2.1	-5.6	1.5	-7.9	-21.7	5.9
C-section	Jul 2020	-7.4	-18.4	3.7	-2.4	-6.0	1.2	-9.1	-23.1	4.8
C-section	Aug 2020	0.9	-9.9	11.8	0.3	-3.2	3.8	1.2	-12.3	14.6
C-section	Sep 2020	-8.0	-19.1	3.1	-2.6	-6.2	1.0	-9.9	-24.0	4.1
C-section	Oct 2020	-3.3	-14.2	7.6	-1.1	-4.6	2.4	-4.1	-17.7	9.5
C-section	Nov 2020	0.7	-10.2	11.6	0.2	-3.3	3.7	0.9	-12.7	14.4
C-section	Dec 2020	-6.8	-17.9	4.3	-2.2	-5.8	1.4	-8.4	-22.4	5.5
C-section	Total Covid Period	-4.9	-10.0	0.1	-1.6	-3.3	0.1	-6.1	-12.6	0.3
OPD	Mar 2020	-20.0	-24.0	-16.0	-9.3	-11.4	-7.2	-22.5	-27.1	-17.9
OPD	Apr 2020	-7.0	-10.6	-3.3	-3.2	-5.0	-1.5	-7.8	-12.0	-3.7
OPD	May 2020	-13.8	-17.6	-9.9	-6.4	-8.3	-4.5	-15.4	-19.8	-11.1
OPD	Jun 2020	-18.3	-22.2	-14.3	-8.5	-10.5	-6.4	-20.5	-25.0	-15.9
OPD	Jul 2020	-10.9	-14.7	-7.2	-5.1	-6.9	-3.3	-12.3	-16.5	-8.0
OPD	Aug 2020	-26.6	-30.8	-22.3	-12.3	-14.7	-10.0	-29.8	-34.8	-24.8
OPD	Sep 2020	-4.7	-8.4	-1.1	-2.2	-3.9	-0.5	-5.3	-9.5	-1.2
OPD	Oct 2020	-6.8	-10.5	-3.1	-3.1	-4.9	-1.4	-7.6	-11.8	-3.5
OPD	Nov 2020	-7.9	-11.6	-4.2	-3.7	-5.4	-1.9	-8.9	-13.0	-4.7
OPD	Dec 2020	-10.4	-14.1	-6.6	-4.8	-6.6	-3.0	-11.6	-15.9	-7.4
OPD	Total Covid Period	-12.4	-14.2	-10.5	-5.8	-6.9	-4.8	-13.8	-16.0	-11.6
IPD	Mar 2020	-9.7	-20.1	0.7	-7.2	-15.7	1.3	-10.0	-20.7	0.8
IPD	Apr 2020	6.2	-4.1	16.6	4.6	-3.4	12.6	6.4	-4.3	17.1
IPD	May 2020	-7.1	-17.5	3.2	-5.3	-13.4	2.8	-7.4	-18.0	3.3
IPD	Jun 2020	-5.7	-16.0	4.6	-4.2	-12.2	3.7	-5.9	-16.5	4.7
IPD	Jul 2020	-0.9	-11.2	9.3	-0.7	-8.3	6.9	-0.9	-11.5	9.6
IPD	Aug 2020	-17.7	-28.6	-6.8	-13.2	-23.6	-2.7	-18.3	-29.6	-6.9
IPD	Sep 2020	3.5	-6.8	13.9	2.6	-5.1	10.4	3.7	-7.0	14.3
IPD	Oct 2020	2.6	-7.7	12.9	1.9	-5.8	9.6	2.6	-8.0	13.3
IPD	Nov 2020	3.7	-6.6	14.0	2.7	-5.0	10.5	3.8	-6.9	14.5
IPD	Dec 2020	-2.4	-12.7	7.9	-1.8	-9.5	5.9	-2.5	-13.1	8.2
IPD	Total Covid Period	-2.8	-7.4	1.8	-2.1	-5.6	1.5	-2.9	-7.6	1.8

ETHIOPIA

		National			Ethiopia without Addis			Addis Ababa Only		
indicator	Month/period	Drop (%)	lb_drop	ub_drop	Drop (%)	lb_drop	ub_drop	Drop (%)	lb_drop	ub_drop
ANC1	Mar-20	-13.0	-24.3	-1.7	-12.5	-23.3	-1.7	-24.9	-46.5	-3.3
ANC1	Apr-20	-17.2	-28.5	-6.0	-16.5	-27.3	-5.7	-32.9	-54.4	-11.4
ANC1	May-20	-1.1	-12.5	10.3	-1.0	-12.0	9.9	-2.1	-24.0	19.9
ANC1	Jun-20	-2.4	-13.8	9.0	-2.3	-13.2	8.6	-4.6	-26.5	17.3
ANC1	Jul-20	-9.2	-20.5	2.1	-8.8	-19.7	2.0	-17.6	-39.3	4.0
ANC1	Aug-20	-3.5	-14.8	7.9	-3.3	-14.2	7.6	-6.6	-28.5	15.2
ANC1	Sep-20	-4.5	-15.9	6.8	-4.3	-15.2	6.6	-8.7	-30.5	13.1
**ANC1**	**Total Covid Period**	**-7.1**	**-14.2**	**-0.1**	**-6.8**	**-13.6**	**-0.1**	**-13.4**	**-26.5**	**-0.4**
ANC4	Mar-20	-5.5	-13.5	2.5	-5.4	-13.1	2.4	-8.7	-21.2	3.9
ANC4	Apr-20	-10.9	-18.8	-2.9	-10.5	-18.2	-2.8	-17.0	-29.5	-4.5
ANC4	May-20	-3.6	-11.7	4.4	-3.5	-11.3	4.3	-5.7	-18.3	6.9
ANC4	Jun-20	-4.7	-12.8	3.3	-4.6	-12.4	3.2	-7.4	-20.0	5.1
ANC4	Jul-20	-5.2	-13.2	2.8	-5.0	-12.8	2.7	-8.2	-20.7	4.4
ANC4	Aug-20	-2.1	-10.1	5.9	-2.0	-9.8	5.7	-3.3	-15.9	9.3
ANC4	Sep-20	-1.8	-9.8	6.2	-1.7	-9.5	6.0	-2.8	-15.4	9.8
**ANC4**	**Total Covid Period**	**-4.8**	**-9.8**	**0.2**	**-4.6**	**-9.5**	**0.2**	**-7.4**	**-15.1**	**0.3**
Deliveries	Mar-20	0.8	-4.9	6.4	0.8	-4.8	6.3	1.0	-6.3	8.3
Deliveries	Apr-20	-3.3	-8.9	2.3	-3.3	-8.8	2.2	-4.3	-11.6	2.9
Deliveries	May-20	0.6	-5.0	6.2	0.6	-4.9	6.1	0.8	-6.5	8.1
Deliveries	Jun-20	2.2	-3.4	7.9	2.2	-3.3	7.7	2.9	-4.4	10.2
Deliveries	Jul-20	-1.3	-6.9	4.3	-1.3	-6.8	4.2	-1.7	-9.0	5.6
Deliveries	Aug-20	-0.5	-6.1	5.2	-0.5	-6.0	5.0	-0.6	-7.9	6.7
Deliveries	Sep-20	-8.2	-13.8	-2.7	-8.1	-13.5	-2.6	-10.7	-17.9	-3.4
**Deliveries**	**Total Covid Period**	**-1.4**	**-5.0**	**2.2**	**-1.4**	**-4.9**	**2.2**	**-1.8**	**-6.5**	**2.9**
Penta1	Mar-20	-11.4	-18.6	-4.2	-10.9	-17.8	-4.0	-22.3	-36.5	-8.0
Penta1	Apr-20	-7.7	-14.9	-0.4	-7.4	-14.3	-0.4	-15.0	-29.3	-0.8
Penta1	May-20	1.8	-5.5	9.1	1.7	-5.3	8.7	3.6	-10.8	18.0
Penta1	Jun-20	-2.7	-9.9	4.6	-2.5	-9.5	4.4	-5.2	-19.5	9.1
Penta1	Jul-20	-3.2	-10.5	4.0	-3.1	-10.1	3.9	-6.4	-20.6	7.9
Penta1	Aug-20	-2.0	-9.3	5.3	-1.9	-8.9	5.0	-4.0	-18.3	10.3
Penta1	Sep-20	-6.5	-13.7	0.8	-6.2	-13.2	0.8	-12.7	-26.9	1.6
**Penta1**	**Total Covid Period**	**-4.5**	**-9.1**	**0.2**	**-4.3**	**-8.7**	**0.1**	**-8.7**	**-17.5**	**0.2**
Penta3	Mar-20	-7.7	-15.0	-0.3	-7.4	-14.4	-0.3	-14.1	-27.8	-0.5
Penta3	Apr-20	-10.0	-17.4	-2.6	-9.6	-16.7	-2.5	-18.4	-32.1	-4.8
Penta3	May-20	-3.1	-10.5	4.3	-3.0	-10.1	4.1	-5.7	-19.4	7.9
Penta3	Jun-20	-3.1	-10.5	4.3	-2.9	-10.1	4.2	-5.7	-19.3	8.0
Penta3	Jul-20	-2.5	-9.9	4.9	-2.4	-9.5	4.7	-4.6	-18.3	9.0
Penta3	Aug-20	-1.1	-8.5	6.3	-1.1	-8.2	6.1	-2.1	-15.8	11.7
Penta3	Sep-20	-3.9	-11.3	3.5	-3.8	-10.9	3.4	-7.2	-20.9	6.4
**Penta3**	**Total Covid Period**	**-4.4**	**-9.1**	**0.2**	**-4.3**	**-8.7**	**0.2**	**-8.1**	**-16.5**	**0.3**
C-section	Mar-20	7.2	-3.5	18.0	8.9	-4.3	22.1	2.4	-1.1	5.9
C-section	Apr-20	3.0	-7.7	13.7	3.7	-9.4	16.8	1.0	-2.5	4.5
C-section	May-20	4.5	-6.2	15.2	5.5	-7.6	18.6	1.5	-2.0	5.0
C-section	Jun-20	10.6	-0.2	21.4	13.0	-0.2	26.3	3.5	0.0	7.0
C-section	Jul-20	4.7	-6.0	15.4	5.7	-7.4	18.9	1.5	-2.0	5.0
C-section	Aug-20	-5.9	-16.5	4.7	-7.3	-20.3	5.7	-2.0	-5.4	1.5
C-section	Sep-20	2.1	-8.5	12.8	2.6	-10.5	15.7	0.7	-2.8	4.2
**C-section**	**Total Covid Period**	**3.8**	**-3.3**	**10.8**	**4.6**	**-4.1**	**13.3**	**1.2**	**-1.0**	**3.5**
Measles vaccination	Mar-20	-14.8	-26.4	-3.2	-14.2	-25.3	-3.0	-28.0	-50.1	-5.9
Measles vaccination	Apr-20	-8.0	-19.7	3.7	-7.7	-18.9	3.5	-15.3	-37.5	7.0
Measles vaccination	May-20	6.2	-5.7	18.1	5.9	-5.4	17.3	11.9	-10.9	34.7
Measles vaccination	Jun-20	-0.2	-12.0	11.6	-0.2	-11.5	11.1	-0.3	-22.8	22.2
Measles vaccination	Jul-20	-5.9	-17.6	5.8	-5.7	-16.9	5.6	-11.2	-33.5	11.1
Measles vaccination	Aug-20	2.5	-9.3	14.4	2.4	-8.9	13.8	4.8	-17.8	27.5
Measles vaccination	Sep-20	-2.4	-14.2	9.4	-2.3	-13.6	9.0	-4.6	-27.0	17.8
**Measles vaccination**	**Total Covid Period**	**-3.2**	**-10.7**	**4.3**	**-3.1**	**-10.3**	**4.1**	**-6.1**	**-20.1**	**8.0**
OPD	Mar-20	-13.6	-25.7	-1.6	-13.6	-25.7	-1.6	-13.5	-25.6	-1.5
OPD	Apr-20	-32.6	-44.6	-20.6	-32.6	-44.6	-20.6	-32.4	-44.7	-20.1
OPD	May-20	-26.7	-38.7	-14.7	-26.7	-38.7	-14.8	-26.6	-38.8	-14.4
OPD	Jun-20	-19.9	-31.9	-7.9	-19.9	-31.9	-7.9	-19.8	-31.9	-7.7
OPD	Jul-20	-25.9	-37.9	-14.0	-26.0	-37.9	-14.0	-25.8	-38.0	-13.6
OPD	Aug-20	-26.2	-38.2	-14.2	-26.2	-38.2	-14.2	-26.0	-38.2	-13.9
OPD	Sep-20	-25.9	-37.8	-13.9	-25.9	-37.9	-13.9	-25.7	-37.9	-13.5
**OPD**	**Total Covid Period**	**-22.8**	**-29.7**	**-16.0**	**-22.8**	**-29.7**	**-16.0**	**-22.7**	**-29.9**	**-15.6**
IPD	Mar-20	-17.4	-34.5	-0.2	-18.0	-35.8	-0.2	-12.4	-24.6	-0.1
IPD	Apr-20	-38.4	-55.2	-21.6	-39.8	-57.2	-22.4	-27.4	-39.5	-15.3
IPD	May-20	-17.6	-34.7	-0.5	-18.3	-36.0	-0.5	-12.5	-24.8	-0.3
IPD	Jun-20	-28.9	-45.9	-12.0	-30.0	-47.6	-12.5	-20.7	-32.8	-8.5
IPD	Jul-20	-24.8	-41.8	-7.8	-25.8	-43.4	-8.1	-17.7	-29.9	-5.5
IPD	Aug-20	-31.2	-48.1	-14.3	-32.4	-49.9	-14.9	-22.3	-34.4	-10.1
IPD	Sep-20	-31.2	-48.1	-14.3	-32.4	-49.9	-14.8	-22.3	-34.4	-10.1
**IPD**	**Total Covid Period**	**-25.1**	**-34.6**	**-15.7**	**-26.0**	**-35.7**	**-16.3**	**-18.3**	**-25.4**	**-11.2**

GHANA

Indicator	Month/Period	National %change	National Lower bound	National Upper bound	Rural %change	Rural Lower bound	Rural Upper bound	Urban %change	Urban Lower bound	Urban Upper bound
ANC1	Mar 2020	-0.4	-2.9	2.1	-0.5	-3.3	2.3	-0.2	-1.7	1.2
ANC1	Apr 2020	-0.4	-2.9	2.0	-0.5	-3.3	2.3	-0.3	-1.7	1.2
ANC1	May 2020	-3.7	-6.2	-1.2	-4.2	-7.0	-1.4	-2.2	-3.7	-0.7
ANC1	Jun 2020	14.4	11.6	17.2	16.4	13.1	19.8	8.6	6.8	10.5
ANC1	Jul 2020	2.2	-0.2	4.7	2.5	-0.3	5.4	1.3	-0.1	2.8
ANC1	Aug 2020	1.1	-1.4	3.6	1.3	-1.5	4.1	0.7	-0.8	2.1
ANC1	Sep 2020	3.7	1.2	6.1	4.2	1.3	7.0	2.2	0.7	3.7
ANC1	Oct 2020	4.5	2.0	7.0	5.1	2.3	8.0	2.7	1.2	4.2
ANC1	Nov 2020	8.3	5.7	10.8	9.4	6.4	12.4	4.9	3.3	6.6
ANC1	Dec 2020	16.3	13.5	19.2	18.6	15.1	22.1	9.8	7.8	11.7
ANC1	Total Covid Period	4.6	3.4	5.8	5.3	3.9	6.6	2.8	2.0	3.5
ANC4	Mar 2020	-3.3	-7.3	0.7	-3.9	-8.5	0.8	-1.8	-4.0	0.4
ANC4	Apr 2020	-8.3	-12.4	-4.3	-9.8	-14.6	-5.0	-4.6	-6.9	-2.3
ANC4	May 2020	-11.1	-15.2	-7.0	-13.0	-17.9	-8.0	-6.1	-8.4	-3.8
ANC4	Jun 2020	-0.1	-4.0	3.9	-0.1	-4.8	4.5	0.0	-2.2	2.1
ANC4	Jul 2020	2.3	-1.7	6.3	2.7	-1.9	7.4	1.3	-0.9	3.5
ANC4	Aug 2020	-0.3	-4.2	3.7	-0.3	-4.9	4.4	-0.1	-2.3	2.0
ANC4	Sep 2020	1.8	-2.2	5.8	2.1	-2.5	6.8	1.0	-1.2	3.2
ANC4	Oct 2020	1.7	-2.3	5.6	2.0	-2.7	6.6	0.9	-1.3	3.1
ANC4	Nov 2020	2.1	-1.8	6.1	2.5	-2.2	7.2	1.2	-1.0	3.4
ANC4	Dec 2020	11.7	7.5	15.9	13.7	8.7	18.8	6.4	4.1	8.8
ANC4	Total Covid Period	-0.4	-2.1	1.3	-0.4	-2.4	1.6	-0.2	-1.1	0.7
Deliveries	Mar 2020	-3.6	-6.6	-0.6	-4.3	-7.9	-0.7	-1.9	-3.5	-0.3
Deliveries	Apr 2020	-5.1	-8.2	-2.1	-6.1	-9.7	-2.5	-2.7	-4.4	-1.1
Deliveries	May 2020	-5.7	-8.8	-2.7	-6.8	-10.4	-3.1	-3.1	-4.7	-1.4
Deliveries	Jun 2020	-3.4	-6.4	-0.4	-4.0	-7.6	-0.4	-1.8	-3.4	-0.2
Deliveries	Jul 2020	2.0	-1.0	5.0	2.3	-1.2	5.9	1.1	-0.6	2.7
Deliveries	Aug 2020	-4.3	-7.3	-1.3	-5.1	-8.7	-1.5	-2.3	-3.9	-0.7
Deliveries	Sep 2020	-5.3	-8.3	-2.2	-6.2	-9.9	-2.6	-2.8	-4.5	-1.2
Deliveries	Oct 2020	-2.4	-5.4	0.6	-2.8	-6.4	0.8	-1.3	-2.9	0.3
Deliveries	Nov 2020	1.4	-1.6	4.4	1.6	-1.9	5.2	0.7	-0.9	2.3
Deliveries	Dec 2020	-4.5	-7.6	-1.5	-5.4	-9.0	-1.8	-2.4	-4.1	-0.8
Deliveries	Total Covid Period	-3.1	-4.4	-1.8	-3.7	-5.2	-2.1	-1.7	-2.4	-0.9
Penta1	Mar 2020	-5.9	-8.5	-3.3	-6.8	-9.9	-3.7	-3.4	-4.9	-1.8
Penta1	Apr 2020	-3.8	-6.4	-1.2	-4.4	-7.4	-1.4	-2.2	-3.7	-0.7
Penta1	May 2020	-4.5	-7.2	-1.9	-5.2	-8.3	-2.2	-2.6	-4.1	-1.1
Penta1	Jun 2020	-0.1	-2.7	2.5	-0.1	-3.1	2.9	-0.1	-1.5	1.4
Penta1	Jul 2020	0.4	-2.2	3.0	0.5	-2.5	3.5	0.2	-1.3	1.7
Penta1	Aug 2020	-0.4	-3.0	2.2	-0.5	-3.5	2.5	-0.2	-1.7	1.3
Penta1	Sep 2020	-3.9	-6.5	-1.3	-4.5	-7.5	-1.5	-2.2	-3.8	-0.7
Penta1	Oct 2020	-3.6	-6.2	-1.0	-4.2	-7.2	-1.1	-2.1	-3.6	-0.6
Penta1	Nov 2020	-4.0	-6.6	-1.4	-4.6	-7.6	-1.6	-2.3	-3.8	-0.8
Penta1	Dec 2020	1.8	-0.8	4.4	2.1	-0.9	5.1	1.0	-0.5	2.5
Penta1	Total Covid Period	-2.4	-3.5	-1.3	-2.8	-4.1	-1.5	-1.4	-2.0	-0.7
Penta3	Mar 2020	-6.3	-8.9	-3.6	-7.2	-10.3	-4.2	-3.6	-5.1	-2.1
Penta3	Apr 2020	-7.9	-10.6	-5.3	-9.2	-12.3	-6.1	-4.6	-6.1	-3.0
Penta3	May 2020	-5.2	-7.8	-2.6	-6.0	-9.1	-3.0	-3.0	-4.5	-1.5
Penta3	Jun 2020	2.7	0.1	5.3	3.1	0.1	6.1	1.5	0.0	3.1
Penta3	Jul 2020	1.6	-1.0	4.2	1.8	-1.2	4.9	0.9	-0.6	2.4
Penta3	Aug 2020	-0.7	-3.3	1.9	-0.8	-3.8	2.2	-0.4	-1.9	1.1
Penta3	Sep 2020	-4.3	-7.0	-1.7	-5.0	-8.0	-2.0	-2.5	-4.0	-1.0
Penta3	Oct 2020	-4.4	-7.0	-1.8	-5.1	-8.1	-2.1	-2.5	-4.1	-1.0
Penta3	Nov 2020	-1.9	-4.5	0.7	-2.2	-5.2	0.8	-1.1	-2.6	0.4
Penta3	Dec 2020	0.7	-1.9	3.3	0.8	-2.3	3.8	0.4	-1.1	1.9
Penta3	Total Covid Period	-2.6	-3.7	-1.5	-3.0	-4.3	-1.7	-1.5	-2.2	-0.8
C-section	Mar 2020	-7.2	-13.0	-1.5	-9.6	-17.5	-1.8	-3.7	-6.6	-0.7
C-section	Apr 2020	-3.7	-9.3	2.0	-4.9	-12.5	2.8	-1.9	-4.7	1.0
C-section	May 2020	-7.7	-13.5	-2.0	-10.3	-18.2	-2.4	-3.9	-6.9	-1.0
C-section	Jun 2020	1.5	-4.2	7.2	2.0	-5.6	9.6	0.8	-2.1	3.6
C-section	Jul 2020	3.9	-1.8	9.7	5.2	-2.5	12.9	2.0	-0.9	4.9
C-section	Aug 2020	-7.3	-13.1	-1.5	-9.7	-17.6	-1.9	-3.7	-6.7	-0.8
C-section	Sep 2020	-5.3	-11.0	0.5	-7.0	-14.7	0.7	-2.7	-5.6	0.2
C-section	Oct 2020	1.8	-3.9	7.5	2.4	-5.3	10.0	0.9	-2.0	3.8
C-section	Nov 2020	0.3	-5.4	6.0	0.4	-7.1	8.0	0.2	-2.7	3.0
C-section	Dec 2020	-1.1	-6.8	4.6	-1.5	-9.0	6.1	-0.6	-3.4	2.3
C-section	Total Covid Period	-2.5	-4.9	0.0	-3.3	-6.6	0.0	-1.3	-2.5	0.0
Measles vaccination	Mar 2020	-12.1	-15.1	-9.1	-14.2	-17.7	-10.6	-6.7	-8.4	-5.0
Measles vaccination	Apr 2020	-16.6	-19.7	-13.5	-19.4	-23.2	-15.7	-9.2	-11.0	-7.4
Measles vaccination	May 2020	-4.6	-7.5	-1.8	-5.4	-8.8	-2.1	-2.6	-4.2	-1.0
Measles vaccination	Jun 2020	-1.5	-4.3	1.4	-1.7	-5.1	1.6	-0.8	-2.4	0.8
Measles vaccination	Jul 2020	-0.7	-3.6	2.1	-0.8	-4.2	2.5	-0.4	-2.0	1.2
Measles vaccination	Aug 2020	1.5	-1.3	4.4	1.8	-1.5	5.2	0.9	-0.7	2.4
Measles vaccination	Sep 2020	-8.7	-11.6	-5.8	-10.2	-13.7	-6.7	-4.8	-6.5	-3.2
Measles vaccination	Oct 2020	-0.7	-3.5	2.2	-0.8	-4.1	2.6	-0.4	-1.9	1.2
Measles vaccination	Nov 2020	-7.1	-10.0	-4.2	-8.3	-11.7	-4.9	-3.9	-5.5	-2.3
Measles vaccination	Dec 2020	-0.9	-3.8	1.9	-1.1	-4.4	2.3	-0.5	-2.1	1.1
Measles vaccination	Total Covid Period	-5.1	-6.4	-3.8	-6.0	-7.5	-4.5	-2.8	-3.6	-2.1
OPD	Mar 2020	-16.0	-19.5	-12.5	-18.5	-22.8	-14.2	-9.1	-11.2	-6.9
OPD	Apr 2020	-29.6	-34.0	-25.3	-34.3	-40.1	-28.6	-16.8	-19.8	-13.9
OPD	May 2020	-32.0	-36.6	-27.5	-37.1	-43.1	-31.1	-18.2	-21.3	-15.1
OPD	Jun 2020	-26.8	-31.0	-22.7	-31.1	-36.5	-25.7	-15.2	-18.0	-12.4
OPD	Jul 2020	-24.2	-28.2	-20.3	-28.1	-33.2	-23.0	-13.7	-16.4	-11.1
OPD	Aug 2020	-22.1	-25.9	-18.2	-25.6	-30.5	-20.7	-12.5	-15.0	-10.0
OPD	Sep 2020	-25.6	-29.6	-21.5	-29.6	-34.9	-24.3	-14.5	-17.2	-11.8
OPD	Oct 2020	-21.7	-25.6	-17.9	-25.2	-30.1	-20.3	-12.3	-14.8	-9.8
OPD	Nov 2020	-14.4	-17.9	-11.0	-16.7	-20.9	-12.5	-8.2	-10.3	-6.1
OPD	Dec 2020	-5.4	-8.5	-2.2	-6.2	-9.9	-2.6	-3.1	-4.9	-1.2
OPD	Total Covid Period	-21.0	-23.4	-18.5	-24.1	-27.5	-20.8	-12.1	-13.9	-10.3
IPD	Mar 2020	-11.1	-15.7	-6.6	-13.2	-18.8	-7.7	-5.8	-8.3	-3.4
IPD	Apr 2020	-25.5	-30.9	-20.2	-30.3	-37.4	-23.2	-13.4	-16.6	-10.3
IPD	May 2020	-28.0	-33.6	-22.4	-33.3	-40.7	-25.8	-14.7	-18.0	-11.4
IPD	Jun 2020	-23.8	-29.1	-18.6	-28.3	-35.2	-21.4	-12.5	-15.5	-9.5
IPD	Jul 2020	-18.7	-23.6	-13.8	-22.2	-28.5	-15.9	-9.8	-12.6	-7.0
IPD	Aug 2020	-17.6	-22.4	-12.7	-20.9	-27.0	-14.7	-9.2	-11.9	-6.5
IPD	Sep 2020	-23.9	-29.1	-18.6	-28.4	-35.3	-21.4	-12.6	-15.6	-9.5
IPD	Oct 2020	-14.4	-19.1	-9.8	-17.2	-23.0	-11.3	-7.6	-10.2	-5.0
IPD	Nov 2020	-5.2	-9.6	-0.8	-6.2	-11.5	-1.0	-2.7	-5.1	-0.4
IPD	Dec 2020	-6.8	-11.3	-2.4	-8.1	-13.5	-2.8	-3.6	-5.9	-1.2
IPD	Total Covid Period	-17.0	-19.7	-14.3	-20.1	-23.8	-16.3	-9.1	-10.8	-7.3

KENYA

		National			Rural			Urban		
Indicator	Month/Period	%change	Lower bound	Upper bound	%change	Lower bound	Upper bound	%change	Lower bound	Upper bound
ANC1	Mar 2020	1.9	-2.3	6.1	2.1	-2.6	6.9	0.5	-0.7	1.7
ANC1	Apr 2020	-4.8	-9.0	-0.6	-5.4	-10.1	-0.7	-1.4	-2.6	-0.2
ANC1	May 2020	-3.4	-7.6	0.8	-3.8	-8.5	0.9	-1.0	-2.2	0.2
ANC1	Jun 2020	10.4	6.2	14.6	11.7	6.9	16.5	3.0	1.8	4.2
ANC1	Jul 2020	-1.6	-5.8	2.6	-1.8	-6.5	2.9	-0.5	-1.7	0.7
ANC1	Aug 2020	-0.7	-4.9	3.5	-0.8	-5.5	3.9	-0.2	-1.4	1.0
ANC1	Sep 2020	3.0	-1.2	7.2	3.4	-1.4	8.1	0.9	-0.3	2.1
ANC1	Oct 2020	-1.4	-5.6	2.8	-1.6	-6.3	3.1	-0.4	-1.6	0.8
ANC1	Nov 2020	3.2	-1.0	7.4	3.6	-1.1	8.3	0.9	-0.3	2.1
ANC1	Dec 2020	3.8	-0.4	8.0	4.3	-0.5	9.0	1.1	-0.1	2.3
**ANC1**	**Total Covid Period**	**1.0**	**-1.0**	**3.1**	**1.2**	**-1.1**	**3.5**	**0.3**	**-0.3**	**0.9**
ANC4	Mar 2020	-20.4	-26.1	-14.6	-23.6	-30.3	-16.9	-5.0	-6.4	-3.6
ANC4	Apr 2020	2.8	-3.0	8.6	3.2	-3.5	10.0	0.7	-0.7	2.1
ANC4	May 2020	-11.8	-17.5	-6.0	-13.6	-20.3	-7.0	-2.9	-4.3	-1.5
ANC4	Jun 2020	-13.6	-19.4	-7.9	-15.8	-22.5	-9.1	-3.3	-4.7	-1.9
ANC4	Jul 2020	-3.2	-9.0	2.6	-3.7	-10.4	3.0	-0.8	-2.2	0.6
ANC4	Aug 2020	-8.5	-14.2	-2.7	-9.8	-16.5	-3.1	-2.1	-3.5	-0.7
ANC4	Sep 2020	-3.0	-8.7	2.8	-3.4	-10.1	3.3	-0.7	-2.1	0.7
ANC4	Oct 2020	-12.3	-18.1	-6.5	-14.3	-21.0	-7.6	-3.0	-4.4	-1.6
ANC4	Nov 2020	-12.3	-18.0	-6.5	-14.2	-20.9	-7.5	-3.0	-4.4	-1.6
ANC4	Dec 2020	-9.1	-14.9	-3.4	-10.6	-17.3	-3.9	-2.2	-3.6	-0.8
**ANC4**	**Total Covid Period**	**-8.9**	**-11.6**	**-6.2**	**-10.3**	**-13.4**	**-7.2**	**-2.2**	**-2.9**	**-1.5**
Deliveries	Mar 2020	0.4	-2.8	3.7	0.5	-3.2	4.1	0.1	-0.8	1.1
Deliveries	Apr 2020	-3.2	-6.4	0.0	-3.6	-7.2	0.0	-0.9	-1.9	0.0
Deliveries	May 2020	-3.8	-7.0	-0.6	-4.3	-7.9	-0.7	-1.1	-2.0	-0.2
Deliveries	Jun 2020	-1.2	-4.4	2.0	-1.4	-5.0	2.3	-0.3	-1.3	0.6
Deliveries	Jul 2020	-1.2	-4.4	2.0	-1.3	-5.0	2.3	-0.3	-1.3	0.6
Deliveries	Aug 2020	-2.5	-5.7	0.7	-2.8	-6.4	0.8	-0.7	-1.7	0.2
Deliveries	Sep 2020	-2.6	-5.8	0.6	-2.9	-6.5	0.7	-0.7	-1.7	0.2
Deliveries	Oct 2020	-4.2	-7.4	-0.9	-4.7	-8.3	-1.1	-1.2	-2.1	-0.3
Deliveries	Nov 2020	-4.9	-8.1	-1.6	-5.5	-9.1	-1.9	-1.4	-2.3	-0.5
Deliveries	Dec 2020	-12.5	-15.7	-9.3	-14.0	-17.7	-10.4	-3.6	-4.6	-2.7
**Deliveries**	**Total Covid Period**	**-3.5**	**-5.1**	**-2.0**	**-4.0**	**-5.7**	**-2.2**	**-1.0**	**-1.5**	**-0.6**
Penta1	Mar 2020	2.1	-1.3	5.5	2.3	-1.5	6.0	0.7	-0.4	1.8
Penta1	Apr 2020	-3.1	-6.6	0.3	-3.4	-7.2	0.3	-1.0	-2.2	0.1
Penta1	May 2020	-10.6	-14.0	-7.2	-11.6	-15.4	-7.9	-3.5	-4.7	-2.4
Penta1	Jun 2020	7.8	4.4	11.2	8.6	4.8	12.3	2.6	1.5	3.7
Penta1	Jul 2020	-1.7	-5.1	1.7	-1.8	-5.6	1.9	-0.6	-1.7	0.6
Penta1	Aug 2020	-4.3	-7.7	-0.9	-4.7	-8.5	-1.0	-1.4	-2.6	-0.3
Penta1	Sep 2020	3.5	0.0	6.9	3.8	0.0	7.6	1.2	0.0	2.3
Penta1	Oct 2020	-4.4	-7.8	-1.0	-4.8	-8.6	-1.1	-1.5	-2.6	-0.3
Penta1	Nov 2020	-0.3	-3.7	3.1	-0.3	-4.1	3.4	-0.1	-1.2	1.0
Penta1	Dec 2020	-4.1	-7.5	-0.7	-4.5	-8.2	-0.8	-1.4	-2.5	-0.2
**Penta1**	**Total Covid Period**	**-1.5**	**-3.2**	**0.2**	**-1.7**	**-3.5**	**0.2**	**-0.5**	**-1.1**	**0.1**
Penta3	Mar 2020	1.1	-2.3	4.5	1.2	-2.5	4.9	0.4	-0.8	1.5
Penta3	Apr 2020	3.0	-0.4	6.4	3.3	-0.4	7.0	1.0	-0.1	2.1
Penta3	May 2020	-4.4	-7.8	-1.0	-4.8	-8.5	-1.1	-1.5	-2.6	-0.3
Penta3	Jun 2020	8.0	4.6	11.4	8.8	5.0	12.5	2.7	1.5	3.8
Penta3	Jul 2020	2.8	-0.6	6.2	3.1	-0.6	6.8	0.9	-0.2	2.1
Penta3	Aug 2020	1.5	-1.9	4.9	1.7	-2.1	5.4	0.5	-0.6	1.6
Penta3	Sep 2020	4.1	0.7	7.5	4.5	0.8	8.3	1.4	0.2	2.5
Penta3	Oct 2020	-0.4	-3.8	3.0	-0.4	-4.2	3.3	-0.1	-1.3	1.0
Penta3	Nov 2020	2.5	-0.8	5.9	2.8	-0.9	6.5	0.8	-0.3	2.0
Penta3	Dec 2020	0.4	-3.0	3.8	0.4	-3.3	4.2	0.1	-1.0	1.3
**Penta3**	**Total Covid Period**	**1.9**	**0.2**	**3.5**	**2.1**	**0.2**	**3.9**	**0.6**	**0.1**	**1.2**
C-section	Mar 2020	2.7	-2.5	7.8	3.3	-3.1	9.7	0.5	-0.5	1.4
C-section	Apr 2020	0.2	-5.0	5.3	0.2	-6.2	6.6	0.0	-0.9	1.0
C-section	May 2020	0.6	-4.5	5.7	0.7	-5.7	7.1	0.1	-0.8	1.1
C-section	Jun 2020	5.4	0.2	10.5	6.7	0.3	13.1	1.0	0.0	1.9
C-section	Jul 2020	4.2	-1.0	9.3	5.2	-1.2	11.6	0.8	-0.2	1.7
C-section	Aug 2020	-0.3	-5.5	4.8	-0.4	-6.8	6.0	-0.1	-1.0	0.9
C-section	Sep 2020	2.0	-3.1	7.2	2.5	-3.9	8.9	0.4	-0.6	1.3
C-section	Oct 2020	-0.3	-5.5	4.8	-0.4	-6.8	6.0	-0.1	-1.0	0.9
C-section	Nov 2020	-4.2	-9.3	1.0	-5.2	-11.6	1.2	-0.8	-1.7	0.2
C-section	Dec 2020	-8.0	-13.1	-2.9	-10.0	-16.4	-3.6	-1.5	-2.4	-0.5
**C-section**	**Total Covid Period**	**0.2**	**-2.3**	**2.7**	**0.3**	**-2.8**	**3.4**	**0.0**	**-0.4**	**0.5**
Measles vaccination	Mar 2020	15.2	9.5	20.9	16.7	10.4	22.9	5.0	3.2	6.9
Measles vaccination	Apr 2020	7.6	2.0	13.3	8.4	2.2	14.6	2.5	0.7	4.4
Measles vaccination	May 2020	2.2	-3.5	7.8	2.4	-3.8	8.6	0.7	-1.2	2.6
Measles vaccination	Jun 2020	16.8	11.1	22.5	18.4	12.2	24.7	5.6	3.7	7.4
Measles vaccination	Jul 2020	13.8	8.2	19.5	15.2	9.0	21.4	4.6	2.7	6.5
Measles vaccination	Aug 2020	-0.7	-6.3	5.0	-0.7	-6.9	5.4	-0.2	-2.1	1.7
Measles vaccination	Sep 2020	2.2	-3.4	7.9	2.4	-3.8	8.6	0.7	-1.1	2.6
Measles vaccination	Oct 2020	1.7	-3.9	7.4	1.9	-4.3	8.1	0.6	-1.3	2.4
Measles vaccination	Nov 2020	5.9	0.2	11.5	6.5	0.3	12.7	2.0	0.1	3.8
Measles vaccination	Dec 2020	5.7	0.1	11.4	6.3	0.1	12.5	1.9	0.0	3.8
**Measles vaccination**	**Total Covid Period**	**7.2**	**4.3**	**10.0**	**7.9**	**4.7**	**11.0**	**2.4**	**1.4**	**3.3**
OPD	Mar 2020	-2.6	-8.5	3.2	-2.8	-9.2	3.5	-1.0	-3.1	1.2
OPD	Apr 2020	-32.9	-38.8	-27.0	-35.6	-42.0	-29.2	-12.2	-14.4	-10.0
OPD	May 2020	-49.6	-55.6	-43.7	-53.6	-60.1	-47.2	-18.5	-20.7	-16.3
OPD	Jun 2020	-35.7	-41.6	-29.8	-38.6	-45.0	-32.2	-13.3	-15.5	-11.1
OPD	Jul 2020	-47.6	-53.5	-41.6	-51.4	-57.9	-45.0	-17.7	-19.9	-15.5
OPD	Aug 2020	-30.8	-36.7	-25.0	-33.4	-39.7	-27.0	-11.5	-13.6	-9.3
OPD	Sep 2020	-32.8	-38.7	-26.9	-35.5	-41.8	-29.1	-12.2	-14.4	-10.0
OPD	Oct 2020	-29.5	-35.4	-23.7	-31.9	-38.3	-25.6	-11.0	-13.1	-8.8
OPD	Nov 2020	-17.4	-23.2	-11.5	-18.8	-25.1	-12.5	-6.4	-8.6	-4.3
OPD	Dec 2020	-35.7	-41.6	-29.8	-38.6	-45.0	-32.2	-13.3	-15.4	-11.1
**OPD**	**Total Covid Period**	**-29.2**	**-31.9**	**-26.5**	**-31.4**	**-34.3**	**-28.5**	**-11.4**	**-12.5**	**-10.3**
IPD	Mar 2020	-13.8	-21.5	-6.1	-15.4	-23.9	-6.8	-4.2	-6.5	-1.8
IPD	Apr 2020	-34.1	-41.8	-26.4	-38.0	-46.5	-29.4	-10.3	-12.7	-8.0
IPD	May 2020	-32.7	-40.4	-25.1	-36.5	-45.0	-27.9	-9.9	-12.3	-7.6
IPD	Jun 2020	-28.4	-36.1	-20.7	-31.6	-40.2	-23.1	-8.6	-10.9	-6.3
IPD	Jul 2020	-33.5	-41.2	-25.9	-37.3	-45.9	-28.8	-10.2	-12.5	-7.8
IPD	Aug 2020	-24.2	-31.9	-16.5	-27.0	-35.5	-18.4	-7.3	-9.7	-5.0
IPD	Sep 2020	-23.1	-30.7	-15.4	-25.7	-34.2	-17.2	-7.0	-9.3	-4.7
IPD	Oct 2020	-20.2	-27.9	-12.5	-22.5	-31.0	-13.9	-6.1	-8.4	-3.8
IPD	Nov 2020	-25.0	-32.6	-17.3	-27.8	-36.4	-19.3	-7.6	-9.9	-5.2
IPD	Dec 2020	-29.7	-37.3	-22.0	-33.1	-41.6	-24.5	-9.0	-11.3	-6.7
**IPD**	**Total Covid Period**	**-24.8**	**-28.2**	**-21.5**	**-27.5**	**-31.1**	**-23.8**	**-7.9**	**-9.0**	**-6.8**

		National			Rural			Urban		
**Indicator**	**Month/Period**	**%change**	**Lower bound**	**Upper bound**	**%change**	**Lower bound**	**Upper bound**	**%change**	**Lower bound**	**Upper bound**
ANC1	Mar 2020	-14.7	-28.1	-1.2	-21.3	-43.3	0.8	-4.9	-9.3	-0.5
ANC1	Apr 2020	-19.8	-34.3	-5.3	-28.7	-53.8	-3.6	-6.6	-11.2	-1.9
ANC1	May 2020	-8.9	-21.5	3.7	-12.9	-32.3	6.4	-2.9	-7.1	1.2
ANC1	Jun 2020	9.2	-3.6	22.0	13.3	-6.4	33.1	3.0	-1.1	7.2
ANC1	Jul 2020	-6.7	-19.2	5.7	-9.8	-28.4	8.8	-2.2	-6.3	1.9
ANC1	Aug 2020	-1.2	-13.4	10.9	-1.8	-19.4	15.9	-0.4	-4.4	3.6
ANC1	Sep 2020	-2.8	-15.0	9.3	-4.1	-21.9	13.7	-0.9	-5.0	3.1
ANC1	Oct 2020	-17.0	-30.9	-3.1	-24.6	-48.0	-1.2	-5.6	-10.1	-1.1
ANC1	Nov 2020	-1.9	-14.1	10.2	-2.8	-20.5	14.9	-0.6	-4.7	3.4
ANC1	Dec 2020	9.3	-3.5	22.1	13.6	-6.2	33.3	3.1	-1.1	7.3
**ANC1**	**Total Covid Period**	-5.4	-11.6	0.8	-7.8	-17.4	1.8	-1.8	-3.9	0.3
ANC4	Mar 2020	-7.9	-21.7	5.9	-10.1	-28.1	7.9	-3.3	-9.0	2.5
ANC4	Apr 2020	-20.8	-36.3	-5.2	-26.5	-48.4	-4.6	-8.6	-15.3	-2.0
ANC4	May 2020	-10.8	-24.9	3.3	-13.8	-32.4	4.9	-4.5	-10.4	1.4
ANC4	Jun 2020	7.6	-6.3	21.6	9.8	-8.4	27.9	3.2	-2.6	8.9
ANC4	Jul 2020	5.7	-8.1	19.5	7.3	-10.5	25.1	2.3	-3.4	8.1
ANC4	Aug 2020	-3.4	-17.0	10.2	-4.4	-21.8	13.1	-1.4	-7.1	4.2
ANC4	Sep 2020	-5.5	-19.2	8.2	-7.1	-24.7	10.6	-2.3	-8.0	3.4
ANC4	Oct 2020	-21.6	-37.3	-5.9	-27.5	-49.8	-5.3	-9.0	-15.7	-2.3
ANC4	Nov 2020	-5.1	-18.8	8.6	-6.5	-24.1	11.1	-2.1	-7.8	3.6
ANC4	Dec 2020	-1.4	-15.0	12.1	-1.8	-19.2	15.5	-0.6	-6.2	5.0
ANC4	Total Covid Period	-6.2	-13.0	0.6	-7.9	-17.0	1.1	-2.6	-5.5	0.3
Deliveries	Mar 2020	1.9	-7.0	10.7	2.1	-8.0	12.2	1.0	-3.9	5.9
Deliveries	Apr 2020	-9.0	-18.0	0.0	-10.3	-20.7	0.2	-5.0	-10.1	0.2
Deliveries	May 2020	-4.1	-13.0	4.7	-4.7	-14.9	5.4	-2.3	-7.2	2.7
Deliveries	Jun 2020	3.2	-5.7	12.1	3.7	-6.5	13.9	1.8	-3.1	6.7
Deliveries	Jul 2020	-4.9	-13.8	4.0	-5.6	-15.8	4.6	-2.7	-7.7	2.2
Deliveries	Aug 2020	-2.4	-11.2	6.5	-2.7	-12.8	7.4	-1.3	-6.2	3.6
Deliveries	Sep 2020	-3.7	-12.5	5.2	-4.2	-14.3	6.0	-2.0	-7.0	2.9
Deliveries	Oct 2020	-9.6	-18.7	-0.6	-11.0	-21.4	-0.5	-5.3	-10.5	-0.1
Deliveries	Nov 2020	-7.3	-16.3	1.6	-8.4	-18.7	2.0	-4.0	-9.1	1.0
Deliveries	Dec 2020	-13.0	-22.2	-3.7	-14.8	-25.6	-4.0	-7.2	-12.6	-1.8
Deliveries	Total Covid Period	-4.8	-9.1	-0.5	-5.5	-10.5	-0.5	-2.7	-5.2	-0.2
Penta1	Mar 2020	-3.6	-14.9	7.7	-5.1	-21.3	11.1	-1.2	-5.1	2.6
Penta1	Apr 2020	-26.3	-41.0	-11.5	-37.3	-63.5	-11.0	-9.0	-13.9	-4.1
Penta1	May 2020	6.2	-5.2	17.7	8.9	-7.9	25.7	2.1	-1.8	6.0
Penta1	Jun 2020	4.8	-6.6	16.2	6.8	-9.7	23.3	1.6	-2.2	5.5
Penta1	Jul 2020	2.6	-8.7	13.9	3.7	-12.4	19.8	0.9	-3.0	4.7
Penta1	Aug 2020	5.4	-6.0	16.8	7.7	-8.9	24.3	1.8	-2.0	5.7
Penta1	Sep 2020	28.1	12.5	43.6	40.1	11.7	68.5	9.5	4.5	14.5
Penta1	Oct 2020	-1.4	-12.6	9.8	-2.0	-18.0	14.0	-0.5	-4.3	3.3
Penta1	Nov 2020	20.9	7.1	34.7	29.8	6.2	53.4	7.1	2.6	11.6
Penta1	Dec 2020	-4.4	-15.7	6.9	-6.3	-22.6	10.0	-1.5	-5.4	2.3
Penta1	Total Covid Period	3.2	-2.7	9.1	4.6	-4.2	13.3	1.1	-0.9	3.1
Penta3	Mar 2020	-2.0	-13.6	9.6	-2.8	-19.3	13.7	-0.7	-4.6	3.3
Penta3	Apr 2020	-29.4	-45.4	-13.4	-41.7	-70.7	-12.7	-10.1	-15.4	-4.8
Penta3	May 2020	3.4	-8.3	15.1	4.8	-11.9	21.6	1.2	-2.8	5.1
Penta3	Jun 2020	-2.6	-14.2	9.0	-3.7	-20.2	12.9	-0.9	-4.8	3.1
Penta3	Jul 2020	1.8	-9.8	13.4	2.5	-14.0	19.1	0.6	-3.3	4.6
Penta3	Aug 2020	6.9	-5.0	18.8	9.8	-7.7	27.3	2.3	-1.7	6.4
Penta3	Sep 2020	24.0	9.0	38.9	34.2	7.9	60.4	8.1	3.3	13.0
Penta3	Oct 2020	6.5	-5.3	18.4	9.3	-8.1	26.7	2.2	-1.8	6.3
Penta3	Nov 2020	27.8	11.8	43.7	39.6	10.7	68.5	9.4	4.3	14.6
Penta3	Dec 2020	0.0	-11.6	11.5	-0.1	-16.5	16.4	0.0	-4.0	3.9
Penta3	Total Covid Period	3.6	-2.6	9.8	5.1	-4.0	14.3	1.2	-0.9	3.3
C-section	Mar 2020	-22.7	-50.8	5.5	-37.5	-91.4	16.5	-6.3	-14.0	1.3
C-section	Apr 2020	-1.4	-27.5	24.7	-2.3	-45.7	41.1	-0.4	-7.7	6.9
C-section	May 2020	18.6	-9.4	46.7	31.1	-21.3	83.4	5.2	-2.4	12.7
C-section	Jun 2020	1.0	-25.1	27.2	1.7	-41.7	45.2	0.3	-7.0	7.6
C-section	Jul 2020	1.7	-24.5	27.9	2.8	-40.7	46.4	0.5	-6.8	7.8
C-section	Aug 2020	-9.9	-36.4	16.5	-16.5	-61.9	29.0	-2.8	-10.1	4.6
C-section	Sep 2020	-12.3	-39.0	14.3	-20.5	-67.1	26.2	-3.5	-10.8	3.9
C-section	Oct 2020	-11.0	-37.6	15.5	-18.3	-64.2	27.7	-3.1	-10.4	4.3
C-section	Nov 2020	-5.4	-31.6	20.8	-8.9	-52.8	35.0	-1.5	-8.8	5.8
C-section	Dec 2020	1.0	-25.2	27.1	1.6	-41.8	45.1	0.3	-7.0	7.6
C-section	Total Covid Period	-4.0	-16.5	8.4	-6.6	-27.6	14.3	-1.1	-4.6	2.4
Measles vaccination	Mar 2020	-14.1	-28.6	0.4	-21.1	-45.7	3.5	-4.5	-8.9	0.0
Measles vaccination	Apr 2020	-44.0	-67.5	-20.6	-65.6	-115.7	-15.4	-14.0	-20.7	-7.4
Measles vaccination	May 2020	-1.4	-14.5	11.7	-2.1	-21.8	17.6	-0.4	-4.6	3.7
Measles vaccination	Jun 2020	-5.3	-18.6	8.0	-7.9	-28.3	12.5	-1.7	-5.9	2.5
Measles vaccination	Jul 2020	-4.5	-17.7	8.8	-6.7	-26.9	13.4	-1.4	-5.6	2.8
Measles vaccination	Aug 2020	-3.6	-16.8	9.6	-5.4	-25.4	14.5	-1.1	-5.3	3.0
Measles vaccination	Sep 2020	2.5	-10.7	15.7	3.7	-16.1	23.6	0.8	-3.4	4.9
Measles vaccination	Oct 2020	-2.1	-15.2	11.0	-3.2	-22.9	16.6	-0.7	-4.8	3.5
Measles vaccination	Nov 2020	23.3	6.3	40.3	35.1	2.6	67.5	7.3	2.4	12.3
Measles vaccination	Dec 2020	19.6	3.6	35.6	29.4	0.2	58.7	6.2	1.4	10.9
Measles vaccination	Total Covid Period	-3.0	-9.8	3.7	-4.5	-14.9	5.8	-1.0	-3.1	1.2
OPD	Mar 2020	-6.3	-17.5	4.9	-9.5	-27.2	8.2	-2.0	-5.5	1.5
OPD	Apr 2020	-28.4	-44.8	-12.0	-42.6	-76.5	-8.7	-9.0	-13.7	-4.2
OPD	May 2020	-21.6	-35.9	-7.3	-32.4	-60.4	-4.5	-6.8	-11.1	-2.6
OPD	Jun 2020	7.1	-4.2	18.5	10.8	-7.4	29.0	2.2	-1.3	5.8
OPD	Jul 2020	-6.2	-17.5	5.0	-9.4	-27.0	8.2	-2.0	-5.5	1.5
OPD	Aug 2020	-2.6	-13.6	8.3	-4.0	-20.6	12.7	-0.8	-4.3	2.6
OPD	Sep 2020	-0.4	-11.3	10.5	-0.5	-17.0	15.9	-0.1	-3.5	3.3
OPD	Oct 2020	-17.9	-31.3	-4.6	-26.9	-51.9	-2.0	-5.6	-9.7	-1.6
OPD	Nov 2020	-5.3	-16.4	5.8	-8.0	-25.3	9.3	-1.7	-5.1	1.8
OPD	Dec 2020	9.4	-2.3	21.1	14.2	-5.2	33.6	3.0	-0.6	6.6
OPD	Total Covid Period	-7.1	-13.2	-1.0	-10.6	-21.3	0.0	-2.3	-4.2	-0.4

MALI

		National			Rural			Urban			Mixed		
**Indicator**	**Month/Period**	**%change**	**Lower bound**	**Upper bound**	**%change**	**Lower bound**	**Upper bound**	**%change**	**Lower bound**	**Upper bound**	**%change**	**Lower bound**	**Upper bound**
ANC1	Mar 2020	-21.0	-25.4	-16.6	-26.8	-32.6	-21.0	-13.0	-15.8	-10.2	-18.0	-22.6	-13.4
ANC1	Apr 2020	10.3	5.9	14.7	13.1	7.5	18.8	6.3	3.6	9.1	8.8	4.8	12.8
ANC1	May 2020	2.9	-1.5	7.2	3.7	-1.9	9.3	1.8	-0.9	4.5	2.5	-1.3	6.2
ANC1	Jun 2020	13.7	9.2	18.1	17.5	11.8	23.2	8.4	5.7	11.2	11.7	7.6	15.9
ANC1	Jul 2020	-17.6	-22.0	-13.2	-22.5	-28.2	-16.8	-10.9	-13.6	-8.2	-15.1	-19.5	-10.8
ANC1	Aug 2020	-8.0	-12.4	-3.7	-10.3	-15.8	-4.7	-5.0	-7.7	-2.3	-6.9	-10.8	-3.0
ANC1	Sep 2020	-1.6	-5.9	2.8	-2.0	-7.6	3.5	-1.0	-3.7	1.7	-1.4	-5.1	2.4
ANC1	Oct 2020	-4.3	-8.6	0.1	-5.4	-11.0	0.1	-2.6	-5.3	0.1	-3.6	-7.4	0.1
ANC1	Nov 2020	-4.1	-8.4	0.3	-5.2	-10.8	0.3	-2.5	-5.2	0.2	-3.5	-7.3	0.3
ANC1	Dec 2020	-0.7	-5.1	3.6	-0.9	-6.5	4.6	-0.5	-3.1	2.2	-0.6	-4.4	3.1
**ANC1**	Total Covid Period	-3.0	-4.9	-1.1	-3.9	-6.3	-1.4	-1.9	-3.1	-0.7	-2.6	-4.3	-0.9
ANC4	Mar 2020	-25.6	-33.1	-18.0	-33.6	-44.3	-22.8	-15.1	-19.6	-10.5	-24.7	-37.0	-12.4
ANC4	Apr 2020	-13.5	-20.7	-6.3	-17.8	-27.5	-8.1	-8.0	-12.2	-3.7	-13.1	-21.8	-4.4
ANC4	May 2020	-11.8	-18.9	-4.6	-15.4	-25.0	-5.8	-6.9	-11.2	-2.7	-11.3	-19.6	-3.1
ANC4	Jun 2020	-12.1	-19.3	-4.9	-15.9	-25.5	-6.3	-7.1	-11.4	-2.9	-11.7	-20.1	-3.3
ANC4	Jul 2020	-22.1	-29.6	-14.7	-29.0	-39.4	-18.7	-13.1	-17.5	-8.6	-21.4	-32.6	-10.2
ANC4	Aug 2020	-16.1	-23.3	-8.8	-21.1	-31.0	-11.2	-9.5	-13.8	-5.2	-15.5	-24.9	-6.1
ANC4	Sep 2020	-15.5	-22.8	-8.3	-20.4	-30.2	-10.5	-9.1	-13.5	-4.8	-15.0	-24.2	-5.8
ANC4	Oct 2020	-17.7	-25.0	-10.4	-23.3	-33.3	-13.3	-10.4	-14.8	-6.1	-17.1	-27.0	-7.3
ANC4	Nov 2020	-16.5	-23.7	-9.2	-21.6	-31.5	-11.7	-9.7	-14.0	-5.4	-15.9	-25.4	-6.4
ANC4	Dec 2020	-18.2	-25.5	-10.9	-23.9	-33.9	-13.8	-10.7	-15.1	-6.4	-17.6	-27.6	-7.6
ANC4	Total Covid Period	-16.4	-19.7	-13.0	-21.2	-26.2	-16.2	-9.8	-11.9	-7.7	-15.8	-22.8	-8.9
Deliveries	Mar 2020	-4.0	-9.0	1.0	-5.5	-12.4	1.4	-2.2	-5.0	0.6	-3.7	-8.5	1.0
Deliveries	Apr 2020	-2.8	-7.9	2.2	-3.9	-10.8	3.0	-1.6	-4.3	1.2	-2.7	-7.4	2.1
Deliveries	May 2020	-6.1	-11.1	-1.1	-8.4	-15.4	-1.5	-3.4	-6.1	-0.6	-5.7	-10.6	-0.8
Deliveries	Jun 2020	-8.9	-14.0	-3.9	-12.3	-19.3	-5.3	-4.9	-7.7	-2.2	-8.4	-13.5	-3.3
Deliveries	Jul 2020	-6.8	-11.8	-1.8	-9.4	-16.3	-2.4	-3.7	-6.5	-1.0	-6.3	-11.3	-1.4
Deliveries	Aug 2020	-6.2	-11.2	-1.1	-8.5	-15.5	-1.5	-3.4	-6.2	-0.6	-5.8	-10.7	-0.9
Deliveries	Sep 2020	1.1	-3.9	6.2	1.6	-5.4	8.5	0.6	-2.1	3.4	1.1	-3.6	5.8
Deliveries	Oct 2020	1.5	-3.5	6.6	2.1	-4.8	9.1	0.8	-1.9	3.6	1.4	-3.3	6.2
Deliveries	Nov 2020	-3.1	-8.2	1.9	-4.3	-11.3	2.6	-1.7	-4.5	1.0	-2.9	-7.7	1.8
Deliveries	Dec 2020	1.3	-3.7	6.3	1.8	-5.1	8.8	0.7	-2.0	3.5	1.2	-3.5	5.9
Deliveries	Total Covid Period	-3.4	-5.5	-1.2	-4.7	-7.6	-1.7	-1.9	-3.1	-0.7	-3.2	-5.3	-1.0
Penta1	Mar 2020	-29.5	-34.3	-24.8	-37.5	-43.7	-31.3	-18.2	-21.2	-15.2	-26.3	-31.3	-21.3
Penta1	Apr 2020	-8.5	-13.2	-3.8	-10.8	-16.8	-4.8	-5.2	-8.1	-2.3	-7.5	-11.8	-3.3
Penta1	May 2020	-3.8	-8.5	0.9	-4.8	-10.9	1.2	-2.3	-5.3	0.6	-3.4	-7.6	0.8
Penta1	Jun 2020	-3.2	-7.9	1.5	-4.1	-10.1	2.0	-2.0	-4.9	0.9	-2.8	-7.1	1.4
Penta1	Jul 2020	-18.7	-23.4	-13.9	-23.7	-29.8	-17.6	-11.5	-14.4	-8.6	-16.6	-21.2	-12.1
Penta1	Aug 2020	-24.4	-29.2	-19.7	-31.0	-37.1	-24.8	-15.0	-18.0	-12.1	-21.7	-26.5	-17.0
Penta1	Sep 2020	-10.3	-15.1	-5.6	-13.1	-19.1	-7.1	-6.4	-9.3	-3.5	-9.2	-13.5	-4.9
Penta1	Oct 2020	-11.8	-16.5	-7.1	-15.0	-21.0	-9.0	-7.3	-10.2	-4.3	-10.5	-14.8	-6.2
Penta1	Nov 2020	-5.0	-9.7	-0.2	-6.3	-12.3	-0.3	-3.1	-6.0	-0.1	-4.4	-8.6	-0.2
Penta1	Dec 2020	-11.9	-16.6	-7.2	-15.1	-21.1	-9.1	-7.3	-10.2	-4.4	-10.6	-14.9	-6.2
Penta1	Total Covid Period	-12.4	-14.4	-10.4	-15.7	-18.2	-13.1	-7.7	-9.0	-6.5	-11.1	-13.2	-9.0
Penta3	Mar 2020	-28.3	-33.4	-23.3	-35.7	-42.2	-29.2	-17.7	-20.9	-14.6	-24.7	-29.5	-19.8
Penta3	Apr 2020	-11.0	-16.0	-6.0	-13.9	-20.2	-7.6	-6.9	-10.0	-3.8	-9.6	-14.0	-5.2
Penta3	May 2020	-5.1	-10.1	-0.1	-6.4	-12.7	-0.1	-3.2	-6.3	-0.1	-4.4	-8.8	-0.1
Penta3	Jun 2020	-9.1	-14.0	-4.1	-11.4	-17.7	-5.1	-5.7	-8.8	-2.5	-7.9	-12.3	-3.5
Penta3	Jul 2020	-20.3	-25.4	-15.3	-25.7	-32.0	-19.3	-12.7	-15.8	-9.6	-17.7	-22.3	-13.1
Penta3	Aug 2020	-27.3	-32.3	-22.2	-34.4	-40.8	-27.9	-17.0	-20.2	-13.9	-23.8	-28.6	-18.9
Penta3	Sep 2020	-14.6	-19.6	-9.6	-18.4	-24.7	-12.1	-9.1	-12.2	-6.0	-12.7	-17.2	-8.2
Penta3	Oct 2020	-14.5	-19.5	-9.5	-18.3	-24.7	-12.0	-9.1	-12.2	-5.9	-12.6	-17.1	-8.2
Penta3	Nov 2020	-9.9	-14.9	-4.9	-12.5	-18.8	-6.2	-6.2	-9.3	-3.1	-8.6	-13.0	-4.2
Penta3	Dec 2020	-16.7	-21.7	-11.7	-21.1	-27.4	-14.7	-10.4	-13.6	-7.3	-14.5	-19.1	-10.0
Penta3	Total Covid Period	-15.2	-17.3	-13.1	-19.1	-21.8	-16.4	-9.6	-11.0	-8.3	-13.3	-15.5	-11.2
C-section	Mar 2020	-12.2	-22.9	-1.4	-18.3	-34.9	-1.7	-6.5	-12.2	-0.8	-20.1	-44.3	4.2
C-section	Apr 2020	-3.5	-14.2	7.1	-5.3	-21.4	10.7	-1.9	-7.6	3.8	-5.9	-24.1	12.4
C-section	May 2020	-5.7	-16.2	4.8	-8.6	-24.5	7.4	-3.0	-8.6	2.6	-9.4	-28.5	9.7
C-section	Jun 2020	-12.6	-23.4	-1.9	-19.0	-35.7	-2.4	-6.7	-12.4	-1.0	-20.8	-45.6	3.9
C-section	Jul 2020	-12.7	-23.5	-1.9	-19.1	-35.8	-2.4	-6.8	-12.5	-1.0	-20.9	-45.7	3.9
C-section	Aug 2020	-1.7	-12.3	9.0	-2.5	-18.6	13.6	-0.9	-6.5	4.8	-2.8	-20.5	15.0
C-section	Sep 2020	-2.5	-13.2	8.1	-3.8	-19.9	12.3	-1.3	-7.0	4.3	-4.2	-22.2	13.8
C-section	Oct 2020	6.2	-4.5	16.9	9.4	-6.9	25.7	3.3	-2.4	9.0	10.3	-9.4	29.9
C-section	Nov 2020	-3.6	-14.2	7.0	-5.4	-21.4	10.6	-1.9	-7.5	3.7	-5.9	-24.1	12.3
C-section	Dec 2020	-3.3	-14.0	7.4	-5.0	-21.1	11.1	-1.8	-7.4	3.9	-5.5	-23.7	12.8
C-section	Total Covid Period	-5.1	-9.7	-0.6	-7.7	-14.6	-0.7	-2.7	-5.2	-0.3	-8.4	-18.5	1.7
Measles vaccination	Mar 2020	-31.3	-36.4	-26.1	-39.2	-45.8	-32.6	-19.7	-22.9	-16.4	-27.7	-32.8	-22.5
Measles vaccination	Apr 2020	-8.2	-13.3	-3.2	-10.3	-16.7	-4.0	-5.2	-8.3	-2.0	-7.3	-11.8	-2.8
Measles vaccination	May 2020	-9.7	-14.8	-4.7	-12.2	-18.6	-5.9	-6.1	-9.3	-2.9	-8.6	-13.1	-4.1
Measles vaccination	Jun 2020	-4.5	-9.5	0.6	-5.6	-12.0	0.8	-2.8	-6.0	0.4	-3.9	-8.4	0.6
Measles vaccination	Jul 2020	-21.8	-26.9	-16.7	-27.3	-33.8	-20.9	-13.7	-16.9	-10.5	-19.3	-24.1	-14.5
Measles vaccination	Aug 2020	-29.0	-34.1	-23.9	-36.4	-42.9	-29.8	-18.2	-21.4	-15.0	-25.6	-30.7	-20.6
Measles vaccination	Sep 2020	-15.1	-20.2	-10.0	-18.9	-25.3	-12.5	-9.5	-12.6	-6.3	-13.3	-18.0	-8.7
Measles vaccination	Oct 2020	-16.4	-21.4	-11.3	-20.5	-26.9	-14.1	-10.3	-13.5	-7.1	-14.5	-19.1	-9.8
Measles vaccination	Nov 2020	-14.0	-19.1	-9.0	-17.6	-24.0	-11.2	-8.8	-12.0	-5.6	-12.4	-17.0	-7.8
Measles vaccination	Dec 2020	-19.1	-24.2	-14.0	-24.0	-30.4	-17.5	-12.0	-15.2	-8.8	-16.9	-21.6	-12.2
Measles vaccination	Total Covid Period	-16.4	-18.5	-14.2	-20.4	-23.1	-17.6	-10.4	-11.8	-9.0	-14.6	-16.8	-12.3
OPD	Mar 2020	-17.7	-26.3	-9.0	-24.6	-36.9	-12.3	-9.6	-14.3	-4.9	-15.7	-24.5	-6.9
OPD	Apr 2020	4.1	-4.5	12.7	5.8	-6.3	17.8	2.2	-2.4	6.9	3.7	-4.0	11.4
OPD	May 2020	2.3	-6.3	10.9	3.3	-8.7	15.3	1.3	-3.4	5.9	2.1	-5.6	9.7
OPD	Jun 2020	-9.9	-18.5	-1.3	-13.8	-25.8	-1.7	-5.4	-10.0	-0.7	-8.8	-16.8	-0.8
OPD	Jul 2020	-11.1	-19.7	-2.5	-15.5	-27.6	-3.4	-6.0	-10.7	-1.4	-9.9	-18.0	-1.8
OPD	Aug 2020	-8.8	-17.4	-0.2	-12.3	-24.3	-0.2	-4.8	-9.4	-0.1	-7.8	-15.7	0.1
OPD	Sep 2020	-3.5	-12.1	5.1	-4.9	-16.9	7.1	-1.9	-6.6	2.8	-3.1	-10.8	4.6
OPD	Oct 2020	-8.2	-16.8	0.4	-11.4	-23.5	0.6	-4.5	-9.1	0.2	-7.3	-15.2	0.6
OPD	Nov 2020	-4.8	-13.4	3.8	-6.7	-18.7	5.3	-2.6	-7.3	2.1	-4.2	-11.9	3.5
OPD	Dec 2020	-4.9	-13.4	3.7	-6.8	-18.8	5.2	-2.6	-7.3	2.0	-4.3	-12.0	3.4
OPD	Total Covid Period	-6.2	-9.8	-2.5	-8.6	-13.6	-3.5	-3.4	-5.4	-1.4	-5.5	-9.0	-1.9
IPD	Mar 2020	-30.0	-54.8	-5.1	-32.0	-58.8	-5.2	-24.0	-44.0	-3.9	-38.4	-77.3	0.4
IPD	Apr 2020	11.2	-13.5	36.0	12.0	-14.5	38.5	9.0	-10.8	28.8	14.4	-18.6	47.4
IPD	May 2020	3.6	-21.0	28.2	3.8	-22.5	30.2	2.9	-16.8	22.5	4.6	-27.1	36.4
IPD	Jun 2020	-26.0	-50.7	-1.2	-27.7	-54.3	-1.1	-20.8	-40.7	-0.8	-33.3	-70.5	3.9
IPD	Jul 2020	-14.2	-38.7	10.4	-15.1	-41.4	11.2	-11.3	-31.0	8.4	-18.2	-51.5	15.1
IPD	Aug 2020	-25.3	-50.0	-0.5	-27.0	-53.6	-0.4	-20.2	-40.1	-0.3	-32.4	-69.3	4.5
IPD	Sep 2020	-2.1	-26.7	22.5	-2.3	-28.5	24.0	-1.7	-21.3	17.9	-2.7	-34.3	28.9
IPD	Oct 2020	11.2	-13.6	36.0	12.0	-14.6	38.5	8.9	-10.9	28.7	14.4	-18.6	47.4
IPD	Nov 2020	-0.4	-25.0	24.2	-0.4	-26.7	25.9	-0.3	-19.9	19.3	-0.5	-32.1	31.1
IPD	Dec 2020	-10.5	-35.0	14.1	-11.2	-37.4	15.1	-8.4	-28.0	11.3	-13.4	-45.9	19.1
IPD	Total Covid Period	-8.2	-18.5	2.1	-8.7	-19.7	2.3	-6.5	-14.9	1.8	-10.4	-24.8	4.0

Amouzou A, *et al. BMJ Global Health* 2022; 7:e008069. doi: 10.1136/bmjgh-2021-008069

NIGER

		National			Rural			Urban		
**Indicator**	**Month/Period**	**%change**	**Lower bound**	**Upper bound**	**%change**	**Lower bound**	**Upper bound**	**%change**	**Lower bound**	**Upper bound**
ANC1	Mar 2020	-14.2	-19.4	-9.0	-25.9	-37.8	-14.0	-11.4	-15.6	-7.3
ANC1	Apr 2020	9.7	4.6	14.8	17.8	7.1	28.5	7.8	3.7	11.9
ANC1	May 2020	8.3	3.2	13.4	15.1	4.8	25.4	6.7	2.6	10.8
ANC1	Jun 2020	28.8	23.0	34.7	53.0	34.3	71.6	23.2	18.5	27.9
ANC1	Jul 2020	-11.2	-16.3	-6.1	-20.4	-31.4	-9.5	-9.0	-13.1	-4.9
ANC1	Aug 2020	-4.1	-9.1	0.9	-7.5	-16.9	1.9	-3.3	-7.3	0.8
ANC1	Sep 2020	0.5	-4.5	5.5	1.0	-8.2	10.1	0.4	-3.6	4.5
ANC1	Oct 2020	-6.9	-12.0	-1.9	-12.7	-22.5	-2.8	-5.6	-9.6	-1.5
ANC1	Nov 2020	-5.4	-10.4	-0.3	-9.8	-19.4	-0.2	-4.3	-8.4	-0.3
ANC1	Dec 2020	-4.9	-10.0	0.1	-9.0	-18.5	0.5	-4.0	-8.0	0.1
**ANC1**	Total Covid Period	0.0	-2.2	2.2	0.1	-4.0	4.1	0.0	-1.8	1.8
ANC4	Mar 2020	-5.8	-11.8	0.3	-10.5	-21.8	0.9	-4.6	-9.5	0.2
ANC4	Apr 2020	-1.0	-6.9	5.0	-1.7	-12.6	9.2	-0.8	-5.6	4.1
ANC4	May 2020	18.7	12.4	25.1	34.2	18.6	49.8	15.1	10.0	20.2
ANC4	Jun 2020	7.8	1.7	13.9	14.3	2.4	26.1	6.3	1.4	11.2
ANC4	Jul 2020	-5.3	-11.3	0.7	-9.7	-21.0	1.6	-4.3	-9.1	0.6
ANC4	Aug 2020	3.1	-2.9	9.1	5.7	-5.4	16.8	2.5	-2.3	7.4
ANC4	Sep 2020	4.2	-1.8	10.2	7.6	-3.6	18.8	3.4	-1.5	8.2
ANC4	Oct 2020	-1.7	-7.7	4.3	-3.1	-14.0	7.8	-1.4	-6.2	3.5
ANC4	Nov 2020	-7.5	-13.5	-1.5	-13.7	-25.4	-2.0	-6.1	-10.9	-1.2
ANC4	Dec 2020	-7.1	-13.1	-1.1	-12.9	-24.5	-1.3	-5.7	-10.6	-0.9
ANC4	Total Covid Period	0.5	-2.1	3.1	1.0	-3.8	5.7	0.4	-1.7	2.5
Deliveries	Mar 2020	0.3	-5.4	6.0	0.6	-11.2	12.3	0.2	-4.3	4.7
Deliveries	Apr 2020	-10.2	-16.0	-4.5	-21.0	-34.7	-7.4	-8.0	-12.6	-3.5
Deliveries	May 2020	5.9	0.1	11.6	12.1	-0.4	24.6	4.6	0.1	9.1
Deliveries	Jun 2020	-9.4	-15.2	-3.7	-19.4	-32.7	-6.0	-7.4	-11.9	-2.9
Deliveries	Jul 2020	-17.6	-23.5	-11.6	-36.1	-52.8	-19.4	-13.8	-18.4	-9.2
Deliveries	Aug 2020	2.0	-3.7	7.7	4.0	-7.8	15.9	1.5	-2.9	6.0
Deliveries	Sep 2020	-4.9	-10.7	0.8	-10.2	-22.4	2.0	-3.9	-8.4	0.6
Deliveries	Oct 2020	-0.9	-6.6	4.8	-1.9	-13.7	9.8	-0.7	-5.2	3.7
Deliveries	Nov 2020	-10.9	-16.7	-5.2	-22.5	-36.4	-8.6	-8.6	-13.1	-4.1
Deliveries	Dec 2020	-11.4	-17.2	-5.7	-23.5	-37.6	-9.5	-9.0	-13.5	-4.5
Deliveries	Total Covid Period	-5.7	-8.2	-3.2	-11.6	-17.7	-5.4	-4.5	-6.4	-2.5
Penta1	Mar 2020	-3.8	-7.8	0.3	-7.3	-15.6	1.0	-3.0	-6.2	0.2
Penta1	Apr 2020	1.2	-2.8	5.3	2.3	-5.6	10.3	1.0	-2.3	4.2
Penta1	May 2020	4.9	0.8	9.0	9.5	0.9	18.1	3.9	0.6	7.1
Penta1	Jun 2020	9.2	5.0	13.4	17.8	7.6	28.1	7.3	4.0	10.6
Penta1	Jul 2020	-4.6	-8.6	-0.5	-8.8	-17.3	-0.4	-3.6	-6.9	-0.4
Penta1	Aug 2020	-3.1	-7.2	0.9	-6.0	-14.2	2.1	-2.5	-5.7	0.8
Penta1	Sep 2020	-0.5	-4.6	3.5	-1.0	-8.9	6.9	-0.4	-3.6	2.8
Penta1	Oct 2020	-1.7	-5.8	2.3	-3.3	-11.3	4.6	-1.4	-4.6	1.9
Penta1	Nov 2020	-2.7	-6.8	1.3	-5.3	-13.4	2.7	-2.2	-5.4	1.0
Penta1	Dec 2020	-1.8	-5.8	2.3	-3.4	-11.4	4.5	-1.4	-4.6	1.8
Penta1	Total Covid Period	-0.3	-2.0	1.4	-0.6	-4.0	2.8	-0.2	-1.6	1.1
Penta3	Mar 2020	-3.6	-7.8	0.7	-7.0	-15.7	1.6	-2.8	-6.2	0.5
Penta3	Apr 2020	1.3	-2.9	5.5	2.5	-5.8	10.9	1.0	-2.3	4.4
Penta3	May 2020	4.5	0.3	8.8	8.9	0.0	17.9	3.6	0.2	7.0
Penta3	Jun 2020	2.9	-1.3	7.2	5.8	-2.8	14.4	2.3	-1.0	5.7
Penta3	Jul 2020	-3.5	-7.8	0.7	-7.0	-15.6	1.7	-2.8	-6.2	0.5
Penta3	Aug 2020	-3.3	-7.6	0.9	-6.6	-15.2	2.1	-2.6	-6.0	0.7
Penta3	Sep 2020	-2.1	-6.3	2.1	-4.1	-12.6	4.3	-1.7	-5.0	1.7
Penta3	Oct 2020	-1.1	-5.3	3.1	-2.2	-10.6	6.1	-0.9	-4.2	2.4
Penta3	Nov 2020	-3.7	-7.9	0.6	-7.2	-15.9	1.5	-2.9	-6.3	0.4
Penta3	Dec 2020	-3.2	-7.5	1.0	-6.4	-15.0	2.2	-2.6	-5.9	0.8
Penta3	Total Covid Period	-1.2	-3.0	0.6	-2.3	-5.9	1.3	-0.9	-2.4	0.5
C-section	Mar 2020	-17.6	-51.1	15.9						
C-section	Apr 2020	-5.0	-36.3	26.3						
C-section	May 2020	-10.1	-42.0	21.8						
C-section	Jun 2020	-3.5	-34.7	27.7						
C-section	Jul 2020	-7.1	-38.6	24.4						
C-section	Aug 2020	-22.9	-58.0	12.2						
C-section	Sep 2020	8.6	-23.2	40.5						
C-section	Oct 2020	-10.3	-42.2	21.6						
C-section	Nov 2020	-11.3	-43.4	20.8						
C-section	Dec 2020	-29.2	-66.6	8.2						
C-section	Total Covid Period	-10.6	-25.6	4.3						
Measles vaccination	Mar 2020	-4.6	-9.3	0.1	-8.8	-18.4	0.7	-3.7	-7.4	0.1
Measles vaccination	Apr 2020	0.7	-4.0	5.4	1.4	-7.6	10.4	0.6	-3.2	4.3
Measles vaccination	May 2020	3.9	-0.8	8.6	7.5	-1.9	17.0	3.1	-0.6	6.9
Measles vaccination	Jun 2020	7.5	2.7	12.3	14.4	3.9	24.9	6.0	2.1	9.8
Measles vaccination	Jul 2020	-3.1	-7.8	1.6	-6.0	-15.3	3.2	-2.5	-6.2	1.3
Measles vaccination	Aug 2020	-6.3	-11.1	-1.6	-12.2	-22.2	-2.2	-5.1	-8.8	-1.3
Measles vaccination	Sep 2020	-0.5	-5.2	4.2	-1.0	-10.0	8.0	-0.4	-4.1	3.3
Measles vaccination	Oct 2020	-0.8	-5.5	3.9	-1.5	-10.5	7.5	-0.6	-4.3	3.1
Measles vaccination	Nov 2020	-5.4	-10.1	-0.7	-10.3	-20.1	-0.6	-4.3	-8.0	-0.5
Measles vaccination	Dec 2020	-5.8	-10.5	-1.0	-11.1	-20.9	-1.2	-4.6	-8.4	-0.8
Measles vaccination	Total Covid Period	-1.4	-3.4	0.6	-2.8	-6.7	1.2	-1.1	-2.8	0.5
OPD	Mar 2020	-8.2	-18.7	2.4	-14.4	-33.6	4.7	-6.6	-15.2	2.0
OPD	Apr 2020	9.4	-1.3	20.1	16.7	-2.9	36.3	7.6	-1.0	16.3
OPD	May 2020	1.0	-9.6	11.6	1.8	-17.0	20.5	0.8	-7.8	9.4
OPD	Jun 2020	-5.5	-16.0	5.1	-9.7	-28.6	9.2	-4.4	-13.0	4.1
OPD	Jul 2020	-17.6	-28.3	-7.0	-31.2	-52.2	-10.2	-14.3	-23.0	-5.7
OPD	Aug 2020	-7.4	-17.9	3.2	-13.0	-32.1	6.0	-6.0	-14.6	2.6
OPD	Sep 2020	0.7	-9.9	11.3	1.2	-17.5	20.0	0.6	-8.0	9.1
OPD	Oct 2020	8.1	-2.6	18.7	14.3	-5.1	33.7	6.5	-2.1	15.2
OPD	Nov 2020	14.7	4.0	25.5	26.2	5.4	46.9	12.0	3.2	20.7
OPD	Dec 2020	22.0	11.0	33.0	39.1	16.2	62.0	17.8	9.0	26.7
OPD	Total Covid Period	1.7	-2.9	6.3	3.0	-5.2	11.2	1.4	-2.3	5.1
IPD	Mar 2020	-24.9	-62.6	12.7	-96.5	-388.2	195.3	-17.7	-44.3	8.8
IPD	Apr 2020	-47.4	-88.4	-6.4	-180.9	-673.0	311.1	-33.8	-62.1	-5.4
IPD	May 2020	-19.0	-56.1	18.1	-73.9	-315.7	168.0	-13.5	-39.8	12.7
IPD	Jun 2020	-10.1	-46.7	26.4	-39.6	-216.5	137.3	-7.2	-33.2	18.8
IPD	Jul 2020	-27.9	-65.9	10.1	-107.7	-425.4	210.0	-19.9	-46.6	6.9
IPD	Aug 2020	-40.7	-80.5	-0.8	-155.9	-587.9	276.1	-29.0	-56.7	-1.3
IPD	Sep 2020	-38.2	-77.6	1.2	-146.5	-556.0	263.0	-27.2	-54.7	0.3
IPD	Oct 2020	-42.1	-82.1	-2.0	-161.1	-605.4	283.3	-30.0	-57.8	-2.1
IPD	Nov 2020	-8.4	-44.9	28.2	-32.7	-199.5	134.1	-6.0	-31.9	20.0
IPD	Dec 2020	17.6	-19.9	55.1	69.8	-172.6	312.1	12.5	-14.0	38.9
IPD	Total Covid Period	-23.2	-40.3	-6.1	-79.8	-266.3	106.7	-16.7	-28.7	-4.7

Amouzou A, *et al. BMJ Global Health* 2022; 7:e008069. doi: 10.1136/bmjgh-2021-008069

TANZANIA

Indicator	Month/Period	National %change	National Lower bound	National Upper bound	Rural %change	Rural Lower bound	Rural Upper bound	Urban %change	Urban Lower bound	Urban Upper bound
ANC1	Mar 2020	5.6	3.5	7.7	5.6	3.5	7.8	5.4	3.3	7.5
ANC1	Apr 2020	1.6	-0.5	3.6	1.6	-0.5	3.7	1.5	-0.5	3.5
ANC1	May 2020	-15.6	-17.9	-13.3	-15.8	-18.2	-13.4	-15.1	-17.8	-12.4
ANC1	Jun 2020	5.8	3.7	7.9	5.9	3.7	8.0	5.6	3.5	7.7
ANC1	Jul 2020	-9.3	-11.4	-7.1	-9.4	-11.6	-7.2	-9.0	-11.2	-6.7
ANC1	Aug 2020	-3.8	-5.9	-1.7	-3.9	-6.0	-1.7	-3.7	-5.7	-1.6
ANC1	Sep 2020	2.7	0.6	4.8	2.8	0.7	4.9	2.6	0.6	4.7
ANC1	Oct 2020	-12.8	-15.0	-10.5	-12.9	-15.2	-10.6	-12.4	-14.8	-9.9
ANC1	Nov 2020	-1.1	-3.2	0.9	-1.2	-3.3	0.9	-1.1	-3.1	0.9
ANC1	Dec 2020	1.4	-0.7	3.4	1.4	-0.7	3.5	1.3	-0.7	3.3
**ANC1**	Total Covid Period	-2.6	-3.5	-1.6	-2.6	-3.5	-1.6	-2.5	-3.4	-1.5
ANC4	Mar 2020	-5.5	-8.9	-2.1	-5.6	-9.1	-2.1	-5.2	-8.4	-1.9
ANC4	Apr 2020	-3.1	-6.5	0.3	-3.1	-6.6	0.3	-2.9	-6.1	0.3
ANC4	May 2020	-6.5	-9.9	-3.1	-6.7	-10.2	-3.2	-6.2	-9.4	-2.9
ANC4	Jun 2020	-1.8	-5.2	1.6	-1.9	-5.3	1.6	-1.7	-4.9	1.5
ANC4	Jul 2020	-8.2	-11.6	-4.8	-8.4	-11.9	-4.9	-7.7	-11.0	-4.4
ANC4	Aug 2020	-10.2	-13.6	-6.7	-10.4	-14.0	-6.8	-9.6	-12.9	-6.2
ANC4	Sep 2020	-7.8	-11.2	-4.3	-7.9	-11.4	-4.4	-7.3	-10.6	-4.0
ANC4	Oct 2020	-16.0	-19.6	-12.4	-16.4	-20.1	-12.7	-15.1	-18.7	-11.5
ANC4	Nov 2020	-11.6	-15.1	-8.1	-11.9	-15.5	-8.3	-10.9	-14.3	-7.6
ANC4	Dec 2020	-12.7	-16.2	-9.2	-13.0	-16.6	-9.3	-11.9	-15.3	-8.5
ANC4	Total Covid Period	-8.2	-9.8	-6.7	-8.4	-10.0	-6.8	-7.7	-9.3	-6.2
Deliveries	Mar 2020	-2.0	-4.5	0.6	-2.2	-5.0	0.7	-1.5	-3.5	0.5
Deliveries	Apr 2020	-2.1	-4.6	0.5	-2.3	-5.1	0.5	-1.6	-3.6	0.4
Deliveries	May 2020	-1.3	-3.9	1.2	-1.5	-4.3	1.4	-1.0	-3.0	1.0
Deliveries	Jun 2020	-0.1	-2.7	2.4	-0.1	-3.0	2.7	-0.1	-2.1	1.9
Deliveries	Jul 2020	-3.0	-5.5	-0.4	-3.3	-6.2	-0.5	-2.3	-4.3	-0.3
Deliveries	Aug 2020	-6.9	-9.5	-4.3	-7.6	-10.5	-4.7	-5.4	-7.4	-3.3
Deliveries	Sep 2020	-9.4	-12.1	-6.8	-10.5	-13.4	-7.5	-7.4	-9.5	-5.3
Deliveries	Oct 2020	-15.6	-18.3	-12.8	-17.2	-20.4	-14.1	-12.2	-14.5	-9.9
Deliveries	Nov 2020	-14.6	-17.4	-11.9	-16.2	-19.3	-13.1	-11.5	-13.7	-9.2
Deliveries	Dec 2020	-14.1	-16.8	-11.4	-15.6	-18.7	-12.5	-11.0	-13.3	-8.8
Deliveries	Total Covid Period	-6.8	-8.0	-5.7	-7.5	-8.9	-6.2	-5.4	-6.4	-4.4
Penta1	Mar 2020	-3.4	-5.9	-0.8	-3.3	-5.8	-0.8	-3.5	-6.1	-0.8
Penta1	Apr 2020	-4.6	-7.2	-2.1	-4.6	-7.1	-2.0	-4.8	-7.4	-2.1
Penta1	May 2020	-6.2	-8.8	-3.7	-6.1	-8.7	-3.6	-6.4	-9.1	-3.7
Penta1	Jun 2020	5.0	2.5	7.6	5.0	2.5	7.5	5.2	2.5	7.9
Penta1	Jul 2020	-4.9	-7.4	-2.3	-4.8	-7.3	-2.3	-5.0	-7.7	-2.3
Penta1	Aug 2020	-2.8	-5.4	-0.3	-2.8	-5.3	-0.3	-2.9	-5.5	-0.3
Penta1	Sep 2020	4.4	1.9	7.0	4.4	1.9	6.9	4.6	1.9	7.3
Penta1	Oct 2020	-8.7	-11.3	-6.2	-8.6	-11.2	-6.1	-9.0	-11.8	-6.2
Penta1	Nov 2020	-7.9	-10.4	-5.3	-7.8	-10.3	-5.2	-8.1	-10.9	-5.4
Penta1	Dec 2020	-10.2	-12.8	-7.6	-10.1	-12.7	-7.5	-10.6	-13.4	-7.7
Penta1	Total Covid Period	-3.9	-5.0	-2.8	-3.9	-4.9	-2.8	-4.0	-5.2	-2.8
Penta3	Mar 2020	-2.8	-5.8	0.2	-2.8	-5.7	0.2	-2.8	-5.9	0.2
Penta3	Apr 2020	-5.2	-8.2	-2.2	-5.2	-8.2	-2.2	-5.3	-8.4	-2.2
Penta3	May 2020	-7.5	-10.5	-4.5	-7.4	-10.4	-4.4	-7.6	-10.8	-4.5
Penta3	Jun 2020	-1.3	-4.3	1.6	-1.3	-4.3	1.6	-1.4	-4.4	1.7
Penta3	Jul 2020	-9.1	-12.2	-6.1	-9.1	-12.1	-6.1	-9.3	-12.5	-6.1
Penta3	Aug 2020	-5.2	-8.2	-2.2	-5.2	-8.1	-2.2	-5.3	-8.4	-2.2
Penta3	Sep 2020	0.3	-2.7	3.3	0.3	-2.7	3.3	0.3	-2.7	3.3
Penta3	Oct 2020	-7.5	-10.5	-4.5	-7.4	-10.4	-4.4	-7.6	-10.7	-4.5
Penta3	Nov 2020	-7.7	-10.7	-4.7	-7.7	-10.7	-4.7	-7.9	-11.0	-4.7
Penta3	Dec 2020	-11.6	-14.6	-8.6	-11.5	-14.6	-8.5	-11.8	-15.1	-8.5
Penta3	Total Covid Period	-5.7	-7.0	-4.4	-5.7	-7.0	-4.4	-5.8	-7.2	-4.4
C-section	Mar 2020	-0.3	-5.5	4.9	-0.4	-8.4	7.6	-0.1	-2.8	2.5
C-section	Apr 2020	0.2	-5.0	5.4	0.4	-7.6	8.4	0.1	-2.5	2.7
C-section	May 2020	-1.3	-6.5	3.9	-2.0	-10.1	6.0	-0.7	-3.3	2.0
C-section	Jun 2020	4.2	-1.0	9.5	6.5	-1.6	14.7	2.1	-0.5	4.8
C-section	Jul 2020	1.4	-3.8	6.6	2.1	-5.9	10.2	0.7	-1.9	3.3
C-section	Aug 2020	-0.6	-5.8	4.6	-0.9	-8.9	7.1	-0.3	-2.9	2.3
C-section	Sep 2020	0.8	-4.4	6.0	1.3	-6.8	9.3	0.4	-2.2	3.0
C-section	Oct 2020	-7.3	-12.6	-2.1	-11.3	-19.7	-2.9	-3.7	-6.4	-1.0
C-section	Nov 2020	-2.3	-7.5	2.9	-3.6	-11.7	4.4	-1.2	-3.8	1.4
C-section	Dec 2020	-2.7	-7.9	2.5	-4.2	-12.3	3.9	-1.4	-4.0	1.3
C-section	Total Covid Period	-0.8	-3.0	1.4	-1.2	-4.6	2.2	-0.4	-1.5	0.7
Measles vaccination	Mar 2020	5.0	1.8	8.1	4.9	1.8	8.1	5.1	1.8	8.3
Measles vaccination	Apr 2020	-6.6	-9.7	-3.4	-6.5	-9.7	-3.4	-6.7	-10.0	-3.4
Measles vaccination	May 2020	0.4	-2.8	3.5	0.4	-2.7	3.5	0.4	-2.8	3.6
Measles vaccination	Jun 2020	4.3	1.2	7.5	4.3	1.1	7.4	4.4	1.1	7.6
Measles vaccination	Jul 2020	-11.8	-15.0	-8.6	-11.7	-14.9	-8.5	-12.0	-15.5	-8.5
Measles vaccination	Aug 2020	-11.9	-15.2	-8.7	-11.9	-15.1	-8.7	-12.2	-15.7	-8.7
Measles vaccination	Sep 2020	7.6	4.4	10.7	7.5	4.3	10.7	7.7	4.4	11.0
Measles vaccination	Oct 2020	-0.2	-3.3	3.0	-0.2	-3.3	2.9	-0.2	-3.4	3.0
Measles vaccination	Nov 2020	-5.1	-8.2	-1.9	-5.0	-8.2	-1.9	-5.2	-8.4	-1.9
Measles vaccination	Dec 2020	-7.5	-10.7	-4.3	-7.5	-10.6	-4.3	-7.7	-11.0	-4.3
Measles vaccination	Total Covid Period	-2.6	-3.9	-1.2	-2.6	-3.9	-1.2	-2.6	-4.0	-1.2
OPD	Mar 2020	12.7	8.0	17.4	16.1	9.8	22.3	8.0	5.0	11.0
OPD	Apr 2020	-5.5	-10.0	-1.0	-6.9	-12.7	-1.2	-3.5	-6.3	-0.6
OPD	May 2020	-22.3	-27.4	-17.3	-28.1	-35.3	-21.0	-14.1	-17.5	-10.7
OPD	Jun 2020	-3.0	-7.5	1.5	-3.8	-9.4	1.9	-1.9	-4.7	0.9
OPD	Jul 2020	-4.6	-9.1	-0.1	-5.8	-11.5	-0.1	-2.9	-5.8	-0.1
OPD	Aug 2020	-3.0	-7.5	1.5	-3.8	-9.4	1.9	-1.9	-4.7	0.9
OPD	Sep 2020	3.1	-1.4	7.6	3.9	-1.8	9.6	1.9	-0.9	4.8
OPD	Oct 2020	-6.0	-10.5	-1.5	-7.6	-13.4	-1.9	-3.8	-6.7	-1.0
OPD	Nov 2020	-2.9	-7.4	1.6	-3.6	-9.3	2.0	-1.8	-4.6	1.0
OPD	Dec 2020	-1.6	-6.1	2.8	-2.1	-7.7	3.6	-1.0	-3.8	1.8
OPD	Total Covid Period	-3.3	-5.2	-1.4	-4.2	-6.6	-1.7	-2.1	-3.3	-0.9
IPD	Mar 2020	-4.6	-8.8	-0.3	-5.8	-11.2	-0.4	-2.9	-5.5	-0.2
IPD	Apr 2020	-19.2	-23.9	-14.5	-24.3	-30.9	-17.8	-12.0	-15.0	-9.0
IPD	May 2020	-28.6	-33.8	-23.4	-36.3	-44.0	-28.5	-17.9	-21.4	-14.5
IPD	Jun 2020	-16.3	-20.8	-11.7	-20.6	-26.8	-14.4	-10.2	-13.1	-7.2
IPD	Jul 2020	-12.5	-16.9	-8.1	-15.9	-21.8	-10.0	-7.8	-10.6	-5.0
IPD	Aug 2020	-11.1	-15.4	-6.7	-14.1	-19.8	-8.3	-6.9	-9.7	-4.2
IPD	Sep 2020	-4.9	-9.2	-0.7	-6.2	-11.7	-0.8	-3.1	-5.7	-0.4
IPD	Oct 2020	-6.7	-10.9	-2.4	-8.4	-13.9	-3.0	-4.2	-6.8	-1.5
IPD	Nov 2020	-4.5	-8.8	-0.3	-5.7	-11.1	-0.3	-2.8	-5.5	-0.2
IPD	Dec 2020	-4.8	-9.0	-0.6	-6.1	-11.5	-0.7	-3.0	-5.6	-0.3
IPD	Total Covid Period	-11.1	-13.2	-9.0	-14.0	-17.0	-10.9	-7.0	-8.4	-5.6

UGANDA

		National			Rural			Urban			Mixed		
Indicator	Month/Period	%change	Lower bound	Upper bound	%change	Lower bound	Upper bound	%change	Lower bound	Upper bound	%change	Lower bound	Upper bound
ANC1	Mar 2020	-4.4	-8.0	-0.8	-5.0	-9.1	-1.0	-0.4	-0.7	-0.1	-1.4	-2.6	-0.3
ANC1	Apr 2020	-9.9	-13.5	-6.3	-11.3	-15.4	-7.2	-0.9	-1.2	-0.6	-3.2	-4.4	-2.0
ANC1	May 2020	-9.0	-12.6	-5.4	-10.3	-14.4	-6.2	-0.8	-1.1	-0.5	-2.9	-4.1	-1.7
ANC1	Jun 2020	11.6	8.0	15.3	13.3	9.1	17.5	1.0	0.7	1.4	3.8	2.6	5.0
ANC1	Jul 2020	-5.3	-8.9	-1.8	-6.1	-10.2	-2.0	-0.5	-0.8	-0.2	-1.7	-2.9	-0.6
ANC1	Aug 2020	-2.5	-6.1	1.1	-2.9	-7.0	1.2	-0.2	-0.5	0.1	-0.8	-2.0	0.3
ANC1	Sep 2020	-2.5	-6.0	1.1	-2.8	-6.9	1.3	-0.2	-0.5	0.1	-0.8	-2.0	0.4
ANC1	Oct 2020	-6.9	-10.5	-3.3	-7.9	-12.0	-3.8	-0.6	-0.9	-0.3	-2.2	-3.4	-1.1
ANC1	Nov 2020	2.4	-1.2	5.9	2.7	-1.4	6.8	0.2	-0.1	0.5	0.8	-0.4	1.9
ANC1	Dec 2020	8.2	4.6	11.8	9.4	5.3	13.5	0.7	0.4	1.1	2.7	1.5	3.9
**ANC1**	Total Covid Period	-1.8	-3.4	-0.3	-2.1	-3.8	-0.3	-0.2	-0.3	0.0	-0.6	-1.1	-0.1
ANC4	Mar 2020	-13.7	-18.9	-8.5	-14.8	-20.5	-9.2	-1.9	-2.6	-1.2	-5.9	-8.4	-3.5
ANC4	Apr 2020	-16.0	-21.2	-10.8	-17.3	-23.0	-11.7	-2.2	-2.9	-1.5	-7.0	-9.4	-4.5
ANC4	May 2020	-16.9	-22.1	-11.7	-18.3	-24.0	-12.6	-2.3	-3.1	-1.6	-7.4	-9.9	-4.8
ANC4	Jun 2020	-0.5	-5.7	4.6	-0.6	-6.2	5.0	-0.1	-0.8	0.6	-0.2	-2.5	2.0
ANC4	Jul 2020	2.3	-2.9	7.5	2.5	-3.1	8.1	0.3	-0.4	1.0	1.0	-1.2	3.2
ANC4	Aug 2020	0.0	-5.1	5.2	0.0	-5.6	5.6	0.0	-0.7	0.7	0.0	-2.2	2.3
ANC4	Sep 2020	11.6	6.3	16.8	12.5	6.8	18.2	1.6	0.9	2.3	5.0	2.6	7.4
ANC4	Oct 2020	8.7	3.4	13.9	9.4	3.7	15.0	1.2	0.5	1.9	3.7	1.4	6.1
ANC4	Nov 2020	7.8	2.6	13.0	8.5	2.8	14.1	1.1	0.4	1.8	3.4	1.1	5.7
ANC4	Dec 2020	8.7	3.4	13.9	9.4	3.7	15.0	1.2	0.5	1.9	3.8	1.4	6.1
ANC4	Total Covid Period	-0.8	-3.1	1.4	-0.9	-3.3	1.5	-0.1	-0.4	0.2	-0.4	-1.3	0.6
Deliveries	Mar 2020	-10.0	-14.0	-6.0	-11.3	-15.9	-6.7	-0.9	-1.2	-0.5	-3.7	-5.3	-2.1
Deliveries	Apr 2020	-11.5	-15.5	-7.4	-13.0	-17.7	-8.4	-1.0	-1.3	-0.6	-4.3	-5.9	-2.7
Deliveries	May 2020	-7.3	-11.3	-3.3	-8.2	-12.8	-3.7	-0.6	-1.0	-0.3	-2.7	-4.3	-1.2
Deliveries	Jun 2020	-5.4	-9.4	-1.4	-6.1	-10.6	-1.6	-0.5	-0.8	-0.1	-2.0	-3.5	-0.5
Deliveries	Jul 2020	-3.5	-7.5	0.5	-4.0	-8.5	0.6	-0.3	-0.6	0.0	-1.3	-2.8	0.2
Deliveries	Aug 2020	-2.0	-6.0	1.9	-2.3	-6.9	2.2	-0.2	-0.5	0.2	-0.8	-2.3	0.7
Deliveries	Sep 2020	-2.3	-6.3	1.7	-2.6	-7.2	1.9	-0.2	-0.5	0.1	-0.9	-2.4	0.6
Deliveries	Oct 2020	-1.0	-5.0	3.0	-1.2	-5.7	3.4	-0.1	-0.4	0.3	-0.4	-1.9	1.1
Deliveries	Nov 2020	4.2	0.2	8.2	4.8	0.2	9.4	0.4	0.0	0.7	1.6	0.1	3.1
Deliveries	Dec 2020	0.5	-3.5	4.5	0.6	-3.9	5.1	0.0	-0.3	0.4	0.2	-1.3	1.7
Deliveries	Total Covid Period	-3.8	-5.5	-2.1	-4.3	-6.2	-2.4	-0.3	-0.5	-0.2	-1.4	-2.1	-0.8
Penta1	Mar 2020	-15.5	-18.9	-12.1	-17.4	-21.2	-13.6	-2.2	-2.6	-1.7	-4.2	-5.1	-3.2
Penta1	Apr 2020	-16.4	-19.8	-13.0	-18.4	-22.3	-14.6	-2.3	-2.8	-1.8	-4.4	-5.3	-3.5
Penta1	May 2020	-11.1	-14.4	-7.7	-12.4	-16.2	-8.7	-1.5	-2.0	-1.1	-3.0	-3.9	-2.1
Penta1	Jun 2020	-2.5	-5.8	0.9	-2.8	-6.5	1.0	-0.3	-0.8	0.1	-0.7	-1.6	0.2
Penta1	Jul 2020	-5.4	-8.7	-2.1	-6.1	-9.8	-2.3	-0.7	-1.2	-0.3	-1.4	-2.3	-0.5
Penta1	Aug 2020	-11.9	-15.2	-8.5	-13.3	-17.1	-9.6	-1.6	-2.1	-1.2	-3.2	-4.1	-2.3
Penta1	Sep 2020	-3.6	-7.0	-0.3	-4.1	-7.8	-0.3	-0.5	-1.0	0.0	-1.0	-1.9	-0.1
Penta1	Oct 2020	-0.1	-3.4	3.2	-0.1	-3.9	3.6	0.0	-0.5	0.4	0.0	-0.9	0.9
Penta1	Nov 2020	-9.5	-12.9	-6.2	-10.7	-14.4	-6.9	-1.3	-1.8	-0.8	-2.5	-3.5	-1.6
Penta1	Dec 2020	2.6	-0.8	5.9	2.9	-0.9	6.6	0.4	-0.1	0.8	0.7	-0.2	1.6
Penta1	Total Covid Period	-7.2	-8.7	-5.8	-8.1	-9.7	-6.5	-1.0	-1.2	-0.8	-2.0	-2.4	-1.6
Penta3	Mar 2020	-13.8	-17.3	-10.3	-15.5	-19.4	-11.5	-2.0	-2.5	-1.5	-3.6	-4.6	-2.7
Penta3	Apr 2020	-21.5	-25.0	-17.9	-24.1	-28.1	-20.0	-3.1	-3.6	-2.6	-5.6	-6.6	-4.7
Penta3	May 2020	-11.9	-15.5	-8.4	-13.4	-17.4	-9.5	-1.7	-2.2	-1.2	-3.1	-4.1	-2.2
Penta3	Jun 2020	3.3	-0.2	6.8	3.7	-0.2	7.7	0.5	0.0	1.0	0.9	0.0	1.8
Penta3	Jul 2020	2.2	-1.3	5.7	2.5	-1.5	6.4	0.3	-0.2	0.8	0.6	-0.3	1.5
Penta3	Aug 2020	-6.3	-9.8	-2.8	-7.0	-11.0	-3.1	-0.9	-1.4	-0.4	-1.6	-2.6	-0.7
Penta3	Sep 2020	-0.8	-4.3	2.7	-0.9	-4.8	3.1	-0.1	-0.6	0.4	-0.2	-1.1	0.7
Penta3	Oct 2020	2.3	-1.2	5.9	2.6	-1.3	6.6	0.3	-0.2	0.8	0.6	-0.3	1.5
Penta3	Nov 2020	-9.0	-12.5	-5.5	-10.1	-14.0	-6.1	-1.3	-1.8	-0.8	-2.4	-3.3	-1.4
Penta3	Dec 2020	2.2	-1.3	5.7	2.5	-1.5	6.4	0.3	-0.2	0.8	0.6	-0.3	1.5
Penta3	Total Covid Period	-5.3	-6.8	-3.8	-5.9	-7.6	-4.2	-0.8	-1.0	-0.5	-1.4	-1.8	-1.0
C-section	Mar 2020	-19.2	-32.6	-5.9	-24.6	-41.9	-7.3	-1.0	-1.7	-0.3	-5.2	-8.9	-1.4
C-section	Apr 2020	-10.9	-24.0	2.2	-14.0	-30.8	2.9	-0.6	-1.2	0.1	-2.9	-6.5	0.6
C-section	May 2020	-10.7	-23.9	2.4	-13.7	-30.6	3.2	-0.6	-1.2	0.1	-2.9	-6.4	0.7
C-section	Jun 2020	-12.2	-25.4	1.1	-15.6	-32.6	1.4	-0.6	-1.3	0.1	-3.3	-6.9	0.3
C-section	Jul 2020	0.3	-12.8	13.3	0.3	-16.4	17.1	0.0	-0.7	0.7	0.1	-3.4	3.6
C-section	Aug 2020	-5.8	-18.8	7.3	-7.4	-24.1	9.4	-0.3	-1.0	0.4	-1.5	-5.1	2.0
C-section	Sep 2020	-2.6	-15.6	10.5	-3.3	-20.0	13.5	-0.1	-0.8	0.5	-0.7	-4.2	2.8
C-section	Oct 2020	-3.3	-16.3	9.8	-4.2	-20.9	12.6	-0.2	-0.8	0.5	-0.9	-4.4	2.6
C-section	Nov 2020	-14.8	-28.2	-1.5	-19.0	-36.2	-1.7	-0.8	-1.5	-0.1	-4.0	-7.7	-0.3
C-section	Dec 2020	-17.5	-30.8	-4.2	-22.4	-39.5	-5.2	-0.9	-1.6	-0.2	-4.7	-8.4	-1.0
C-section	Total Covid Period	-9.4	-15.0	-3.9	-12.0	-19.2	-4.8	-0.5	-0.8	-0.2	-2.6	-4.2	-1.0
Measles vaccination	Mar 2020	-18.1	-25.7	-10.5	-20.6	-29.3	-12.0	-2.2	-3.1	-1.2	-4.6	-6.5	-2.6
Measles vaccination	Apr 2020	-24.7	-32.4	-17.1	-28.2	-36.9	-19.5	-2.9	-3.9	-2.0	-6.3	-8.2	-4.3
Measles vaccination	May 2020	-6.7	-14.3	1.0	-7.6	-16.3	1.1	-0.8	-1.7	0.1	-1.7	-3.6	0.2
Measles vaccination	Jun 2020	4.9	-2.8	12.6	5.6	-3.2	14.3	0.6	-0.3	1.5	1.2	-0.7	3.2
Measles vaccination	Jul 2020	9.9	2.2	17.6	11.2	2.5	20.0	1.2	0.3	2.1	2.5	0.5	4.4
Measles vaccination	Aug 2020	-4.8	-12.4	2.8	-5.5	-14.2	3.2	-0.6	-1.5	0.3	-1.2	-3.1	0.7
Measles vaccination	Sep 2020	3.3	-4.4	11.0	3.8	-5.0	12.5	0.4	-0.5	1.3	0.8	-1.1	2.8
Measles vaccination	Oct 2020	9.8	2.1	17.5	11.2	2.4	20.0	1.2	0.3	2.1	2.5	0.5	4.4
Measles vaccination	Nov 2020	-3.6	-11.2	4.1	-4.1	-12.8	4.6	-0.4	-1.3	0.5	-0.9	-2.8	1.0
Measles vaccination	Dec 2020	5.0	-2.6	12.7	5.7	-3.0	14.5	0.6	-0.3	1.5	1.3	-0.7	3.2
Measles vaccination	Total Covid Period	-2.5	-5.8	0.7	-2.9	-6.6	0.9	-0.3	-0.7	0.1	-0.6	-1.5	0.2
OPD	Mar 2020	2.0	-20.7	24.7	2.3	-23.6	28.2	0.2	-1.8	2.2	0.7	-7.1	8.5
OPD	Apr 2020	-15.8	-38.4	6.7	-18.0	-43.7	7.6	-1.4	-3.4	0.6	-5.4	-13.2	2.4
OPD	May 2020	-19.7	-42.3	2.8	-22.5	-48.2	3.2	-1.7	-3.7	0.3	-6.8	-14.6	1.1
OPD	Jun 2020	-37.4	-59.9	-14.9	-42.5	-68.2	-16.9	-3.3	-5.3	-1.3	-12.8	-20.9	-4.8
OPD	Jul 2020	-11.4	-34.0	11.2	-13.0	-38.7	12.7	-1.0	-3.0	1.0	-3.9	-11.7	3.9
OPD	Aug 2020	-6.4	-29.0	16.2	-7.3	-33.1	18.5	-0.6	-2.5	1.4	-2.2	-10.0	5.6
OPD	Sep 2020	0.3	-22.4	23.0	0.3	-25.5	26.2	0.0	-2.0	2.0	0.1	-7.7	7.9
OPD	Oct 2020	-5.7	-28.3	16.9	-6.5	-32.3	19.3	-0.5	-2.5	1.5	-1.9	-9.7	5.8
OPD	Nov 2020	-3.6	-26.2	19.1	-4.1	-29.9	21.7	-0.3	-2.3	1.7	-1.2	-9.0	6.5
OPD	Dec 2020	7.9	-14.9	30.6	9.0	-16.9	34.9	0.7	-1.3	2.7	2.7	-5.1	10.5
OPD	Total Covid Period	-8.9	-18.2	0.5	-10.1	-20.7	0.5	-0.8	-1.6	0.1	-3.1	-6.4	0.2
IPD	Mar 2020	-10.3	-15.5	-5.1	-11.6	-17.5	-5.6	-0.9	-1.4	-0.4	-4.2	-6.7	-1.8
IPD	Apr 2020	-35.1	-41.4	-28.9	-39.5	-46.9	-32.1	-3.1	-3.6	-2.5	-14.6	-19.3	-9.8
IPD	May 2020	-29.0	-35.0	-23.1	-32.7	-39.6	-25.7	-2.5	-3.1	-2.0	-12.0	-16.1	-7.9
IPD	Jun 2020	-29.0	-35.0	-23.1	-32.7	-39.6	-25.7	-2.5	-3.1	-2.0	-12.0	-16.1	-7.9
IPD	Jul 2020	-26.6	-32.4	-20.8	-29.9	-36.7	-23.2	-2.3	-2.8	-1.8	-11.0	-14.8	-7.2
IPD	Aug 2020	-18.4	-23.8	-12.9	-20.7	-26.9	-14.4	-1.6	-2.1	-1.1	-7.6	-10.7	-4.5
IPD	Sep 2020	-10.2	-15.5	-5.0	-11.5	-17.4	-5.6	-0.9	-1.4	-0.4	-4.2	-6.7	-1.8
IPD	Oct 2020	-13.7	-19.0	-8.4	-15.5	-21.5	-9.4	-1.2	-1.7	-0.7	-5.7	-8.4	-3.0
IPD	Nov 2020	-5.2	-10.4	-0.1	-5.9	-11.7	-0.1	-0.5	-0.9	0.0	-2.2	-4.4	0.1
IPD	Dec 2020	-10.3	-15.5	-5.1	-11.6	-17.5	-5.7	-0.9	-1.4	-0.4	-4.3	-6.7	-1.8
IPD	Total Covid Period	-18.2	-20.9	-15.4	-20.4	-23.7	-17.1	-1.6	-1.9	-1.4	-7.7	-10.0	-5.3

ZAMBIA

		National			Rural			Urban		
**Indicator**	**Month/Period**	**%change**	**Lower bound**	**Upper bound**	**%change**	**Lower bound**	**Upper bound**	**%change**	**Lower bound**	**Upper bound**
ANC1	Mar 2020	-2.0	-6.4	2.3	-2.5	-7.7	2.8	-0.9	-2.8	1.0
ANC1	Apr 2020	-9.4	-13.8	-5.0	-11.3	-16.6	-6.1	-4.2	-6.1	-2.2
ANC1	May 2020	-21.9	-26.2	-17.5	-26.4	-31.7	-21.0	-9.7	-11.7	-7.8
ANC1	Jun 2020	6.5	2.1	10.9	7.9	2.6	13.2	2.9	1.0	4.8
ANC1	Jul 2020	-8.8	-13.1	-4.4	-10.6	-15.9	-5.3	-3.9	-5.9	-2.0
ANC1	Aug 2020	-6.1	-10.4	-1.7	-7.3	-12.6	-2.1	-2.7	-4.6	-0.8
ANC1	Sep 2020	2.7	-1.7	7.1	3.3	-2.0	8.5	1.2	-0.7	3.1
ANC1	Oct 2020	-10.1	-14.4	-5.7	-12.1	-17.4	-6.9	-4.5	-6.4	-2.5
ANC1	Nov 2020	-1.5	-5.9	2.8	-1.9	-7.1	3.4	-0.7	-2.6	1.3
ANC1	Dec 2020	3.6	-0.8	8.0	4.4	-0.9	9.6	1.6	-0.3	3.5
**ANC1**	Total Covid Period	-4.7	-6.5	-2.8	-5.6	-7.9	-3.4	-2.1	-2.9	-1.2
ANC4	Mar 2020	-15.8	-22.4	-9.2	-18.4	-26.2	-10.6	-7.8	-11.0	-4.5
ANC4	Apr 2020	-5.9	-12.5	0.6	-6.9	-14.6	0.8	-2.9	-6.2	0.3
ANC4	May 2020	-22.4	-29.1	-15.7	-26.1	-34.0	-18.2	-11.0	-14.3	-7.7
ANC4	Jun 2020	-13.2	-19.8	-6.5	-15.3	-23.1	-7.6	-6.5	-9.7	-3.2
ANC4	Jul 2020	-15.3	-21.9	-8.7	-17.8	-25.6	-10.1	-7.5	-10.8	-4.3
ANC4	Aug 2020	-26.2	-32.9	-19.4	-30.5	-38.5	-22.5	-12.9	-16.2	-9.6
ANC4	Sep 2020	-17.8	-24.5	-11.2	-20.8	-28.6	-13.0	-8.8	-12.0	-5.5
ANC4	Oct 2020	-22.1	-28.8	-15.4	-25.8	-33.7	-17.9	-10.9	-14.2	-7.6
ANC4	Nov 2020	-20.9	-27.6	-14.2	-24.4	-32.2	-16.5	-10.3	-13.6	-7.0
ANC4	Dec 2020	-21.7	-28.4	-15.0	-25.3	-33.2	-17.4	-10.7	-14.0	-7.4
ANC4	Total Covid Period	-17.5	-20.4	-14.7	-20.3	-23.8	-16.9	-8.8	-10.2	-7.3
Deliveries	Mar 2020	-13.7	-18.9	-8.5	-16.8	-23.3	-10.4	-5.8	-8.0	-3.6
Deliveries	Apr 2020	-16.4	-21.6	-11.2	-20.2	-26.6	-13.7	-7.0	-9.2	-4.8
Deliveries	May 2020	-17.5	-22.7	-12.3	-21.5	-28.0	-15.0	-7.4	-9.6	-5.2
Deliveries	Jun 2020	-16.4	-21.6	-11.2	-20.1	-26.6	-13.7	-7.0	-9.2	-4.8
Deliveries	Jul 2020	-16.0	-21.2	-10.8	-19.7	-26.1	-13.2	-6.8	-9.0	-4.6
Deliveries	Aug 2020	-13.0	-18.2	-7.9	-16.0	-22.4	-9.6	-5.5	-7.7	-3.3
Deliveries	Sep 2020	-14.2	-19.4	-9.1	-17.5	-23.9	-11.1	-6.0	-8.2	-3.9
Deliveries	Oct 2020	-13.8	-19.0	-8.6	-16.9	-23.4	-10.5	-5.9	-8.0	-3.7
Deliveries	Nov 2020	-14.3	-19.5	-9.1	-17.6	-24.0	-11.2	-6.1	-8.3	-3.9
Deliveries	Dec 2020	-11.6	-16.8	-6.4	-14.2	-20.6	-7.9	-4.9	-7.1	-2.7
Deliveries	Total Covid Period	-14.3	-16.5	-12.1	-17.5	-20.3	-14.7	-6.2	-7.1	-5.2
Penta1	Mar 2020	-5.5	-10.0	-0.9	-6.8	-12.5	-1.1	-2.3	-4.2	-0.4
Penta1	Apr 2020	-5.1	-9.7	-0.5	-6.3	-12.0	-0.7	-2.1	-4.0	-0.2
Penta1	May 2020	-3.0	-7.6	1.6	-3.7	-9.4	2.0	-1.2	-3.1	0.7
Penta1	Jun 2020	7.0	2.4	11.6	8.7	3.0	14.4	2.9	1.0	4.8
Penta1	Jul 2020	-6.0	-10.5	-1.4	-7.4	-13.1	-1.7	-2.5	-4.4	-0.6
Penta1	Aug 2020	-3.0	-7.6	1.6	-3.7	-9.4	1.9	-1.3	-3.1	0.6
Penta1	Sep 2020	2.7	-1.9	7.3	3.3	-2.4	9.0	1.1	-0.8	3.0
Penta1	Oct 2020	6.4	1.8	11.0	7.9	2.2	13.6	2.6	0.7	4.5
Penta1	Nov 2020	0.9	-3.6	5.5	1.2	-4.5	6.8	0.4	-1.5	2.3
Penta1	Dec 2020	-0.4	-5.0	4.2	-0.5	-6.2	5.2	-0.2	-2.1	1.7
Penta1	Total Covid Period	-0.6	-2.6	1.4	-0.7	-3.2	1.7	-0.2	-1.1	0.6
Penta3	Mar 2020	-4.9	-9.4	-0.3	-6.0	-11.7	-0.4	-2.0	-3.9	-0.1
Penta3	Apr 2020	-7.0	-11.5	-2.4	-8.6	-14.3	-3.0	-2.9	-4.8	-1.0
Penta3	May 2020	-2.1	-6.7	2.4	-2.7	-8.3	3.0	-0.9	-2.8	1.0
Penta3	Jun 2020	14.0	9.3	18.6	17.3	11.5	23.0	5.8	3.9	7.7
Penta3	Jul 2020	-2.6	-7.2	1.9	-3.2	-8.9	2.4	-1.1	-3.0	0.8
Penta3	Aug 2020	-0.9	-5.4	3.7	-1.1	-6.7	4.6	-0.4	-2.3	1.5
Penta3	Sep 2020	3.0	-1.6	7.6	3.7	-1.9	9.4	1.3	-0.6	3.2
Penta3	Oct 2020	6.2	1.6	10.8	7.7	2.0	13.4	2.6	0.7	4.5
Penta3	Nov 2020	4.0	-0.5	8.6	5.0	-0.7	10.7	1.7	-0.2	3.6
Penta3	Dec 2020	3.1	-1.4	7.7	3.9	-1.8	9.5	1.3	-0.6	3.2
Penta3	Total Covid Period	1.3	-0.7	3.3	1.6	-0.8	4.0	0.5	-0.3	1.4
C-section	Mar 2020	-20.0	-38.1	-1.9	-28.2	-55.2	-1.2	-6.5	-12.0	-0.9
C-section	Apr 2020	-23.1	-41.5	-4.7	-32.6	-60.5	-4.7	-7.4	-13.0	-1.8
C-section	May 2020	-22.0	-40.3	-3.7	-31.0	-58.6	-3.5	-7.1	-12.7	-1.5
C-section	Jun 2020	-16.8	-34.6	1.0	-23.7	-49.8	2.5	-5.4	-10.9	0.1
C-section	Jul 2020	-19.6	-37.7	-1.6	-27.7	-54.6	-0.8	-6.3	-11.9	-0.8
C-section	Aug 2020	-23.0	-41.4	-4.6	-32.4	-60.2	-4.5	-7.4	-13.0	-1.8
C-section	Sep 2020	-20.4	-38.5	-2.2	-28.7	-55.8	-1.6	-6.6	-12.1	-1.0
C-section	Oct 2020	-18.5	-36.4	-0.5	-26.0	-52.6	0.6	-5.9	-11.5	-0.4
C-section	Nov 2020	-27.4	-46.4	-8.4	-38.6	-67.9	-9.3	-8.8	-14.5	-3.2
C-section	Dec 2020	-18.6	-36.6	-0.7	-26.3	-52.9	0.3	-6.0	-11.6	-0.4
C-section	Total Covid Period	-20.2	-29.2	-11.1	-28.0	-43.0	-13.0	-6.7	-9.2	-4.2
Measles vaccination	Mar 2020	-12.5	-18.2	-6.8	-15.8	-23.1	-8.6	-4.9	-7.1	-2.6
Measles vaccination	Apr 2020	-13.6	-19.3	-7.9	-17.3	-24.6	-10.0	-5.3	-7.6	-3.1
Measles vaccination	May 2020	-7.3	-13.0	-1.6	-9.3	-16.6	-2.1	-2.9	-5.1	-0.6
Measles vaccination	Jun 2020	1.7	-4.0	7.5	2.2	-5.1	9.5	0.7	-1.6	2.9
Measles vaccination	Jul 2020	-16.5	-22.2	-10.7	-20.9	-28.2	-13.6	-6.5	-8.7	-4.2
Measles vaccination	Aug 2020	-2.4	-8.1	3.3	-3.0	-10.3	4.3	-0.9	-3.2	1.3
Measles vaccination	Sep 2020	-5.3	-11.0	0.4	-6.7	-13.9	0.6	-2.1	-4.3	0.2
Measles vaccination	Oct 2020	4.7	-1.0	10.5	6.0	-1.3	13.3	1.9	-0.4	4.1
Measles vaccination	Nov 2020	5.5	-0.3	11.2	7.0	-0.4	14.3	2.1	-0.1	4.4
Measles vaccination	Dec 2020	-9.1	-14.8	-3.4	-11.6	-18.9	-4.3	-3.6	-5.8	-1.3
Measles vaccination	Total Covid Period	-5.4	-7.8	-3.0	-6.9	-9.9	-3.8	-2.1	-3.1	-1.2
OPD	Mar 2020	10.7	5.0	16.4	13.3	6.1	20.5	4.4	2.0	6.7
OPD	Apr 2020	5.0	-0.7	10.6	6.2	-0.8	13.2	2.0	-0.3	4.4
OPD	May 2020	-7.5	-13.1	-1.8	-9.3	-16.3	-2.2	-3.1	-5.4	-0.7
OPD	Jun 2020	-10.0	-15.6	-4.3	-12.4	-19.5	-5.3	-4.1	-6.4	-1.8
OPD	Jul 2020	-11.9	-17.6	-6.2	-14.8	-21.9	-7.7	-4.9	-7.2	-2.6
OPD	Aug 2020	-14.9	-20.6	-9.2	-18.5	-25.8	-11.3	-6.1	-8.5	-3.8
OPD	Sep 2020	-3.6	-9.2	2.0	-4.5	-11.5	2.5	-1.5	-3.8	0.8
OPD	Oct 2020	1.4	-4.2	7.0	1.7	-5.3	8.7	0.6	-1.7	2.9
OPD	Nov 2020	6.9	1.2	12.5	8.5	1.5	15.6	2.8	0.5	5.1
OPD	Dec 2020	9.0	3.3	14.7	11.2	4.1	18.3	3.7	1.4	6.0
OPD	Total Covid Period	-1.5	-3.9	0.9	-1.9	-4.9	1.1	-0.6	-1.6	0.4

Amouzou A, *et al. BMJ Global Health* 2022; 7:e008069. doi: 10.1136/bmjgh-2021-008069

% CHANGE IN HOSPITAL ADMISSION OVER MARCH-DECEMBER 2020 BY FIRST LEVEL SUBNATIONAL AREA

Country and region	Indicator	%change	Lower bound	Upper bound
**BURKINA FASO**				
Boucle du Mouhoun	ipd	2.7	-3.8	9.2
Cascades	ipd	1.7	-2.4	5.8
Centre	ipd	1.0	-1.3	3.2
Centre Est	ipd	3.0	-4.2	10.2
Centre Nord	ipd	3.9	-5.9	13.8
Centre Ouest	ipd	3.3	-4.7	11.2
Centre Sud	ipd	5.4	-9.9	20.7
Est	ipd	2.1	-2.9	7.1
Hauts Bassins	ipd	2.7	-3.8	9.3
Nord	ipd	2.8	-4.0	9.6
Plateau central	ipd	2.5	-3.7	8.8
Sahel	ipd	2.0	-2.7	6.7
Sud Ouest	ipd	2.4	-3.3	8.1
**COTE D'IVOIRE**				
Abidjan 1	ipd	-1.5	-4.1	1.1
Abidjan 2	ipd	-3.8	-11.9	4.3
Agneby-Tiassa	ipd	-2.2	-6.5	2.0
Bafing	ipd	-8.6	-46.1	28.9
Bagoue	ipd	-11.3	-72.9	50.3
Belier	ipd	-2.6	-7.4	2.3
Bere	ipd	-11.8	-80.3	56.6
Bounkani	ipd	-13.4	-88.7	61.9
Cavally	ipd	-4.3	-14.2	5.7
Folon	ipd	-31.5	-597.6	534.7
Gbeke	ipd	-16.6	-110.1	76.8
Gbokle	ipd	-3.5	-12.3	5.3
Gontougo	ipd	-3.9	-12.4	4.6
Grands Ponts	ipd	-2.2	-6.3	2.0
Guemon	ipd	-2.3	-6.7	2.1
Gôh	ipd	-1.7	-4.9	1.4
Hambol	ipd	-2.2	-6.3	1.9
Haut Sassandra	ipd	-0.9	-2.4	0.6
Iffou	ipd	-3.2	-10.3	3.8
Indenie-Duablin	ipd	-2.8	-8.5	2.9
Kabadougou	ipd	-3.5	-12.5	5.5
Lôh-Djiboua	ipd	-1.9	-5.3	1.5
Marahoue	ipd	-2.7	-8.1	2.7
Me	ipd	-3.7	-11.6	4.3
Moronou	ipd	-3.4	-11.0	4.3
N'zi	ipd	-3.6	-11.9	4.7
Nawa	ipd	-3.5	-10.8	3.9
Poro	ipd	-2.4	-6.8	2.0
San Pedro	ipd	-1.3	-3.6	1.0
Sud-Comoe	ipd	-2.3	-6.7	2.0
Tchologo	ipd	-4.5	-16.5	7.5
Tonkpi	ipd	-2.8	-8.4	2.7
Worodougou	ipd	-5.0	-26.2	16.2

% CHANGE IN HOSPITAL ADMISSION OVER MARCH-DECEMBER 2020 BY FIRST LEVEL SUBNATIONAL AREA

Country and region	Indicator	%change	Lower bound	Upper bound
**GHANA**				
Ahafo	ipd	-20.2	-39.1	-1.3
Ashanti	ipd	-14.5	-18.5	-10.6
Bono	ipd	-13.8	-20.3	-7.4
Bono East	ipd	-15.3	-23.5	-7.1
Central	ipd	-17.8	-25.9	-9.8
Eastern	ipd	-20.7	-29.7	-11.7
Greater Accra	ipd	-22.2	-33.0	-11.5
North East	ipd	-21.4	-42.7	-0.1
Northern	ipd	-13.3	-18.4	-8.1
Oti	ipd	-23.7	-46.3	-1.1
Savannah	ipd	-33.1	-80.1	13.9
Upper East	ipd	-18.1	-27.8	-8.3
Upper West	ipd	-14.9	-22.7	-7.2
Volta	ipd	-16.3	-23.6	-9.0
Western	ipd	-13.0	-18.4	-7.7
Western North	ipd	-19.8	-34.8	-4.8
**KENYA**				
Baringo	ipd	-41.4	-54.1	-28.7
Bomet	ipd	-49.9	-67.8	-31.9
Bungoma	ipd	-10.9	-12.6	-9.2
Busia	ipd	-21.5	-25.7	-17.3
Elgeyo Marakwet	ipd	-49.7	-67.6	-31.9
Embu	ipd	-44.2	-58.5	-29.8
Garissa	ipd	-46.1	-62.3	-29.8
Homa Bay	ipd	-25.1	-30.4	-19.7
Isiolo	ipd	-95.1	-157.6	-32.7
Kajiado	ipd	-27.2	-33.4	-21.0
Kakamega	ipd	-10.8	-12.5	-9.2
Kericho	ipd	-18.1	-21.4	-14.8
Kiambu	ipd	-8.7	-10.0	-7.4
Kilifi	ipd	-22.8	-27.4	-18.1
Kirinyaga	ipd	-20.5	-24.4	-16.6
Kisii	ipd	-15.2	-17.8	-12.7
Kisumu	ipd	-10.7	-12.4	-9.1
Kitui	ipd	-26.9	-32.9	-20.8
Kwale	ipd	-49.7	-67.6	-31.9
Laikipia	ipd	-71.1	-106.4	-35.8
Lamu	ipd	-124.2	-229.6	-18.8
Machakos	ipd	-18.1	-21.4	-14.8
Makueni	ipd	-33.0	-41.7	-24.4
Mandera	ipd	-144.2	-290.3	1.9
Marsabit	ipd	-87.6	-140.9	-34.3
Meru	ipd	-16.2	-19.0	-13.4
Migori	ipd	-16.6	-19.5	-13.7
Mombasa	ipd	-14.7	-17.2	-12.3
Muranga	ipd	-34.3	-43.3	-25.2
Nairobi	ipd	-5.4	-6.2	-4.6
Nakuru	ipd	-9.7	-11.1	-8.2
Nandi	ipd	-35.2	-44.7	-25.7
Narok	ipd	-41.9	-55.0	-28.9
Nyamira	ipd	-80.6	-125.6	-35.5
Nyandarua	ipd	-51.1	-69.8	-32.3
Nyeri	ipd	-21.5	-25.7	-17.3
Samburu	ipd	-103.3	-176.5	-30.1
Siaya	ipd	-23.8	-28.7	-18.8
Taita Taveta	ipd	-80.3	-125.0	-35.6
Tana River	ipd	-224.9	-568.9	119.0
Tharaka Nithi	ipd	-53.6	-74.1	-33.1
Trans Nzoia	ipd	-32.2	-40.3	-24.0
Turkana	ipd	-50.8	-69.5	-32.2
Uasin Gishu	ipd	-52.0	-71.4	-32.6
Vihiga	ipd	-34.4	-43.5	-25.2
Wajir	ipd	-98.4	-165.0	-31.9
West Pokot	ipd	-43.5	-57.4	-29.6

% CHANGE IN HOSPITAL ADMISSION OVER MARCH-DECEMBER 2020 BY FIRST LEVEL SUBNATIONAL AREA

Country and region	Indicator	%change	Lower bound	Upper bound
**MALI**				
Bamako	ipd	-7.7	-17.7	2.3
Gao	ipd	-9.4	-22.5	3.7
Kayes	ipd	-8.9	-20.5	2.6
Kidal	ipd	-10.7	-25.4	4.1
Koulikoro	ipd	-5.4	-12.2	1.5
Menaka	ipd	-10.0	-24.2	4.3
Mopti	ipd	-8.4	-19.2	2.5
Segou	ipd	-8.4	-19.3	2.5
Sikasso	ipd	-10.1	-23.2	3.1
Taoudenit	ipd	-12.4	-29.3	4.6
Tombouctou	ipd	-6.2	-14.3	1.8
**NIGER**				
Agadez	ipd	-31.7	-88.9	25.6
Diffa	ipd	-40.8	-140.0	58.4
Dosso	ipd	-16.6	-33.8	0.5
Maradi	ipd	-30.4	-77.2	16.5
Niamey	ipd	-7.7	-13.8	-1.5
Tahoua	ipd	-25.2	-54.2	3.9
Tillaberi	ipd	-43.2	-120.2	33.8
Zinder	ipd	-21.9	-46.3	2.5
**TANZANIA**				
Arusha	ipd	-10.4	-15.6	-5.1
Dar Es Salaam	ipd	-3.9	-5.0	-2.9
Dodoma	ipd	-11.2	-16.9	-5.5
Geita	ipd	-17.9	-34.3	-1.6
Iringa	ipd	-13.9	-24.6	-3.1
Kagera	ipd	-6.5	-8.6	-4.4
Katavi	ipd	-30.1	-80.0	19.7
Kigoma	ipd	-8.6	-12.0	-5.1
Kilimanjaro	ipd	-8.8	-12.6	-5.0
Lindi	ipd	-14.4	-25.0	-3.8
Manyara	ipd	-12.7	-20.4	-5.0
Mara	ipd	-11.2	-16.6	-5.8
Mbeya	ipd	-10.5	-15.9	-5.1
Morogoro	ipd	-9.4	-13.2	-5.5
Mtwara	ipd	-16.4	-27.6	-5.1
Mwanza	ipd	-10.4	-15.3	-5.4
Njombe	ipd	-11.1	-17.5	-4.7
Pwani	ipd	-18.4	-32.4	-4.3
Rukwa	ipd	-13.0	-23.6	-2.5
Ruvuma	ipd	-8.2	-11.3	-5.0
Shinyanga	ipd	-11.9	-19.3	-4.6
Simiyu	ipd	-22.3	-47.5	2.8
Singida	ipd	-13.5	-22.1	-4.8
Songwe	ipd	-17.3	-34.0	-0.7
Tabora	ipd	-12.6	-19.7	-5.5
Tanga	ipd	-12.9	-19.3	-6.4
**UGANDA**				
Central	ipd	-13.4	-17.9	-8.9
Eastern	ipd	-21.5	-31.0	-12.0
Northern	ipd	-20.5	-29.0	-12.0
Western	ipd	-17.3	-23.6	-10.9
