# Supplementary material for: Health service utilisation during the COVID-19 pandemic in sub-Saharan Africa in 2020: a multicountry empirical assessment with a focus on maternal, newborn and child health services
Source: BMJ Glob Health. 2022 May 2;7(5):e008069. doi: 10.1136/bmjgh-2021-008069 (PMC9062456; doi:10.1136/bmjgh-2021-008069)
Supplement: Supplementary data [file bmjgh-2021-008069supp002.pdf]

Reflexivity Statement

Domain	Question	Response
Study conceptualization	1. How does this study address local research and policy priorities?	The study was born out of an on-going country collaboration on strengthening the capacity of local researchers and government institutions in the analysis of routine health facility data to inform performance reviews of health sector plan in reproductive, maternal, newborn and child health services, through the assessment of the disruption of the on-going COVID-19 pandemic on the continuation of these services.
	2. How were local researchers involved in study design?	The Countdown country collaboration includes on-going data analysis activities with researchers from a public health institution in each the twelve countries, extended to representative from the Ministry of Health. Multi-country workshops were held with these participants, convened by the African Population Research Center (APHRC) to discuss the timeliness and usefulness of the study, its design, data needs and implementation. A total of eight virtual workshops were held.
Research management	3. How has funding been used to support the local research team(s)?	The local research teams had on-going grant from the Countdown to 2030 initiative, through APHRC. Additional funds were provided to each country to support additional effort for data compilation, cleaning, analysis, and virtual workshops
Data acquisition and analysis	4. How are research staff who conducted data collection acknowledged?	All country participants (researchers and government staff) are listed in the “Countdown to 2030 country collaboration on COVID-19” for group authorship
	5. How have members of the research partnership been provided with access to study data?	The data are owned by each country team. An Excel template was developed to standardize the data for analysis. Facilitators from high-income institutions had access to the data in Excel to assist the data cleaning and analysis. Standard statistical codes were provided to each country team to customize and use to run the analysis.
	6. How were data used to develop analytical skills within the partnership?	Each country team analyzed its data based on standard statistical codes provided to them. Each country customized the codes for analysis and interpretation of the results. Facilitators conducted on-going training to each team to run the codes and review the results.
Data interpretation	7. How have research partners collaborated in interpreting study data?	Facilitators from global and regional institutions in the Countdown collaboration worked with each country to conduct the analysis and review the results. The countries presented, interpreted, and discussed their results at the virtual multi-country workshops organized for this purpose
Drafting and revising for intellectual content	8. How were research partners supported to develop writing skills?	Each country team wrote a country report summarizing the findings of the study. They reviewed and commented an initial outline and draft of the cross-country summary that were provided to them, focusing on their country results and overall summaries. All authors in the participating countries further reviewed and approved the final version of manuscript.
	9. How will research products be shared to address local needs?	All country team prepared presentation and summary report of their findings for internal discussion. In addition, a data quality report was developed to further support data quality assessment and correction. Furthermore, the findings were shared with UNICEF country offices to enhance further local discussion and action. The individual reports were shared with Ministries of Health. Analytic collaborations focusing on improving and analyzing health facility data are continuing.
Authorship	10.How is the leadership,	All country teams members that participated in the analytical workshops

	contribution and ownership of this work by LMIC researchers recognised within the authorship?	are listed a co-author under a group authorship of “Countdown to 2030 country collaboration on COVID-19”, agreed upon by all contributors.
	10. How have early career researchers across the partnership been included within the authorship team?	All country teams members that participated in the analytical workshops are listed co-authors under a group authorship of “Countdown to 2030 country collaboration on COVID-19”, agreed upon by all contributors. This includes several early career researchers from the public health institutions in sub-Saharan Africa
	11. How has gender balance been addressed within the authorship?	No specific restriction was made on the authorship and all country teams members that participated in the analytical workshops are listed co-authors under a group authorship of “Countdown to 2030 country collaboration on COVID-19”, agreed upon by all contributors. The gender ratios vary by country teams, some more women, some more men, overall significant participation of women.
Training	12. How has the project contributed to training of LMIC researchers?	This publication came from a larger collaboration with countries on capacity strengthening for evidence and result generation to inform country annual performance reviews. Countries participate in training workshops and technical meetings. The workshops and meetings are usually planned as face to face gathering and exchange, but the COVID-19 has forced us to go virtual.
Infrastructure	13. How has the project contributed to improvements in local infrastructure	This project has not directly contributed to improvements in local infrastructure
	14. What safeguarding procedures were used to protect local study participants and researchers?	The data used are routine counts of health services utilization and do not include any study participant information.
